# The genus *Litophyton* Forskål, 1775 (Octocorallia, Alcyonacea, Nephtheidae) in the Red Sea and the western Indian Ocean

**DOI:** 10.3897/zookeys.567.7212

**Published:** 2016-02-22

**Authors:** Leen P. van Ofwegen

**Affiliations:** 1 Naturalis Biodiversity Center, P.O. Box 9517, 2300 RA Leiden, The Netherlands

**Keywords:** Cnidaria, Anthozoa, Alcyonacea, Nephthea, revision, new species

## Abstract

The *Litophyton* species of the Red Sea and the western Indian Ocean are revised, which includes species previously belonging to the genus *Nephthea*, which is synonymized with *Litophyton*. A neotype for both *Litophyton arboreum*, the type species of *Litophyton*, and *Nephthea chabrolii*, the type species of *Nephthea*, are designated. The new species *Litophyton curvum* **sp. n.** is described and depicted, and a key to all *Litophyton* species is provided. Of the 26 species previously described from the western Indian Ocean and Red Sea, 13 species are considered valid and 13 have been synonymized or placed in other genera.

## Introduction

This is the second in a series of publications in which nephtheid genera are revised. The first (Ofwegen 2005) dealt with the genus *Chromonephthea*, erected for *Nephthea* species with coloured sclerites. In their paper dealing with a molecular approach of nephtheid taxonomy, Ofwegen and Groenenberg (2007) already stated that in a morphologically based revision of *Nephthea* the genus should be synonymized with *Litophyton*. This is the first part of such a morphologically based revision, dealing only with species from the the western Indian Ocean and Red Sea, in which both genera are synonymized based on the molecular and morphological data mentioned by Ofwegen and Groenenberg (2007). The material of the type species of both *Litophyton*, *Litophyton arboreum* Forskål, 1775, and *Nephthea*, *Nephthea chabrolii* Andouin, 1828, is lost. Both of these species were originally described from the Red Sea and the examination of extensive Red Sea material allowed the selection of neotypes for them. As the original descriptions of these two species revealed hardly any characters, the neotype selections were based on specimens that could not be allocated to any other species known from the Red Sea and Indian Ocean in accordance with identifications of other authors.

*Nephthya savignyi* Ehrenberg, 1834 is at present the type species of the genus *Dendronephthya*, as designated by Utinomi (1954a). After examination of many specimens from the Red Sea referable to this species it proved to belong to *Litophyton*, making *Dendronephthya* a junior synonym of *Litophyton*. This finding was supported by the molecular study of McFadden et al. (2011) in which specimens identified in that publication as *Dendronephthya savignyii* grouped with *Nephthea* and *Litophyton*. A case will be submitted to the International Commission on Zoological Nomenclature (ICZN) to preserve the name *Dendronephthya*. Meanwhile the species will be cited as Litophyton ?savignyi.

Examination of the types of most species of *Litophyton* and *Nephthea* and examination of many recently collected specimens proved that species of this genus have restricted distributions and that therefore the revision can be split based on different geographic regions. The 17 nominal species of *Litophyton* (van Ofwegen 2015a) and 49 nominal species originally referred to *Nephthea* (van Ofwegen 2015b) at present recorded as occurring in the Indo-West Pacific will be dealt with in separate publications.

### Abbreviations

NBC
Naturalis Biodiversity Center (formerly Rijksmuseum van Natuurlijke Historie, RMNH) Leiden, The Netherlands 


NHMW
Naturhistorisches Museum Wien, Austria 


NTM
Museum and Art Gallery of the Northern Territory, Darwin, Australia 


UUZM
Uppsala University Zoological Museum, Uppsala, Sweden 


ZMB
Museum für Naturkunde der Humboldt-Universität, Berlin, Germany 


ZMH
Zoologisches Museum Hamburg, Germany 


ZMTAU
Zoological Museum, Department of Zoology, Tel Aviv University, 69978 Tel Aviv, Israel 


## Material and methods

For the technical terms used in the descriptions, see the glossary compiled by Bayer et al. (1983).

Four permanent microscope slides have been made for each specimen examined, which are kept at RMNH: (1) one slide of the sclerites from a number of polyps and part of branch, (2) one of the sclerites from the surface and interior of the top of the stalk, (3) one of the sclerites from the surface and interior of the base of the stalk, and (4) a slide of polyps made transparent to study the arrangement of the sclerites.

Sclerite drawings have been made using the permanent microscope slides. As most old museum specimens examined contained a large amount of broken sclerites, SEM images were produced of recently collected material.

All the *Nephthea* and *Litophyton* type specimens available have been re-examined. In addition, more recent material from the RMNH and ZMTAU collections has been included.

## Systematic part

### Class Anthozoa Ehrenberg, 1831 Subclass Octocorallia Haeckel, 1866 Order Alcyonacea Lamouroux, 1812 Family Nephtheidae Gray, 1862

#### Litophyton

Taxon classificationAnimaliaAlcyonaceaNephtheidae

Forskål, 1775

Litophyton Forskål, 1775: 139.
Ammothea Lamarck, 1816: 410.
Nephthee Savigny, 1817: pl. 2 fig. 5 (plates of the text of Andouin)
Nephthea Audouin, 1828: 49.
Nephthya Ehrenberg, 1834: 284.
Neptaea Blainville, 1834: 523.
Nephtya Van Beneden, 1867: 197.
Amicella Gray, 1869: 123.
Verrilliana Gray, 1869: 130.
Litophytum Kükenthal, 1903: 106.

##### Diagnosis.

Nephtheids with bushy and arborescent colonies. Polyps clustered at the end of the terminal branches, forming catkins. Polyps non-retractile, without or with supporting bundle, sometimes completely unarmed. Sclerites of surface layer of branches, stem and stalk are spindles and unilateral spinose spindles, the colony stalk also contains capstans and derivatives of capstans. Interior of the stalk has sparsely tuberculated spindles. Colonies zooxantellate.

##### Type species.

*Litophyton arboreum* Forskål, 1775, by monotypy.

##### Remarks.

Because of the synonymy of *Nephthea* with *Litophyton*, for many species a spelling emendation needed to be made to comply with ICZN Art. 31.2 in relation to gender agreement between generic and species names.

##### Characters used.

*Litophyton* species are known to have extreme intraspecific variation in colony shape and sclerites (Verseveldt 1973). For the Red Sea and Indian Ocean the number of nominal species is 26, but in the present study this number has been reduced to 13 valid species, including a new one, whereas 13 species have been synonymized or assigned to other genera (see below).

Colony shape did not prove to provide a reliably constant character. A good example is Litophyton ?savignyi, which may resemble some other *Litophyton* species but can also have a colony shape like that seen in some species of *Stereonephthya* (Figures 50–51).

The polyp armature showed some useful characters but some sclerite arrangements were observed in various species: *Litophyton chabrolii* (Figure 2B), *Litophyton laevis* (Figure 2A), *Litophyton simulatum* (Figure 2D), and *Litophyton striatum* (Figure 2C), Only one species, Litophyton ?savignyi had a projecting supporting bundle; three had small rodlets in the polyp stalk, *Litophyton arboreum*, *Litophyton curvum* and *Litophyton filamentosum*; two had rodlets in the polyp head, *Litophyton maldivensis* and *Litophyton viridis*; one lacked sclerites in the adaxial polyp part, *Litophyton bumastum* and the remaining five species had spindles all over the polyp, *Litophyton chabrolii*, *Litophyton laevis*, *Litophyton lanternarium*, *Litophyton simulatum*, and *Litophyton striatum*.

The sclerites of *Litophyton* species show a staggering morphological variation, with those in the polyp and stem and stalk surfaces varying most in shape. Notably, the shape of the stalk surface sclerites is different depending on the height on the stalk. The least variable sclerites are the spindles of the internal canals. As with the polyp armature some species have the same types of spindles, which limits the usefulness of these sclerites.

#### Species from the Red Sea and western Indian Ocean considered valid

1. *Litophyton acuticonicum* (Verseveldt, 1974), Red Sea
2. *Litophyton arboreum* Forskål, 1775 (type lost), Red Sea and Socotra
3. *Litophyton bumastum* (Verseveldt, 1973), Madagascar
4. *Litophyton chabrolii* (Andouin, 1828) (type lost), Red Sea
5. *Litophyton curvum* sp. n., Red Sea
6. *Litophyton filamentosum* (Verseveldt, 1973), Madagascar
7. *Litophyton laevis* (Kükenthal, 1913), Red Sea
8. *Litophyton lanternarium* (Verseveldt, 1973), Madagascar
9. *Litophyton maldivensis* (Hickson, 1905), Red Sea and Maldives
10. Litophyton ?savignyi (Ehrenberg, 1834), Red Sea and Madagascar
11. *Litophyton simulatum* (Verseveldt, 1970), Red Sea, Socotra and Chagos Archipelago
12. *Litophyton striatum* (Kükenthal, 1903), Red Sea, Socotra, Chagos Archipelago and Madagascar
13. *Litophyton viridis* (May, 1898), Red Sea and East Africa

#### Species from the Red Sea and western Indian Ocean considered invalid

1. *Nephthea aberrans* Verseveldt, 1968 = *Litophyton savignyi*
2. *Spongodes albida* Holm, 1894 = *Litophyton savignyi*
3. *Nephthya armata* Thomson & Henderson, 1906 = *Stereonephthya*
  BM 1912.2.25.12 (holotype) and BM 1933.3.13.148 (holotype fragment) were re-examined. Despite disintegrated sclerites, it was obvious that the species belongs to *Stereonephthya*
4. *Nephthea galbuloides* Verseveldt, 1973 = *Litophyton striatum*
5. *Nephthea tixierae* Verseveldt, 1968 = *Litophyton savignyi*
6. *Litophyton acutifolium* Kükenthal, 1913 = *Litophyton viridis*
7. *Litophyton crosslandi* Thomson & McQueen, 1908 = *Litophyton viridis*
8. *Nephthea elatensis* Verseveldt & Cohen, 1971= *Litophyton striatum*
9. *Nephthea hirsuta* Tixier-Durivault, 1966 = *Stereonephthya*
MNHN holotype was re-examined. Despite disintegrated sclerites it was obvious that the species belongs to *Stereonephthya*
10. *Nephthya jaegerskioeldi* Holm, 1904 = *Litophyton savignyi*
11. *Ammothea sanderi* May, 1899 = *Litophyton viridis*
12. *Ammothea stuhlmanni* May, 1898 = *Litophyton viridis*
13. *Nephthya zanzibarensis* Thomson & Henderson, 1906 = *Stereonephthya*
  BM 1933.3.13.150 (part of syntype) was re-examined. Despite disintegrated sclerites it was obvious that the species belongs to *Stereonephthya*

#### Key to the species of *Litophyton* from the Red Sea and western Indian Ocean

**Table:** 

1	Supporting bundle projecting	***Litophyton savignyi***
–	Supporting bundle not projecting	**2**
2	Polyp stalk with rodlets	**3**
–	Polyp stalk without rodlets	**5**
3	Internal spindles of the base of the stalk mostly with blunt ends	***Litophyton arboreum***
–	Internal spindles of the base of the stalk mostly with pointed ends	**4**
4	Base of stalk surface sclerites straight	***Litophyton filamentosa***
–	Base of stalk surface spindles curved	***Litophyton curvum***
5	Polyps without sclerites or mostly small rodlets	**6**
–	Polyps with spindles	**7**
6	Internal spindles of the base of the stalk heavily branched.	***Litophyton viridis***
–	Internal spindles of the base of the stalk not branched, several with blunt ends	***Litophyton maldivensis***
7	Adaxial side of polyps without sclerites	***Litophyton bumastum***
–	Adaxial side of polyps with sclerites	**8**
8	Internal spindles of the base of the stalk very small, most about 0.5 mm long or shorter	**9**
–	Internal spindles of the base of the stalk at least 1.0 mm long	**10**
9	Internal spindles of the base of the stalk slender, up to 0.15 mm wide, few in number	***Litophyton laevis***
–	Internal spindles of the base of the stalk wide, up to 0.25 mm wide, many present; spindles with blunt ends can be present	***Litophyton simulatum***
10	Larger internal spindles of the base of the stalk heavily branched	***Litophyton striatum***
–	Larger internal spindles of the base of the stalk not branched	**11**
11	Internal spindles of the base of the stalk up to 2.0 mm long	***Litophyton acuticonicum***
–	Internal spindles of the base of the stalk up to about 1.0 mm long	**12**
12	Internal spindles of the base of the stalk mostly unbranched, with regular tuberculation	***Litophyton chabrolii***
–	Internal spindles of the base of the stalk often branched, with irregular tuberculation	***Litophyton lanternarium***

#### Litophyton acuticonicum

Taxon classificationAnimaliaAlcyonaceaNephtheidae

(Verseveldt, 1974)

Figures 1H , 3 , 4 , 5 , 6 , 7 , 8 , 9 , 10 , 11

Nephthea acuticonica Verseveldt, 1974b: 28, figs 20–21, pl. 9 (El Kura’, Gulf of Aqaba, Red Sea); McFadden et al. 2011: 25.
Litophyton acutifolium ; Verseveldt 1974b: 25, figs 19–18 (Gulf of Aqaba, Red Sea).
Litophyton acuticonicum Not Litophyton acutifolium Kükenthal, 1913: 12 (= Litophyton viridis).
Nephthea striata ; Verseveldt 1974b: 2 (listed).

##### Material examined.

**RMNH Coel. 8920**, part of holotype, Red Sea, Gulf of Aqaba, El Kura, trawling, 14 September 1967, Hebrew Univ.-Smiths. Red Sea project, 118/SLR 533; **RMNH Coel. 12318**, Red Sea, Gulf of Aqaba, Ras Muhammad, depth 12 m, 6 September 1976, coll. Y. Benayahu; **ZMTAU Co 25862 2051**, Red Sea, Gulf of Suez, Shag Rock, depth 3–24 m, 14 July 1987, coll. Y. Benayahu; **ZMTAU Co 25867 1354**, Red Sea, South tip Sinai, Tiran St., depth 3–4 m, 25 June 1985, coll. Y. Benayahu; **ZMTAU Co 25969 2170**, Red Sea, South tip Sinai, Shab Mahmud, Beacon Rock, depth 27 m, 22 March 1988, coll. Y. Benayahu; **ZMTAU Co 26190**, Red Sea, South tip Sinai, Sharm el Maya, depth 6–12 m, 16 March 1981, coll. Y. Benayahu; **ZMTAU Co 26191**, Red Sea, Straits of Tiran, Ras Nazrani, 24 March 1981, coll. Y. Benayahu; **ZMTAU Co 26202**, Red Sea, Gulf of Aqaba Naqeb Shahin, depth 18 m, 15 June 1981, coll. Y. Benayahu; **ZMTAU Co 26206**, Red Sea, South tip Sinai, south of Naama, Amphores, depth 16–20 m, 7 June 1981, coll. Y. Benayahu; **ZMTAU Co 26215**, Red Sea, Gulf of Aqaba, Naqeb Shahin, depth 25 m, 29 June 1981, coll. Y. Benayahu; **ZMTAU Co 26220**, Red Sea, South tip Sinai, Naama Garden, depth 30 m, 30 June 1981, coll. Y. Benayahu; **ZMTAU Co 26239**, Red Sea.

##### Reassigned to the species.

**RMNH Coel. 8483**, identified by Verseveldt as *Litophyton acutifolium*, Red Sea, Elat, coll. M. Grasshoff; **RMNH Coel. 8916**, identified by Verseveldt as *Litophyton acutifolium*, Red Sea, Hebrew Univ.-Smith. Red Sea project, 49b/SLR L 388; **RMNH Coel. 8939**, identified by Verseveldt as *Nephthea striata*, Red Sea, Gulf of Aqaba, Fara ‘un Island, 27 June 1967, Hebrew Univ.-Smiths. Red Sea project, 2/SLR 45; **RMNH Coel. 8955**, identified by Verseveldt as *Litophyton acutifolium*, Red Sea, Gulf of Aqaba, Marsa el Muqeilba, 6 January 1968, Hebrew Univ.-Smith. Red Sea project 64/SLR 1156.

##### Diagnosis.

*Litophyton* with branched spindles in the surface layer of the base of the stalk and large internal spindles in the base of the stalk, up to 2.0 mm long, the largest not branched.

##### Distribution.

Red Sea: Gulf of Suez, Gulf of Aqaba.

##### Remarks.

The species is sufficiently described by Verseveldt (1974b: 28). Here I give drawings of a polyp (Figure 3A), polyp sclerites (Figure 3B), base stalk internal (Figures 3C, 4B) and base stalk surface sclerites (Figure 4A) of the type. For showing variation, SEM images of sclerites of ZMTAU Co 25867 (Figures 5–8) and ZMTAU Co 26239 (Figures 9–11) are also presented.

RMNH Coel. 8483, identified by Verseveldt as *Litophyton acutifolium*, shows the polyp armature and large interior spindles that are characteristic of this species.

RMNH Coel. 8916 and RMNH Coel. 8955, also both identified by Verseveldt as *Litophyton acutifolium*, show the large internal stalk sclerites of this species. However, the polyps show an armature more like *Litophyton viridis* (*Litophyton acutifolium* is synonymised with that species). This weak *Litophyton viridis* armature could represent intraspecific variation.

The species is characterized by the presence of branched spindles of the surface layer of the base of the stalk and the presence of large internal spindles in the base of the stalk, sometimes with blunt ends or branched. *Litophyton arboreum* also has internal spindles with blunt ends, but here the surface spindles of the base of the stalk are never branched. *Litophyton simulatum* can also have spindles with blunt ends but these are always less than 0.5 mm long, while in *Litophyton acuticonicum* they are up to 1.5 mm long. Sometimes also spindles with side branches are present in the interior of the base of the stalk (ZMTAU 26239, Figure 11c), similar to those of *Litophyton striatum* but that species never shows internal spindles of 2.0 mm long. It is noteworthy that these longer spindles with blunt end were not photographed with the SEM but were present in the microscope slides.

#### Litophyton arboreum

Taxon classificationAnimaliaAlcyonaceaNephtheidae

Forskål, 1775

**Figure 1.**
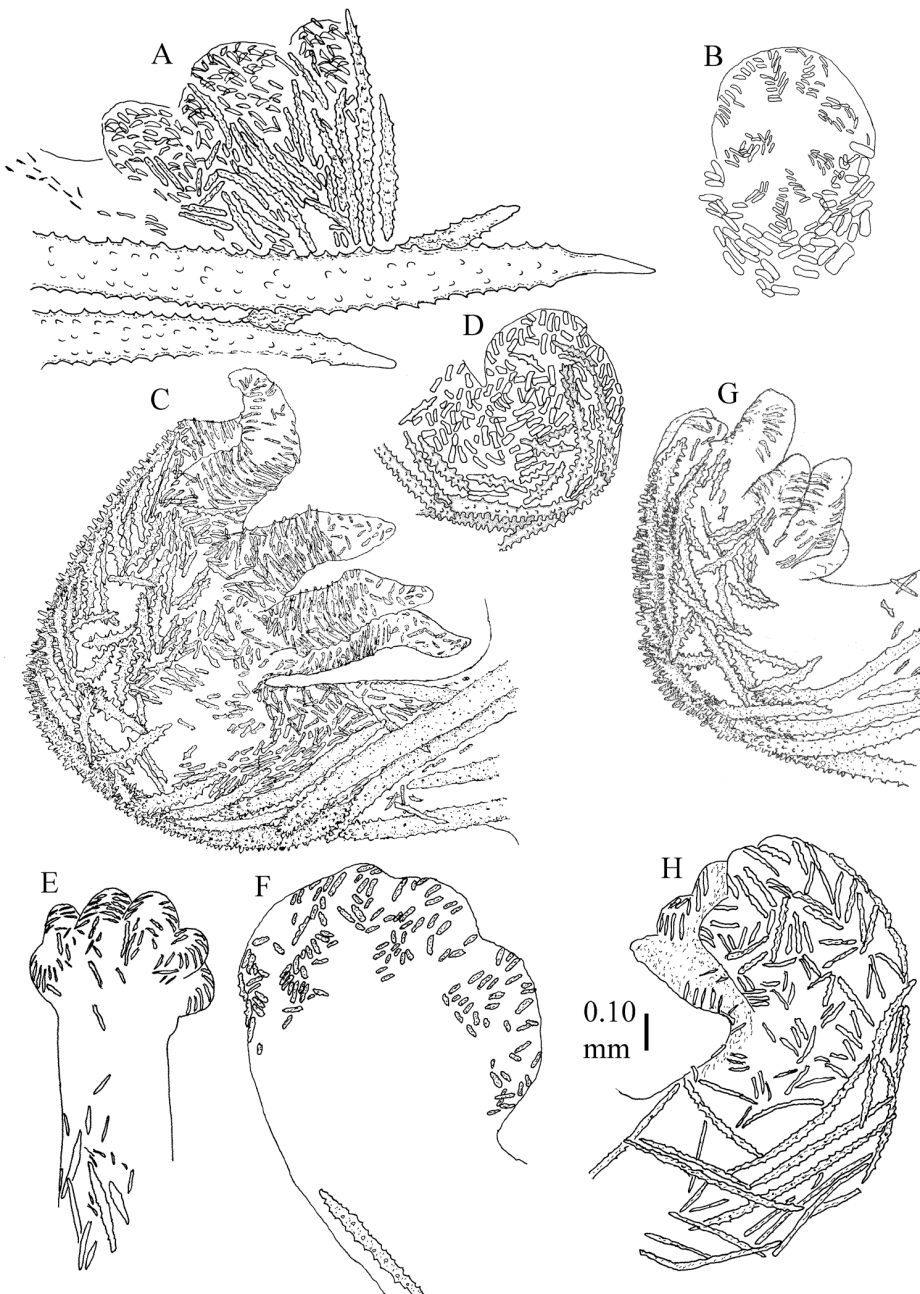
Polyp armatures. **A** *Litophyton savignyi* **B** *Litophyton arboreum* **C** *Litophyton filamentosum* **D** *Litophyton curvum* **E** *Litophyton maldivensis* **F** *Litophyton viridis* **G** *Litophyton bumastum* **H** *Litophyton acuticonicum*; all lateral views, except **B** which is an adaxial view. Figures **C, G** are from Verseveldt (1973).

Figures 1B , 12 , 13 , 14 , 15 , 16

Litophyton arboreum Forskål, 1775: 139 (Red Sea); Roxas 1933: 384 (in key only; discussion about synonymy); Verseveldt 1965: 33 (Red Sea).
Ammothea virescens Lamarck, 1816: 411; Savigny 1817: pl. 2 fig. 6; Blainville 1830: 486; 1834: 522; Ehrenberg 1834: 283 (listed); Gray 1869: 129; Haeckel 1876: pl. 1 fig. 9; Kükenthal 1869: 129.
Nephthea cordierii Audouin, 1828: 48 (Savigny's, pl. 2 fig. 6).
Neptaea inominata Blainville, 1830: 487 (Savigny's, pl. 2 fig. 6); 1834: 523.
Ammothea arborea Klunzinger, 1877: 31, pl. 2 fig. 4 (Red Sea); May 1899: 133.
Litophytum arboreum ; Kükenthal 1903: 124 (Red Sea); 1913: 12 (Red Sea); Thomson and McQueen 1908: 55 (Sudanese Red Sea); Shann 1912: 511, pl. 61 fig. 1 (reproduction of Savigny's, pl. 2 fig. 6).
Litophyton viride ; Bayer et al. 1983: pl. 17 fig. 121.
Litophyton arboreum Not Litophyton arboreum;Verseveldt 1966: 5, figs 1-2, pl. 1 (Sulawesi); Tixier-Durivault 1970a: 222 (Vietnam); 1972: 29 (Madagascar).

##### Material examined.

**ZMTAU Co 26246**, neotype, Red Sea, Gulf of Aqaba Eilat Marine lab, 12 m depth, 20 March 1978, coll. Y. Benayahu; **RMNH Coel. 8917**, Red Sea, Gulf of Suez, Abu Durba, coll. Hebrew Univ.-Smiths. Red Sea project; **RMNH Coel. 8918**, Red Sea, Gulf of Aqaba, 5 March 1972, coll. H. Schumacher; **RMNH Coel. 8919**, Red Sea, Gulf of Suez, Et Tur, depth 12 m, 1 January 1969, coll. Hebrew Univ.-Smiths. Red Sea project; **RMNH Coel. 8949**, Red Sea, Gulf of Suez, Et Tur, 20 September 1967, coll. Hebrew Univ.-Smiths. Red Sea project; **ZMTAU Co 25847**, two colonies, Red Sea, 1986–1987, coll. Y. Benayahu; **ZMTAU Co 25858**, Red Sea, Gulf of Suez, Shag Rock, depth 3–24 m, 14 July 1987, coll. Y. Benayahu; **ZMTAU Co 26234**, Red Sea, Gulf of Suez, El Bilaiyim lagoon, 24 August 1971, coll. D. Popper; **RMNH Coel. 42083**, Indian Ocean, Socotra, Ras Farun SW, sta. 207, sample 80, subtidal, 11 April 1999, coll. G. Reinicke, microscope slides only.

##### Reassigned to the species.

**RMNH Coel. 8941**, Red Sea, Gulf of Aqaba, Ophir Bay, 30 August 1967, coll. Hebrew Univ.- Smiths. Red Sea project (misidentified by Verseveldt as *Nephthea laevis*).

##### Removed from the species.

**RMNH Coel. 2218**, Indonesia, Sulawesi, 18 April 1978; **RMNH Coel. 17122**, Australia, Lodestone reef, July 1972, coll. G.R. Pettit (see remarks).

##### Diagnosis.

*Litophyton* with many internal spindles of the base of the stalk with blunt ends. The polyp stalk with scales.

##### Description.

The neotype is 5 cm high and 7.5 cm wide; the colony stalk is 2–3 cm high (Figure 12).

The polyps (Figure 13) are up to about 0.5 mm wide and high. Supporting bundle not projecting, composed of clavate spindles with simple, tall tubercles, outer side and one end thorny (Figure 14A). Length of these spindles is up to 0.7 mm. Polyp body sclerites irregularly arranged, the smallest are present adaxially, they are sparsely tuberculated spindles (Figure 14B); abaxially they merge into the smaller spindles of the supporting bundle and likewise have a thorny outer side (Figure 14C). The tentacles have rodlets up to 0.05 mm long (Figure 14D). The polyp stalk has scales up to 0.05 mm long (Figure 14E).

**Surface layer top of stalk.** Spindles, radiates, and derivatives of these, merging into unilaterally spinose spindles; all sclerites with simple tubercles (Figures 14F, 15A). The spindles are up to 0.3 mm long.

**Surface layer base of stalk.** Sclerites similar to those of the top of the stalk but with longer and sharper spines (Figure 15B–C).

**Interior base of stalk.** Spindles, up to 1.2 mm long, with simple sparse tubercles (Figure 16A–B). A few spindles have one or more side branches, many have one or two blunt ends. The smaller spindles are more often branched than the larger ones.

**Colour.** The colony is white.

##### Distribution.

Red Sea, Socotra.

##### Remarks.

The microscope slide of the stalk of ZMTAU Co 26234 only has internal sclerites of the stalk because the specimen has the surface layer missing.

RMNH Coel. 8917, 8918, and 8919 agree with the above description, although of RMNH Coel. 8918 no interior stalk microscope slide exists.

Two of the 14 microscope slides of RMNH Coel. 2218, from Indonesia, are missing, notably those of the interior stalk sclerites. The unilaterally spinose sclerites of the surface layer of the stalk have much higher spines than those of the neotype of *Litophyton arboreum*, and the slide with polyp sclerites also shows different sclerites, no polyp stalk scales at all. I regard this a misidentification.

RMNH Coel. 17122, from Australia, is clearly a misidentification, it has pointed interior sclerites in the base of the stalk.

*Litophyton arboreum* is characterized by having large spindles with blunt ends in the interior of the stalk. *Litophyton acuticonicum* and *Litophyton simulatum* also have this type of sclerites. *Litophyton acuticonicum* differs in having branched, unilaterally spinose spindles in the surface layer of the stalk, which are also twice as long as the unbranched spinose spindles of *Litophyton arboreum*. *Litophyton simulatum* also differs in having twice as long unilaterally spinose spindles in the surface layer of the stalk. Moreover, *Litophyton arboreum* has small oval scales in the polyp stalk, a type of sclerite not present in *Litophyton acuticonicum* and *Litophyton simulatum*.

#### Litophyton bumastum

Taxon classificationAnimaliaAlcyonaceaNephtheidae

(Verseveldt, 1973)

Figures 1G , 17 , 18 , 19

Nephthea bumasta Verseveldt, 1973: 98, figs 22-23 (Madagascar).

##### Material examined.

**RMNH Coel. 8045**, the holotype, Madagascar, Nosy Be, Pte. Lokobe, 8 m depth.

##### Diagnosis.

*Litophyton* with adaxial side of polyps without sclerites; interior stalk with pointed spindles up to 1.5 mm long; a few have blunt ends.

##### Distribution.

Madagascar.

##### Remarks.

The species is sufficiently described by Verseveldt (1973: 98). Here I show the drawing of the polyp as presented by Verseveldt (1973: fig. 22a). The colony, which was not shown by Verseveldt (Figure 17), together with SEM images of the sclerites (Figures 18–19).

*Litophyton bumastum* is the only *Litophyton* species in the western Indian Ocean described with the adaxial side of the polyps lacking sclerites.

#### Litophyton chabrolii

Taxon classificationAnimaliaAlcyonaceaNephtheidae

(Andouin, 1828)

**Figure 2.**
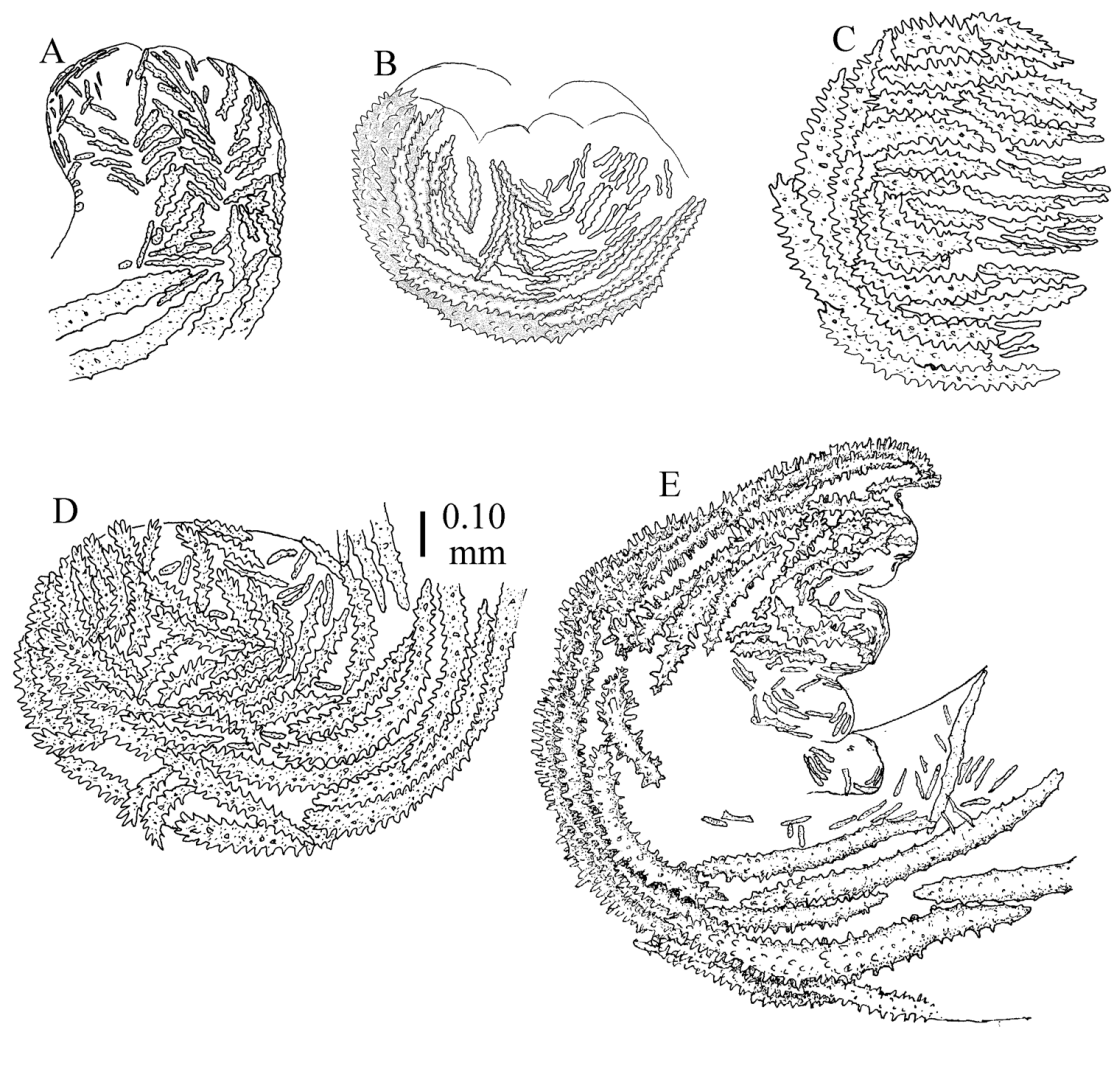
Polyp armatures **A** *Litophyton laevis* **B** *Litophyton chabrolii* **C** *Litophyton striatum* **D** *Litophyton simulatum* **E** *Litophyton lanternarium*; all lateral views. Figure **E** is from Verseveldt (1973).

Figures 2B , 20 , 21 , 22 , 23 , 24 , 25

Nephthea chabrolii Andouin, 1828:49 (explanation for Savigny's, “Description de l’Egypte ...”, 1817, pl. 2 fig. 5; ? Tixier-Durivault 1966: 273, figs 256–259 (Nosy Bé, Madagascar); Imahara 1996: 25 (listed).
Neptaea Savignyi ; Blainville 1830: 487 (listed); 1834: 523, pl. 88 fig. 6.
Litophyton chabrolii Not Nephthya Savignii; Ehrenberg 1834: 84; Dana 1846: 610 (= Litophyton savignyi).
Nephthya chabrolii ; Milne Edwards 1857: 128, pl. B1 figs 2a–2b (Red Sea); Kölliker 1864: 133 (listed); Klunzinger 1877: 33, pl. 2 fig. 5 (Red Sea); May 1899: 158; Kükenthal 1903: 157 (revision Nephthea); ?Thomson and Russell 1910: 183 (Salomon Reef, East Africa); Shann 1912: 511, pl. 61 figs 2–5, pl. 62 fig. 6 (reproduction of Savigny's, Nephthee plate.
Litophyton chabrolii Not Spongodes (Nephthya) chabrolii; Holm 1894: 25, pl. 2 figs 1–3 (Java Sea, Indonesia).
Litophyton chabrolii Not Spongodes (Nephthya) Chabrolii var. ternatana Kükenthal, 1895: 428 (Ternate, Indonesia).
Litophyton chabrolii Not Spongodes (Nephthya) Chabrolii var. molukkana Kükenthal, 1895: 428 (Ternate, Indonesia).
Litophyton chabrolii Not Nephthya chabrolii var. ternatana; Kükenthal 1896: 90.
Litophyton chabrolii Not Nephthya chabrolii var. moluccana; Kükenthal 1896: 91.
Litophyton chabrolii Not Nephthya chabrolii; Hickson and Hiles 1900: 500 (New Guinea); Thomson and Dean 1931: 83 (Malay Archipelago).
Litophyton chabrolii Not Nephthea chabrolii; Roxas 1933: 412 (Palawan, Philippines); Utinomi 1954b: 59, fig. 2 (Kii coast, Japan); Utinomi 1956: 233 (Palau); Verseveldt 1966: 14, figs 6–7, pl. 4 fig. 1 (Java, Indonesia); Tixier-Durivault 1970a: 225 (Vietnam); 1970b: 298 (New Caledonia); Utinomi 1971: 94, pl. 16 fig. 1 (Darwin, Australia); Verseveldt 1972: 457 (Eniwetok Atoll, Marshall Isl.; listed only, re-examined); 1977a: 3 (Carolines; listed only, re-examined); 1977c: 175 (Ellison Reef, Australia; listed only); Handayani et al. 1997 (Indonesia); Rao et al. 2000 (Gulf of Mannar, India); Lam and Morton 2008: 753, fig. E (Hong Kong = Chromonephthea sp.).

##### Material examined.

**ZMTAU Co 26244**, neotype, Red Sea, Gulf of Aqaba Wadi Magrash km 207, 20 July 1974, coll. Y. Benayahu (second specimen in the bottle is *Litophyton simulatum*); **RMNH Coel. 8956**, Red Sea, Gulf of Aqaba, Fara ‘un Isl., 7 January 1968, coll. Hebrew Univ.- Smiths. Red Sea project 65/SLR 1204 (identified as *Nephthea albida*); **RMNH Coel. 12364**, Red Sea, Gulf of Aqaba, Sharm el Sheikh, depth 10 m, 6 September 1976, coll. Y. Benayahu (identified as *Nephthea striata* by Verseveldt); **ZMTAU Co 26209**, Red Sea, Gulf of Aqaba, Shurat el Manqata, 9 November 1981, coll. Y. Benayahu; **ZMTAU Co 26228**, Red Sea, Gulf of Aqaba, Muqeibla, 4 June 1976, coll. Y. Benayahu; **ZMTAU Co 26229**, Red Sea, Gulf of Aqaba, Muqeibla, depth 4 m, 12 February 1976, coll. Y. Benayahu; **ZMTAU Co 26251**, Red Sea, South tip Sinai Ras Muhammed, depth 15 m, 21 April 1979, coll. Y. Benayahu.

##### Removed from the species.

**RMNH Coel. 2212**, voyage Boie & Macklot, nr. 83 (?Java); **RMNH Coel. 2216**, voyage Boie and Macklot, nr. 101 (?Java); **RMNH Coel. 2977**, Indonesia, Kei Islands, Tual anchorage, 12–16 December 1899, 22 m depth, Lithothamnion, sand and coral, reef exploration, dredge, Siboga sta. 258 (= *Chromonephthea intermedia* (Thomson and Dean, 1931)); **RMNH Coel. 2978**, Indonesia, Galewo Strait, off Salawatti Island; 1°42.5'S, 130°47.5'E, dredge, depth 32 m, sand and shells, 20 August 1899, Siboga sta 164 (= *Chromonephthea intermedia* (Thomson and Dean, 1931)); **RMNH Coel. 8921**, Red Sea, Gulf of Suez, Abu Zanima, 12 June 1968, coll. Hebrew Univ.-Smiths. Red Sea project (= *Litophyton simulatum*); **RMNH Coel. 8945**, Red Sea, Gulf of Aqaba, Marsa abu Zabad, 15 September 1967, coll. Hebrew Univ.-Smiths. Red Sea project (= *Litophyton simulatum*); **RMNH Coel. 8091**, Marshall islands, Eniwetok Atoll, in lagoon west of Eniwetok island, 5 m depth, 16 July 1969, coll. A.G. Humes; **RMNH Coel. 11767**, Ponape, shallow reef, about halfway Kolonia and Nanmatol, depth 1.5 m, coll. B. Jay Burreson; **RMNH Coel. 11944**, Indonesia, NW Ceram, Marsegoe Island, 2°59'30"S 128°03'30"E, depth 2 m, 15 May 1975, coll. A.G. Humes; **RMNH Coel. 10843**, Leti islands, Serwaru, coll. B. Tursch; **RMNH Coel. 10844**, Leti islands, Serwaru, coll. B. Tursch; **RMNH Coel. 11636**, Ellison Reef, seaward slope, 17°44'S, 146°24'E, depth 5 m, 8 January 1975, coll. R.N. Garrett; **RMNH Coel. 13161**, Australia, GBR, SE outer slope of John Brewer Reef, depth 15 m, 2–6 November 1976, coll. Terence Done; **RMNH Coel. 14119**, Australia, GBR, Lizard Island, between Bird and South island, depth 6–9 m, 14 February 1977, coll. H.K. Larson; **RMNH Coel. 24017**, Indonesia, W Sumatra, off shore of Sinyaru island, snorkelling, April 1994, coll. Ru Angelie Edrada; **ZMB 3589** [label: Spongodes chabrolii var. ternatana]; **ZMB 3590** [label: Spongodes chabrolii var. moluccana]; **ZMB 6764** [label: Nephthya chabrolii var. moluccana]; **ZMB 6765** [label: Nephthya chabrolii var. ternatana].

##### Diagnosis.

*Litophyton* with polyps with spindles. Internal spindles of the base of the stalk up to about 1.0 mm long, mostly unbranched and with very regular tuberculation.

##### Description.

The neotype is 4 cm high and 6.5 cm wide; the colony stalk is 1 cm high (Figure 20).

The polyps are up to about 0.5 mm wide and high (Figure 21). Supporting bundle not projecting, composed of spindles with simple or complex tubercles (Figure 22D). Length of these spindles is up to 1.2 mm. Polyp body sclerites irregularly arranged, the smallest are present adaxially (Figure 22B); abaxially they merge into the smaller spindles of the supporting bundle (Figure 22C). The tentacle sclerites resemble the smallest adaxial polyp sclerites (Figure 22A).

**Surface layer top of stalk.** Spindles, radiates, and derivatives of these, spindles, and unilaterally spinose spindles; sclerites with simple or complex tubercles (Figure 23). The spindles are up to 0.6 mm long.

**Surface layer base of stalk.** Sclerites similar to those of the top of the stalk but the unilaterally spinose sclerites with slightly longer spines (Figure 24).

**Interior base of stalk.** Spindles, up to 1.2 mm long, with simple, regular, sparse tubercles (Figure 25). Several spindles have one or more side branches, a few have one or two blunt ends. The smaller spindles are more often branched than the larger ones.

**Colour.** The colony is white.

##### Distribution.

Gulf of Aqaba.

##### Remarks.

The species resembles *Litophyton lanternarium* and *Litophyton simulatum* but differs in having mostly unbranched internal stalk spindles with very regular tuberculation.

It is noteworthy that Lam and Morton (2008) probably misidentified a specimen of *Chromonephthea* as they mentioned coloured specimens with coloured sclerites, characters of that genus and not of *Lithophyton*.

RMNH 2212, 2216 is the material from Indonesia described by Verseveldt (1966) as *Nephthea chabrolii*. It has similar polyp armature as the neotype here described. However, the internal stalk sclerites are branched, not present in any Red Sea specimens identifiable as *Litophyton chabrolii*. Likewise a number of RMNH specimens identified as *Nephthea chabrolii* from the Indo-Pacific and a few ZMB specimens from Indonesia (see removed from the species) all proved to be other species and therefore I have to conclude *Nephthea chabrolii* as here described has only been found in the Red Sea so far.

#### Litophyton curvum sp. n.

Taxon classificationAnimaliaAlcyonaceaNephtheidae

http://zoobank.org/A80D0522-AA9E-4C1A-BC67-CC04CD1EBE02

Figures 1D , 26 , 27 , 28 , 29 , 30 , 31 , 32

##### Material examined.

**ZMTAU Co 28555 (E167)**, holotype and seven paratypes, Eritrea, Dahlak Archipelago, Dur Ridgrig, depth 8 m, 15 October 1993, coll. Y. Benayahu; **paratypes: ZMTAU Co 25670 1873**, Red Sea, South tip Sinai, Shab el Utaf, depth 0–20 m, 11 July 1987, coll. Y. Benayahu; **ZMTAU Co 26223**, Red Sea, Gulf of Aqaba, 10 km south of Dahab, 24 July 1972, coll. L. Fishelson; **ZMTAU Co 26225**, Red Sea, South tip Sinai Ras um Sud, 11 April 1972, coll. Y. Benayahu; **ZMTAU Co 28549 (E241)**; Eritrea, Dahlak Archipelago, Sarad, depth 3 m, 17 October 1993, coll. Y. Benayahu; **ZMTAU Co 28552 (E261)**, Red Sea, Dahlak Archipelago, Daliacus, depth 3 m, 18 October 1993, coll. Y. Benayahu; **ZMTAU 32929**, Eritrea, Dahlak Archipelago, between Nocra Is. and Dahlak Is., southern entrance to the channel, 15°41.36'N, 39°56.08'E, depth 0–5 m, 14 February 2005, coll. Y. Benayahu; **ZMTAU Co 32964**, Eritrea, Dahlak Archipelago, Shumma Is., 15°32.00'N, 40°00.00'E, depth 8–12 m, 16 February 2005, coll. Y. Benayahu.

##### Removed from the species.

**RMNH 12317**, (identified as *Nephthea striata* by Verseveldt, Red Sea, Gulf of Aqaba, Sharm el Sheikh, depth 30 m, 7 September 1976, coll. Y Benayahu.

##### Diagnosis.

*Litophyton* with the internal spindles of the base of the stalk mostly with pointed ends. Polyp stalk with scales. Surface layer of the stalk with straight and curved sclerites.

##### Description.

The flabby holotype ZMTAU Co 28555 is 5.5 cm long and wide (Figure 26A); the colony is bent to one side.

The polyps are up to about 0.5 mm wide and 0.6 mm high (Figure 27). Supporting bundle not projecting, composed of spindles with simple tubercles, outer side and distal end with larger tubercles (Figure 28A). Length of these spindles is up to 1.0 mm. Polyp body sclerites irregularly arranged, the smallest are present adaxially (Figure 28B); abaxially they merge into the smaller spindles of the supporting bundle and have a thorny outer side (Figure 28C). The tentacle sclerites are small rodlets up to 0.1 mm long (Figure 28D). The polyp stalk has scales up to 0.05 mm long (Figure 28E).

**Surface layer top of stalk.** Radiates, derivatives of these, spindles and unilaterally spinose spindles (Figure 29A–B); the latter up to 0.6 mm long.

**Surface layer base of stalk.** Radiates, derivatives of these, spindles and unilateral spinose spindles (Figures 29C, 30A); the spindles and unilateral spinose spindles are up to 0.5 mm long; many are slightly curved.

**Interior base of stalk.** Spindles, up to 1.0 mm long, with widely spaced simple tubercles (Figure 30B–D); some spindles branched.

##### Etymology.

The Latin “curvum”, curve, curved object or line, refers to the curved spindles from the surface of the stalk.

##### Distribution.

Red Sea: Gulf of Aqaba, Dahlak Archipelago.

##### Remarks.

ZMTAU Co 26223, ZMTAU Co 26225 and ZMTAU Co 28552 are slightly different from the holotype. They show less compressed colony shapes (Figure 26D).

To show variation, the sclerites of ZMTAU Co 28552 are also presented (Figures 31–32).

The species can be confused with *Litophyton chabrolii* (Andouin, 1828), but that species has stiffer colonies, stronger polyp armature, and wider, more regular shaped internal stalk spindles. *Litophyton laevis* (Kükenthal, 1913) is also similar to this species, but lacks the curved spindles and unilateral spinose spindles in the surface layer of the base of the stalk. Moreover, both these species do not have the polyp stalk scales present in *Litophyton curvum*.

#### Litophyton filamentosum

Taxon classificationAnimaliaAlcyonaceaNephtheidae

(Verseveldt, 1973)

Figures 1C , 33 , 34 , 35

Nephthea filamentosa Verseveldt, 1973: 141, figs 24–25, pl. 6 (Tany Kely, near Nosy Bé, Madagascar).
Litophyton filamentosum Not Nephthea filamentosa; Ofwegen 1996: 209 (Papua New Guinea).

##### Material examined.

**RMNH Coel. 8046**, holotype, Tany Kely, Madagascar, 23 m depth; **RMNH Coel. 8047**, paratypes, Tany Kelly, Madagascar, 23 m depth.

##### Removed from the species.

**RMNH Coel. 12966**, Mililat Bay, Papua-New Guinea, 10 m depth; **RMNH Coel. 14596**, Laing I., Papua-New Guinea, 7 m depth.

##### Diagnosis.

*Litophyton* with the internal spindles of the base of the stalk mostly with pointed ends. Polyp stalk with scales, surface of the stalk with straight spindles and unilaterally spinose spindles.

##### Distribution.

Only known from the type locality Madagascar.

##### Remarks.

The species is sufficiently described by Verseveldt (1973: 141). Here I present the holotype colony shape (Figure 33) and SEM images of its sclerites (Figures 34–35).

The species mostly resembles *Litophyton curvum* but differs in having straight sclerites in the surface layer of the base of the stalk and very spiny, almost spheroidal, sclerites in the surface layer of the base of the stalk.

RMNH Coel. 12966 and RMNH Coel. 14596 are misidentifications. The specimens have no rodlets on the adaxial side of the polyp, as is the case in *Litophyton filamentosum*.

#### Litophyton laevis

Taxon classificationAnimaliaAlcyonaceaNephtheidae

(Kükenthal, 1913)

Figures 2A , 36 , 37 , 38 , 39 , 40 , 41

Nephthya laevis Kükenthal, 1913: 20, figs 9-13, pl. 2 fig. 5 (Red Sea, Jeddah).
Nephthea laevis ; Roxas 1933: 415; Verseveldt 1970: 219, figs 5–6, pl. 3 fig. 1 (Gulf of Suez, Et Tur).
Litophyton laevis Not Nephthea laevis; Verseveldt 1974b: 2 (Red Sea, Gulf of Aqaba, El Hamira; listed only; = Litophyton arboreum).

##### Material examined.

**ZMB 6818**, holotype, Kükth det., Rotes Meer, Djidda, Pola Exp.; **RMNH Coel. 6821**, Red Sea, Gulf of Suez, Et Tur, 6. July 1969, coll. L. Fishelson; **ZMTAU NS 8306**, Red Sea, Gulf of Suez, El-Bilaiyim lagoon, 24 August 1971, coll. D. Popper; **ZMTAU Co 25971**, Red Sea, Gulf of Suez, Jubal Is., Bluf point, depth 4 m, 24 March 1988, coll. Y. Benayahu; **ZMTAU Co 26126 3211**, Red Sea, Gulf of Suez, between Shaduan and Tawilla Is., 25 September 1989, coll. Y. Benayahu; **ZMTAU Co 26231**, Red Sea, Gulf of Suez, Ras Gahra, 26 September 1974, coll. Y. Benayahu; **ZMTAU Co 28550 (E258)**; Red Sea, Dahlak Archipelago, Daliacus; depth 3 m; 18 October 1993, coll. Y. Benayahu; **ZMTAU Co 28585 (E121)**, Red Sea, Dahlak Archipelago, Dur Gam, depth 3 m, 14 October 1993, coll. Y. Benayahu.

##### Removed from the species.

**RMNH 8941**, Red Sea, Gulf of Aqaba, Ophir Bay, 30 August 1967, coll. Hebrew Univ.- Smiths. Red Sea project (identified by Verseveldt as *Nephthea laevis* = *Litophyton arboreum*).

##### Diagnosis.

*Litophyton* with the internal spindles of the base of the stalk short and slender, up to 0.15 mm wide and 0.5 mm long.

##### Description.

The holotype is 8 cm high and 5 cm wide; the short colony stalk divides in several main stems shortly above its base (Figure 36A). Polyps are crowded at the end of the lobes arranged in globular to oval-shaped structures.

The polyps are up to about 0.7 mm high and 0.6 mm wide (Figure 37A). Supporting bundle not projecting, composed of spindles with simple tubercles, outer side and distal end with larger tubercles. Length of these spindles is up to 0.8 mm (Figure 37B). Polyp body sclerites irregularly arranged, the smallest are present adaxially; abaxially they merge into the smaller spindles of the supporting bundle (Figure 37C). The tentacle sclerites resemble the smallest adaxial polyp sclerites (Figures 37D, 39B–C).

**Surface layer top of stalk.** Rods and spindles, up to 0.45 mm long, with simple tubercles (Figure 37E).

**Surface layer base of stalk.** Radiates and derivatives of these, up to 0.15 mm long, with simple tubercles; a few are unilaterally spinose (Figure 38A). A few spindles and unilaterally spinose spindles are also present, up to 0.45 mm long, with simple tubercles.

**Interior base of stalk.** Spindles, up to 0.5 mm long, with simple sparse tubercles (Figure 38B). Several spindles have one or more side branches.

**Colour.** The colony is whitish.

##### Distribution.

Red Sea: Gulf of Suez, Dahlak Archipelago.

##### Remarks.

Kükenthal (1913) mentioned four specimens with his description, in Berlin I found only one specimen, labelled holotype, which is the same one that Kükenthal used in his description. He mentioned longer interior spindles, up to 1 mm long. I assume I missed the longer ones as only few interior spindles are present in the microscope slide made of the stalk of ZMB 6818.

The species can be confused with *Litophyton simulatum*, but the latter has wider, more branched internal spindles.

ZMTAU Co 26126 3211 has been used for SEM images of sclerites (Figures 39–41).

RMNH Coel. 8941 has spindles up to 1.3 mm long in the interior of the stalk, quite some of them with blunt ends and therefore I re-identified it as *Litophyton arboreum*.

#### Litophyton lanternarium

Taxon classificationAnimaliaAlcyonaceaNephtheidae

(Verseveldt, 1973)

Figures 2E , 42 , 43 , 44

Nephthea lanternaria Verseveldt, 1973: 147 (Madagascar).
Nephthea amentacea ; Verseveldt 1973: 91 (Madagascar).
Litophyton lanternarium Not Nephthya amentaceaStuder 1894: 123 (Sulu Islands)(see remarks).

##### Material examined.

**RMNH Coel. 8052**, holotype, Madagascar, east of Nosy Komba, near Nosy Bé, Bay of Tsimipaika, Banc de la Lanterne, depth 15 m, 26 July 1967, coll. A.G. Humes; **RMNH 8053**, paratype, same data as holotype.

##### Diagnosis.

*Litophyton* with the internal spindles of the base about 1.0 mm long, often branched, with irregular distribution of tubercles.

##### Distribution.

Only known from the type locality Madagascar.

##### Remarks.

The species is sufficiently described by Verseveldt (1973: 147). Here I give an image of the holotype (Figure 42) and present SEM images of its sclerites (Figures 43–44). Verseveldt mentioned and depicted (1973: fig. 29a) rodlets in the polyp stalk. I only noticed a few and with the SEM work they also did not stand out as they do in the species with many polyp stalk rodlets; only one is depicted by me (Figure 43E).

The species mostly resembles *Litophyton chabrolii* but differs in having internal spindles in the base of the stalk with irregular tuberculation; several spindles branched.

The specimens from Madagascar identified by Verseveldt as *Nephthea amentacea* (1973: 91) are very much like *Litophyton lanternarium*. They only differ in having longer spindles in the interior of the base of the stalk and these spindles having denser tuberculation. I could not find the type material of *Nephthea amentacea* and therefore the characters of that species remain unknown, but I consider it highly unlikely this species, which was described from the Sulu Islands, occurs in Madagascar anyway.

#### Litophyton maldivensis

Taxon classificationAnimaliaAlcyonaceaNephtheidae

(Hickson, 1905)

Figures 1E , 45 , 46 , 47 , 48 , 49

Eunephthya maldivensis Hickson, 1905: 824, fig. 12 (Maldives, Kolumadula Atoll); Kükenthal 1907: 380.
Litophyton maldivensis ; Hickson 1908: 173–176.

##### Material examined.

**BMNH 1962.7.20.123**, syntype; **BMNH 1962.7.20.124**, syntype; **ZMTAU Co 26249**, Red Sea, Gulf of Suez, Ras Gahra, depth 2 m, 19 November 1977, coll. Y. Benayahu; **ZMTAU Co 26221**, Red Sea, Gulf of Suez, A-Tur, 20 September 1967, coll. L. Fishelson; **ZMTAU Co 26252**, Red Sea, Gulf of Suez, Ras Gahra, depth 2 m, 20 November 1977, coll. Y. Benayahu; **ZMTAU Co 28548 (E262)**, Red Sea, Dahlak Archipelago, Daliacus; depth 3 m, 18 October 1993, coll. Y. Benayahu; **RMNH Coel. 42084**, Indian Ocean, Socotra, sta. 267, sample 86, subtidal, 15 April 1999, coll. G. Reinicke.

##### Diagnosis.

*Litophyton* with polyps with small rodlets. Internal spindles of the base of the stalk short, mostly unbranched, several with blunt ends.

##### Description.

The holotype is 3.5 cm high and 5 cm wide (Figure 45A).

The polyps have small rodlets and spindles, situated in the tentacles and both the lateral and abaxial parts of the polyp (Figure 46A–B). Length of the spindles up to 0.25 mm.

**Lobes.** Surface and interior with narrow spindles up to 0.5 mm long (Figure 46D).

**Surface layer top of stalk.** Radiates, derivatives of these, and spindles (Figure 46E); up to 0.30 mm long.

**Surface layer base of stalk.** Radiates, derivatives of these, spindles and unilateral spinose spindles (Figure 47A); the spindles and unilateral spinose spindles up to 0.25 mm long.

**Interior base of stalk.** Spindles with widely spaced simple tubercles (Figure 47B–D); some spindles branched; some smaller ones almost smooth; many with blunt ends. The interior spindles are up to 0.85 mm long.

##### Distribution.

Maldives, Red Sea, Socotra.

##### Remarks.

The characteristics of specimen BMNH 1962.7.20.124 agree with the description of Hickson (1905) of his single specimen. Therefore it is puzzling why nowadays the BMNH has two syntypes of *Litophyton maldivensis*. The other syntype, BMNH 1962.7.20.123, was also examined and shows characters of the genus *Scleronephthya*. Therefore, BMNH 1962.7.20.124 is here considered to be the holotype of *Litophyton maldivensis*.

The species can be confused with *Litophyton arboreum* Forskål, 1775, as that species has also many blunt spindles in the interior of the base of the stalk. But they are longer, have more regularly spaced tubercles and do not include smaller, smoother forms. Also the polyps are more strongly armed. *Litophyton maldivensis* can also be confused with specimens of *Litophyton simulatum* in terms of having short sclerites in the interior of the stalk. But *Litophyton simulatum*, like *Litophyton arboreum*, differs in lacking the smooth smaller internal spindles and having more strongly armed polyps. Moreover, it has many branched internal spindles.

SEM images of the sclerites of ZMTAU Co26249 (Figure 45B) are also presented (Figures 48–49). The polyp body sclerites of ZMTAU Co26249 (Figure 48B) are different from the rodlets of the holotype (Figure 46C), and the sclerites from the top of stalk surface (Figure 48D) are different to those shown for the holotype in Figure 46E. I consider these difference intraspecific variation.

#### Litophyton ?savignyi

Taxon classificationAnimaliaAlcyonaceaNephtheidae

(Ehrenberg, 1834)

Figures 1A , 50 , 51 , 52 , 53 , 54 , 55 , 56 , 57 , 58

Nephthya Savignyi Ehrenberg, 1834: 284 (Red Sea).
Spongodes Savignyi ; Klunzinger 1877: 35 (Koseir).
Dendronephthya savignyi ; Kükenthal 1905: 528. (Red Sea, Koseir, Tor); McFadden et al. 2011: 25.
Spongodes albida Holm, 1894: 30 (Red Sea, Gulf of Suez).
Nephthya albida ; Kükenthal 1903: 160; Thomson and Mcqueen 1908: 59.
Litophyton ?savignyi Not Nephthya albida; Thomson and Dean 1931: 82 (Indonesia).
Litophyton ?savignyi Not Nephthea albida; Roxas 1933: 413; Verseveldt 1966: 9 (Indonesia); Tixier-Durivault 1970b: 298 (New Caledonia); Verseveldt 1974a: 96 (New Caledonia, listed); 1976: 499; 1977c: 175 (John Brewer Reef, Townsville, Qld, Australia; listed only); 1978: 50 (Pacific); Imahara 1991: 74 (Kerama Islands, Ryukyu Isl., Japan); 1996: 25 (listed); Ofwegen 1996: 209 (Papua New Guinea).
Nephthya jaegerskioeldi Holm, 1904: 6 (Red Sea, Tor).
Nephthea aberrans Verseveldt, 1968: 54 (Tany Kely, near Nosy Bé, Madagascar); 1973: 96 (Tany Kely, near Nosy Bé, Madagascar; re-description).
Nephthea tixierae Verseveldt, 1968: 55 (Nosy Ovy, Radama Is., Madagascar); 1973: 94 (Nosy Ovy, Radama Is., Madagascar; re-description); Tixier-Durivault 1972: 26 (Madagascar).

##### Material examined.

**NHMW 2407**, 2 specimens, Red Sea, Tor, Frauenfeld; **UUZM 417**, type *Nephthya jaegerskioeldi*, Red Sea, Tor, depth 0.5–0.65 m; **RMNH Coel. 3906**, *Nephthea aberrans*, holotype, Tany Kely, Madagascar, depth 10 m; **RMNH Coel. 3907**, *Nephthea tixierae*, holotype, Nosy Ovy, depth 8 m; **RMNH Coel. 8044**, paratype, Nosy Ovy, depth 8 m, 9; **RMNH Coel. 42087**, Egypt, Safaga, sample 44, 5 April 1997, coll. G. Reinicke; **ZMTAU Co 25685 1965**, Red Sea, South tip Sinai, Shab Mahmud, depth 0–21 m, 12 July 1987, coll. Y. Benayahu; **ZMTAU Co 25688 1971**, Red Sea, South tip Sinai, Shab Mahmud, 12 July 1987, coll. Y. Benayahu; **ZMTAU Co 25690 1979**, Red Sea, South tip Sinai, Shab Mahmud, 12 July 1987, coll. Y. Benayahu; **ZMTAU Co 25825 1489**, Red Sea, South tip Sinai, Shab Mahmud, depth 20–30 m, 9 July 1986, coll. Y. Benayahu; **ZMTAU Co 25829 1523**, Red Sea, South tip Sinai, Shab Mahmud, depth 20–30 m, 9 July 1986, coll. Y. Benayahu; **ZMTAU Co 25833 1606**, Red Sea, Gulf of Suez, Shag Rock, depth 0–20 m, 10 July 1986, coll. Y. Benayahu; **ZMTAU Co 25840 1782**, Red Sea, South tip Sinai, Tiran St., 13 July 1986, coll. Y. Benayahu; **ZMTAU Co 25861 2048**, Red Sea, Gulf of Suez, Shag Rock, depth 3–24 m, 14 July 1987, coll. Y. Benayahu; **ZMTAU Co 25878 1625**, Red Sea, Gulf of Suez, Shag Rock, depth 0–20 m, 10 July 1986, coll. Y. Benayahu; **ZMTAU Co 25983 2618**, Red Sea, Tiran Straits, Gordon andThomas reef, depth 12–16 m, 27 March 1988, coll. Y. Benayahu; **ZMTAU Co 26061 (2748)**, Red Sea, Gulf of Suez, Shag Rock, 6 October 1988, coll. Y. Benayahu; **ZMTAU Co 26062 (2750)**, Red Sea, Gulf of Suez, Shag Rock, depth 10 m, 6 October 1988, coll. Y. Benayahu; **ZMTAU Co 26065 (2788)**, two specimens, Red Sea, Gulf of Suez, Shag Rock, 7 October 1988, coll. Y. Benayahu; **ZMTAU Co 26068 (2830)**, Red Sea, Gulf of Suez, Shag Rock, depth 30 m, 7 October 1988, coll. Y. Benayahu; **ZMTAU Co 26115 (3149)**, Red Sea, Gulf of Suez, Tawilla Is., 24 September 1989, coll. Y. Benayahu; **ZMTAU Co 26131 (3236)**, Red Sea, Gulf of Suez, near Shaduan Is., 26 September 1989, coll. Y. Benayahu; **ZMTAU Co 26189**, Red Sea, Gulf of Aqaba, Ras Mamlakh, coll. Y. Benayahu, 12 March 1981; **ZMTAU Co 26198 (1046)**, Red Sea, Gulf of Aqaba, Dahab southern oasis, depth 8 m, 4 November 1981, coll. Y. Benayahu; **ZMTAU Co 26205 (1077)**, Red Sea, S tip of Sinai, S of Naama, “Amphores”, depth 16–20 m, 7. November 1981, coll. Y. Benayahu; **ZMTAU 26208 (1088)**, Red Sea, Gulf of Aqaba, El Goz, depth 2–5 m, 8 November 1981, coll. Y. Benayahu; **ZMTAU Co 26210 (1089)**, Red Sea, Gulf of Aqaba, Shurat el Manqata, depth 3–6 m, 9 November 1981, coll. Y. Benayahu; **ZMTAU Co 26212 (1091)**, Red Sea, Gulf of Aqaba, Shurat el Manqata reef flat, 9 November 1981, coll. Y. Benayahu; **ZMTAU Co 26214 (1097)**, two specimens, Red Sea, Gulf of Aqaba, Naqeb Shahin, depth 25 m, 29 November 1981, coll. Y. Benayahu; **ZMTAU Co 26217 (1114)**, South tip Sinai, Sharm a Sheikh. “Amphores”, depth 20 m, 30 November 1981, coll. Y. Benayahu; **ZMTAU Co 26219 (1118)**, Red Sea, S tip of Sinai, Naama garden, depth 30 m, 30 November 1981, coll. Y. Benayahu; **ZMTAU 26233**, Red Sea, Tiran Is., Favel Bay lagoon, depth 1–2 m, 22 September 1981, coll. Kerman; **ZMTAU Co 26235**, Red Sea, Gulf of Suez, Ras Tanaka, 25 September 1974, coll. Y. Benayahu; **ZMTAU Co 26245 (327)**, Red Sea, Gulf of Aqaba, Taba km 179, depth 25 m, 9 October 1977, coll. Y. Benayahu; **ZMTAU Co 26247 (350)**, Red Sea, Gulf of Suez, Sheikh Riach, depth 5 m, 18 November 1977, coll. Y. Benayahu; **ZMTAU Co 26248 (389)**, Red Sea, Gulf of Suez, Sheikh Riach, depth 5 m, 18 November 1977, coll. Y. Benayahu; **ZMTAU 26250 (446)**, Red Sea, Gulf of Suez, Ras Gahra, depth 1 m, 29 November 1977, coll. Y. Benayahu; **ZMTAU Co 26253 (470)**, Red Sea, South tip Sinai, Marsa Khadamia, depth 30 m, 22 November 1977, coll. Y. Benayahu; **ZMTAU Co 26254 (17923)**, Red Sea, coll. Y. Benayahu; **ZMTAU NS 8295**, Red Sea, Gulf of Suez, Ras Kanisa, 20 October 1971, coll. Fishelson; **ZMTAU Co 30064**, Eritrea, *Heteroxenia* bed near Eucus island, 15°53.884'N, 39°53.141'E, depth 3.5 m, 29 April 1997, coll. Y. Benayahu; **ZMTAU Co 32965**, Eritrea, Dahlak Archipelago, Shumma Is., 15°32.00'N, 40°00.00'E, depth 8–12 m, 16 February 2005, coll. Y. Benayahu; **ZMTAU Co 34205**–**34207**, Red Sea, Gulf of Aqaba, North Oil Jetty Elat, 29°31.41'N, 34°56.14'E, depth 15.2 m, 26 July 2007, coll. Y Benayahu; Z**MTAU Co 34066**–**34067**, Red Sea, Gulf of Aqaba, Elat, 29°30.14'N, 34°55.075'E, depth 18.3–22.9 m, 23 July 2007, coll. Y. Benayahu.

##### Removed from the species.

**RMNH Coel. 8956**, Red Sea, Gulf of Aqaba, Fara ‘un Isl., 7 January 1968, coll. Hebrew Univ.- Smiths. Red Sea project 65/SLR 1204 (identified as *Nephthea albida* = *Litophyton chabrolii*).

##### Diagnosis.

*Litophyton* where the polyps have a projecting supporting bundle and make an acute angle with the polyp stalk.

##### Description of NHMW 2407.

The colony is 3 cm high and wide, the colony stalk 2 cm high (Figure 50B).

Polyps up to about 0.6 mm wide and high (Figure 52A). Supporting bundle projecting up to 0.7 mm, composed of 2–4 spindles (Figure 52A). These spindles are up to 3 mm long, with spines and projecting smooth tip (Figure 52D). Sclerites in polyp are irregularly distributed. Abaxial side of the polyp with curved spindles with spines or simple tubercles, up to 0.6 mm long, several with one smooth end (Figure 52B). Laterally less tuberculated spindles are present, up to 0.2 mm long (Figure 52B). Adaxially and in the tentacles flattened rodlets and ovals are present, up to 0.1 mm long (Figure 52C). The adaxial side of the polyp stalk has small rodlets, up to 0.05 mm long (Figure 52E). The amount of these rodlets varies per polyp, sometimes only a few are present (Figure 52A), others have the whole polyp stalk closely packed with them.

**Surface layer top of stalk.** Spindles with simple tubercles, up to 2.5 mm long, some slightly unilaterally spinose.

**Surface layer base of stalk.** Spindles and unilaterally spinose spindles with simple tubercles, shorter than in the top of the stalk, up to 1.5 mm long. Furthermore small rodlets, several unilateral spinose; smaller branched spindles, radiates and derivatives of these (Figure 53A).

**Interior base of stalk.** The larger interior spindles are not very different from the surface ones, only slightly less tuberculate (Figure 52F). They are up to 1.5 mm long. Smaller, branched bodies also occur (Figure 53B).

**Colour.** Colony is white.

##### Distribution.

Red Sea, Indian Ocean.

##### Variability.

Most colonies examined have slender branches and resemble species of *Stereonephthya* (Figure 51A–B); a few are more “*Litophyton*-like” (Figure 51C).

##### Remarks.

After the very short original description of *Nephthya savignyi* by Ehrenberg (1834: 60), Klunzinger (1877: 35) identified a specimen from Koseir (Red Sea), as *Spongodes* (= *Dendronephthya*) *savignyi*. Kükenthal (1905: 529) examined many specimens, 20 all together, including two specimens from the Berlin museum, one of them Ehrenberg's, “originalexemplar”, and one from the Stuttgart museum, Klunzinger's, specimen. Kükenthal mentioned little variability in all specimens examined and he also synonymized Holm's, *Nephthya jaegerskioeldi* and Nephthea jaegerskioeldi var. microspina with Ehrenberg's, *Dendronephthya savignyi*, based on the presence of polyps in bundles. One of the type specimens of *Nephthea jaegerskioeldi* has been re-examined; the colony and sclerites are presented in Figures 50D, 54–55, and I agree with Kükenthal that the species should be synonymized with Litophyton ?savignyi.

During my visit to the Berlin museum I was unable to find Ehrenberg's, specimen, later on Dr. Goetz Reinicke was so kind to provide me with photographs of a specimen that could be that particular one, though with a question mark (Figure 50A). Indeed doubts remain about the status of the Berlin specimen since it is almost 12 cm wide, while Kükenthal mentioned its width to be 8.5 cm.

The specimen described above is from the Vienna Museum (NHMW 2407) (Figures 50B–C, 52–53), and is probably one of the specimens examined by Kükenthal, as it was found at Tor (Red Sea) and Kükenthal (1905: 531) also examined material from that locality.

Sclerites of ZMTAU 26245 (Figure 51C) are presented to show their variation (Figures 56–58).

Although not re-examined I consider *Spongodes albida* Holm, 1894 synonymous with Litophyton ?savignyi. The specimen is only a few cm long but features all the characters of Litophyton ?savignyi, i.e. projecting supporting bundle, many small rodlets in the polyp stalk and large interior spindles.

In the Red Sea Litophyton ?savignyi differs from all other *Litophyton* species in having polyps with a protruding supporting bundle giving the colony a prickly appearance. The polyps also make an acute angle with the stalk as is seen in the genus *Stereonephthya*. It can only be confused with two species of *Stereonephthya*, *Stereonephthya acaulis* Verseveldt, 1973, and *Stereonephthya cundabiluensis* Verseveldt, 1965. The latter always contains coloured sclerites but *Stereonephthya acaulis* can have white colonies with colourless sclerites (Verseveldt 1973: 153). But it differs from *Litophyton savignyi* in lacking oval tentacle sclerites, having differently shaped polyp stalk rodlets (*Stereonephthya*-type; Figs 13a, 14a, 15a in Ofwegen and Groenenberg (2007)), and having much smaller (up to 0.75 mm long), branched, less tuberculate, internal stalk spindles; see Verseveldt (1973: figs 33n, o).

ZMTAU Co 34066, identified by me as *Dendronephthya savignyi*, was used in a molecular study by McFadden et al (2011). In that study it grouped with other *Litophyton* species, i.e. *Litophyton striatum* (Kükenthal, 1903) (identified by me as *Litophyton elatensis* (Verseveldt & Cohen, 1971), *Litophyton acuticonicum* Verseveldt, 1974, and *Litophyton acutifolium* Verseveldt, 1974 (= *Litophyton viridis*) rather than with other *Dendronephthya* species included in the study.

Unfortunately, Utinomi (1954a) designated *Litophyton savignyi* as the type species for *Dendronephthya*, a genus with more than 250 nominal species. Following strict nomenclatural priority would cause widespread confusion within nephtheid taxonomy. To avoid changing of generic combinations and the confusion that it would cause, a case will be submitted to the International Commission on Zoological Nomenclature (ICZN) to preserve the name *Dendronephthya*, in the meanwhile the species will be cited as Litophyton ?savignyi.

The two *Nephthea* species with projecting supporting bundle described by Verseveldt (1968) from Madagascar, *Nephthea aberrans* and *Nephthea tixierae*, I regard synonymous with Litophyton ?savignyi. I consider the reported differences to represent intra-specific variation.

#### Litophyton simulatum

Taxon classificationAnimaliaAlcyonaceaNephtheidae

(Verseveldt, 1970)

Figures 2D , 59 , 60 , 61 , 62 , 63 , 64 , 65 , 66 , 67 , 68 , 69

Nephthya striata (in part) Kükenthal, 1903: 166, pl. 7 fig. 12, pl. 9 fig. 60 (Red Sea).
Nephthea simulata Verseveldt, 1970: 221, figs 7–8, pl. 2 fig. 1 (Et Tur, Gulf of Suez).

##### Material examined.

**RMNH Coel. 6822**, **part of holotype**, Red Sea, Gulf of Suez, Et Tur, 6 July 1969, coll. L. Fishelson; **RMNH Coel. 8921**, Red Sea, Gulf of Suez, Abu Zanima, 12 June 1968, coll. Hebrew Univ.-Smiths. Red Sea project (identified as *Nephthea chabrolii* by Verseveldt); **RMNH Coel. 8945**, Red Sea, Gulf of Aqaba, Marsa abu Zabad, 15 September 1967, coll. Hebrew Univ.-Smiths. Red Sea project (identified as *Nephthea chabrolii* by Verseveldt); **ZMB 6838**, syntype of *Nephthya striata*, Rotes Meer, Klunzinger leg.; **RMNH Coel. 42085**, Indian Ocean, Socotra, Pbal el Keeri, sta. 188, sample 78, subtidal, 9 April 1999, coll. G. Reinicke; **RMNH Coel. 42086**, Indian Ocean, Socotra, Darsa, sta. 245, sample 89, subtidal, 8 April 1999, coll. G. Reinicke; **RMNH Coel. 42092**, Indian Ocean, Chagos Archipelago, 7°0'S, 72°30'E, Peros Banhos, Diamond, 28 February 1996, coll. G.B. Reinicke, no. 3; **RMNH Coel. 42093**, Indian Ocean, Chagos Archipelago, 7°0'S, 72°30'E, Peros Banhos, Ile Vache Marine, lagoon, 6 March 1996, coll. G.B. Reinicke, no. 87; **RMNH Coel. 42094**, Indian Ocean, Chagos Archipelago, 7°0'S, 72°30'E, Salomoms Atoll, Ile Fouquet, 8 March 1996, coll. G.B. Reinicke, no. 112; **RMNH Coel. 42095**, Indian Ocean, Chagos Archipelago, 7°0'S, 72°30'E, Salomons, off Ile de la Passe, 11 March 1996, coll. G.B. Reinicke, no. 184; **RMNH Coel. 42096**, Indian Ocean, Chagos Archipelago, 7°0'S, 72°30'E, Great Chagos Bank, Nelson Island, NE corner, 12 March 1996, coll. G.B. Reinicke, no. 194; **RMNH Coel. 42097**, Indian Ocean, Chagos Archipelago, 7°0'S, 72°30'E, Great Chagos Bank, Middle Brother, lagoon, 13 March 1996, coll. G.B. Reinicke, no. 226; **ZMTAU Co 25839 1781**, Red Sea, South tip Sinai, Tiran Strait, depth 0–35 m, 13 July 1986, coll. Y. Benayahu; **ZMTAU Co 25841**, Red Sea, South tip of Sinai, Ras Muhamad, depth 0–25 m, 14 July 1986, coll. Y. Benayahu; **ZMTAU Co 25844 1861**, Red Sea, South tip Sinai, Tiran St., depth 30 m, 15 July 1986, coll. Y. Benayahu; **ZMTAU Co 25856 (2015**), Red Sea, Gulf of Suez, Shag Rock, depth 3–24 m, 14 July 1987, coll. Y. Benayahu; **ZMTAU Co 25874**, Red Sea, South tip Sinai, South to Shag Mahmud, depth 10 m, 10 July 1986, coll. Y. Benayahu; **ZMTAU Co 25970**, Red Sea, Gulf of Suez, Jubal Is., depth 30 m, 22 March 1988, coll. Y. Benayahu; **ZMTAU Co 26201**, Red Sea, Gulf of Aqaba, Nageb Shahin, depth 18 m, 5 November 1981, coll. Y. Benayahu; **ZMTAU Co 26211**, Red Sea, Gulf of Aqaba, Shurat el Manqata, 9 November 1981, coll. Y. Benayahu; **ZMTAU Co 26213**, Red Sea, Gulf of Aqaba, Shurat el Manqata, 9 November 1981, coll. Y. Benayahu; **ZMTAU Co 26224**, Red Sea, Gulf of Suez A-Tur, 6 July 1969, coll. Y. Benayahu; **ZMTAU Co 26227**, Red Sea, Gulf of Suez, Ras Rareb, 24 August 1971, coll. D. Popper; **ZMTAU Co 26230**, Red Sea, Gulf of Suez, Ras Tanaka, 25 September 1974, coll. Y. Benayahu; **ZMTAU Co 26237**, Red Sea, Gulf of Aqaba Muqeibla, depth 3 m, 17 April 1979, coll. Y. Benayahu; **ZMTAU Co 26240**, 3 colonies, all 3 cut in half lengthwise, Red Sea, Gulf of Aqaba W. Magrash km 207, 17 April 1979, coll. Y. Benayahu; **ZMTAU Co 26244**, Red Sea, Gulf of Aqaba Wadi Magrash km 207, 20 July 1974, coll. Y. Benayahu (in bottle with neotype of *Litophyton chabrolii*); **ZMTAU Co 34030**, Indian Ocean, Chagos Archipelago, Ile Fouquet, 5°28.870'S, 71°48.762'E, 12 February 2006, coll. M. Schleyer; **ZMTAU Co 34040**, Indian Ocean, Chagos Archipelago, Middle Brother, 6°8.929'S, 71°31.630'E, 7 February 2006, coll. M. Schleyer; **ZMTAU Co 34042**, Indian Ocean, Chagos Archipelago, Ile de la Passe, 5°17.943'S, 72°15.449'E, 11 March 2006, coll. M. Schleyer; **ZMTAU Co 34043**, Indian Ocean, Chagos Archipelago, Ile Anglaise, 5°20.439'S, 72°12.809'E, 17 February 2006, coll. M. Schleyer; **ZMTAU Co 34046**, Indian Ocean, Chagos Archipelago, Ile Anglaise, 5°20.439'S, 72°12.809'E, 17 February 2006, coll. M. Schleyer; **NTM C02254**, C. of Eilat, Muqebla, depth 3 m; 8 August 1975; coll. Y. Benayahu.

##### Diagnosis.

*Litophyton* with internal spindles of the base of the stalk short, mostly up to 0.5 mm long, up to 0.25 mm wide. Spindles with blunt ends and branched spindles present.

##### Description

**(after Verseveldt 1970)**. The specimen RMNH Coel. 6822 is the left part of the holotype as depicted by Verseveldt (1970: pl. 2 fig. 1).

The polyps are up to about 0.65 mm wide and 0.8 mm high. Supporting bundle not projecting, composed of spindles with simple tubercles, outer side and distal end with more tubercles. Length of these spindles is up to 1.1 mm. Polyp body sclerites irregularly arranged, the smallest are present adaxially; abaxially they merge into the smaller spindles of the supporting bundle and have larger tubercles on the outer side. The tentacle sclerites resemble the smallest adaxial polyp sclerites.

**Surface layer top of stalk.** Spindles, radiates, and derivatives of these, merging into unilaterally spinose spindles; all sclerites with simple tubercles. The spindles are up to 0.25 mm long.

**Surface layer base of stalk.** Sclerites similar to those of the top of the stalk but longer, up to 0.4 mm long; the unilaterally spinose sclerites having longer spines.

**Interior base of stalk.** Spindles, up to 1.0 mm long, with simple sparse tubercles. Several spindles have one or more side branches, the smaller spindles are more often branched than the larger ones. A few spindles have blunt ends.

**Colour.** The colony is grey.

##### Distribution.

Red Sea, Socotra, Chagos Archipelago.

##### Remarks.

The microscope slides of transparent polyps made by Verseveldt (1970) show smooth rodlets in the adaxial polyp body, his drawing of a polyp (Figure 7I) also shows a few, and they are even mentioned in his description. However, they represent the end views of polyp sclerites as smooth rodlets are not present in the slides of polyp sclerites.

Verseveldt (1970) compared the species with *Litophyton laevis* (Kükenthal, 1913) and concluded the tuberculation of the sclerites, being much stronger in *Litophyton simulatum*, was the main character to distinguish between them. Also the interior sclerites differ, in *Litophyton laevis* they are slender, and only up to 0.5 mm long, in *Litophyton simulatum* they are twice as wide and up to 1.0 mm long.

The species also resembles *Litophyton striatum* as that species has also branched interior spindles in the base of the stalk. However, the sclerites of the interior of the base of the stalk of *Litophyton striatum* always include spindles with many small side branches or extra tall tubercles, while those of *Litophyton simulatum* have just one or a few.

RMNH Coel. 8921 and 8945, both identified by Verseveldt as *Nephthea chabrolii*, proved to be *Litophyton simulatum*.

ZMB 6838, a syntype of *Litophyton striatum* (Kükenthal, 1903), shows sclerites characteristic of *Litophyton simulatum* (Figures 59A, 60–61).

For showing variation sclerites of ZMTAU Co 25874 (Figures 59B, 62–65) and ZMTAU Co 26201 (Figures 59C, 66–69) are presented.

#### Litophyton striatum

Taxon classificationAnimaliaAlcyonaceaNephtheidae

(Kükenthal, 1903)

Figures 2C , 70 , 71 , 72 , 73 , 74 , 75 , 76 , 77 , 78 , 79 , 80 , 81 , 82

Nephthya striata (in part) Kükenthal, 1903: 166, pl. 7 fig. 12, pl. 9 fig. 60 (Red Sea); 1913: 20 (Red Sea).
Litophyton striatum Not Nephthea striata; Thomson and Dean 1931: 89 (Indonesia); Verseveldt 1966: 16, figs 8-10, pl. 2 fig. 2 (Morotai, Moluccas, Indonesia; material compared with that of Thomson and Dean 1931); 1974b: 2 (Fara’un I., Gulf of Aqaba, Red Sea; listed only = Litophyton acuticonicum); Tixier-Durivault 1966: 282, figs 264–266 (Madagascar); 1970b: 299 (New Caledonia); 1972: 26 (Madagascar); Imahara 1991: 73, fig. 12, pl. IIc (Kerama Islands, Ryukyu Is., Japan; 13m); Imahara 1996: 25 (listed).
Nephthea galbuloides Verseveldt, 1973: 144, figs 26–28 (Andraikarekabé, Nosy Komba, near Nosy Bé, Madagascar; Pointe Ambarionaomby, Nosy Komba, near Nosy Bé, Madagascar; Tany Kely, near Nosy Bé, Madagascar).
Litophyton striatum Not Nephthea galbuloides; Verseveldt 1977b: 303 (Ambon, Indonesia); Ofwegen 1996: 209 (Papua New Guinea).
Nephthea elatensis Verseveldt & Cohen, 1971: 53, fig. 1 (Red Sea); Verseveldt 1970: 210 (Red Sea, listed only); 1974b: 2 (Red Sea; listed only); McFadden et al. 2011: 25.

##### Material examined.

**SMF 1279**, syntype *Nephthya striata*, Rotes Meer; Rüppell leg. 1832; **ZMB 6837**, syntype *Nephthya striata*, Rotes Meer, Rüppell leg. 1832; **ZMB 6838**, syntype *Nephthya striata*; Rotes Meer, Klunzinger leg. (= *Litophyton simulatum*); **RMNH Coel. 6866**, *Nephthea elatensis*, holotype, Red Sea, Gulf of Aqaba, opposite Solar Lake (SWS), depth 4 m, August 1969, coll. J. Cohen; **RMNH Coel. 8048**, holotype *Nephthea galbuloides*, Andraikarekabe, Madagascar, depth 3 m; **RMNH Coel. 8049**, paratypes *Nephthea galbuloides* Pointe Ambarionaomby, Madagascar, depth 1 m; **RMNH Coel. 6820**, Red Sea, Dahab, Gulf of Aqaba, 10 October 1968, (identified by Verseveldt as *Nephthea elatensis*); **RMNH Coel. 12316**, Red Sea, Gulf of Aqaba, Sharm el Sheikh, depth 6 m, 7 September 1976, coll. Y. Benayahu; **RMNH Coel. 42088**, Indian Ocean, Socotra, sta. 79, subtidal, 10 April 1999, coll. G. Reinicke; **RMNH Coel. 42089**, Indian Ocean, Socotra, sta. 268, sample 87, subtidal, 15 April, 1999, coll. G. Reinicke; **RMNH Coel. 42090**, Indian Ocean, Socotra, Samha, NE coast, sta. 334, sample 88, subtidal, 16 April 199, coll. G. Reinicke; **RMNH Coel. 42091**, Indian Ocean, Socotra, Kal Farun, sta. 209, sample 81, subtidal, 11 April 1999, coll. G. Reinicke; **RMNH Coel. 42098**, Indian Ocean, Chagos Archipelago,(7°0'S, 72°30'E), Peros Banhos, Diamond, 28 February 1996, coll. G.B. Reinicke, no. 2; **ZMTAU NS 1726**, Red Sea, Gulf of Aqaba, Dahab, 13 September 1967, coll. Fishelson; **ZMTAU Co 25686 1966**, Red Sea, South tip Sinai, Shab Mahmud, 12 July 1987, coll. Y. Benayahu; **ZMTAU Co 25826 1492**, Red Sea, South tip Sinai, Shab Mahmud, depth 20–30 m, coll. Y. Benayahu, 9 July 1986; **ZMTAU Co 25828 1520**, Red Sea, South tip Sinai, Shab Mahmud, depth 20–30 m, 9 July 1986, coll. Y. Benayahu; **ZMTAU Co 25832 1600**, Red Sea, Gulf of Suez, Shag Rock, 0–20 m, 10 July 1986, coll. Y. Benayahu; **ZMTAU Co 25838 1753**, Red Sea, South tip Sinai, Tiran St., depth 0–35 m, 13 July 1986, coll. Y. Benayahu; **ZMTAU Co 25851** 1902, Red Sea, South tip Sinai, Shab Mahmud, depth 0–20 m, 12 July 1987, coll. Y. Benayahu; **ZMTAU Co 26192**, Red Sea, Straits of Tiran Ras Nazrani, 14 March 1981, coll. Y. Benayahu; **ZMTAU Co 26194**, Red Sea, Tiran Is. lagoon, depth 4 m, 15 March 1981, coll. Y. Benayahu; **ZMTAU Co 26195**, Red Sea, Tiran Is. lagoon, depth 4 m, 15 March 1981, coll. Y. Benayahu; **ZMTAU Co 26200**, Red Sea, Gulf of Aqaba, Dahab southern Oasis, depth 10 m, 4 November 1981, coll. Y. Benayahu; **ZMTAU Co 26203**, Red Sea, South tip Sinai, Sharm a Sheikh, Gan Eden, 6 November 1981, coll. Y. Benayahu; **ZMTAU Co 26207**, Red Sea, Gulf of Aqaba, El Goz (N of Tiran strait), depth 2–5 m, 8 November 1981, coll. Y. Benayahu; **ZMTAU Co 26216**, Red Sea, South tip Sinai, Sharm a Sheikh, depth 20 m, 30 November 1981, coll. Y. Benayahu; **ZMTAU CO 34112**–**34113**, 2 specimens, Red Sea, Gulf of Aqaba, Elat, 29°30.14'N, 34°55.075'E, depth 10.7–12.2 m, coll. Y Benayahu, 24 July 2007 (identified by Ofwegen as *Nephthea elatensis*); **ZMTAU Co 30062**, 3 specimens, Eritrea, Entedeber Is., 15°43.020'N, 39°53.465'E, 1 May 1997, depth 6.5 m, coll. Y. Benayahu; **ZMTAU Co 34034**, Indian Ocean, Chagos Archipelago, Ile Fouquet, 5°28.870'S, 71°48.762'E, 12 February 2006, coll. M. Schleyer; **ZMTAU Co 34036**, Indian Ocean, Chagos Archipelago, Middle Brother, 6°8.929'S, 71°31.630'E, 7 February 2006, coll. M. Schleyer.

##### Removed from the species.

**RMNH Coel. 2238**, Indonesia, N Moluccas, Morotai, Snellius expedition, 3–10 June 1930; **RMNH Coel. 8939**, identified by Verseveldt as *Nephthea striata*, Red Sea, Gulf of Aqaba, Fara ‘un Island, 27 June 1967, Hebrew Univ.-Smiths. Red Sea project, 2/SLR 45 (= *Litophyton acuticonicum*); **RMNH Coel. 12317**, identified as *Nephthea striata* by Verseveldt, Red Sea, Gulf of Aqaba, Sharm el Sheikh, depth 30 m, 7 September 1976, coll. Y Benayahu (= *Litophyton curvum* sp. n.).

##### Diagnosis.

*Litophyton* with the large internal spindles of the base of the stalk at least 1.0 mm long and heavily branched.

##### Re-description of the lectotype, SMF 1279.

The stiff lectotype is 7 cm long and 5 cm wide (Figure 70A); end lobes rounded. Colony stalk very short, up to 1 cm long.

The polyps are up to 0.80 in height and up to 0.90 in width (Figure 71A). Supporting bundle mostly not projecting, sometimes one spindle projecting for 0.10 mm; it is composed of up to about 10 spindles; these spindles are up to 1.15 mm long and up to 0.13 mm wide; with simple tubercles and spines (Figure 71B). Distal end of projecting spindles with higher spines. Polyp body sclerites irregularly arranged. On the abaxial and lateral sides the spindles are up to 0.35 mm long; with thorns on the outer side (Figure 71C). Adaxially only some small, spiny rodlets are present, about 0.1 mm long. On the adaxial side of the polyp stalk, just below the polyp body, similar rodlets are present, placed transversely; tentacles with nearly smooth rodlets, 0.03–0.08 mm long (Figure 71D).

**Surface layer top of stalk.** Radiates and derivatives of these, spindles and unilaterally spinose spindles, which are up to 0.6 mm long. Several sclerites with some side-branches (Figure 71E).

**Surface layer base of stalk.** Sclerites similar to those of the top of the stalk but with longer spines (Figure 72A). Few unilaterally spinose spindles present.

**Interior base of stalk.** Spindles with widely placed simple spines and tubercles; the spindles often have side-branches (Figure 72B–C). Length of these spindles up to 1.6 mm.

**Colour.** Colony cream.

##### Distribution.

Red Sea, Socotra, Chagos Archipelago, Madagascar.

##### Remarks.

In the catalogue of the ZMB the numbers 6833–6838 are mentioned as material of *Nephthea striata*, 6834 and 6838 as types. I could only find two specimens (numbers 6837 and 6838). In the SMF one specimen is present (SMF 1279), that clearly is the same specimen as the one described and depicted by Kükenthal (1903: 166, pl. 7 fig. 12). Kükenthal, in his description of the species, mentioned two other specimens (both about 3 cm high and wide) from the Red Sea. According to Kükenthal (1903) these two specimens were deposited in the Breslau Museum. Nowadays no type material of this species is present in Breslau. Most probably ZMB 6838 (Figure 59A) is one of the two Breslau specimens. The sclerites of ZMB 6838 (Figures 60–61) show it to be a specimen belonging to *Litophyton simulatum*. ZMB 6837 lacks the base of the colony and therefore some doubts about its identity remains but probably it represents the same species SMF 1279, which is here designated as the lectotype of *Litophyton striatum*.

The lectotype SMF 1279 has some supporting bundle spindles with a somewhat leafy projecting end (not depicted); ZMTAU 26194 and ZMTAU 26195 have some with a smooth spine (not depicted).

ZMTAU Co 25851 (Figures 70B, 73–76), ZMTAU Co 26216 and ZMTAU Co 26203 (Figures 70C, 77–79) have been used to produce SEM images of the sclerites. Noteworthy is the difference in internal base stalk spindles (Figures 76, 78). ZMTAU Co 26203 shows an unusual amount of unilaterally spinose sclerites in the surface of the base of the stalk, with densely placed spines which are not like those in SMF 1279 (Figure 79).

The type material of *Nephthea galbuloides* has been re-examined (Figures 80–82) and proved to be *Litophyton striatum*. This specimen also shows very densely arranged spines on the unilaterally developed forms.

ZMTAU CO 34112–34113, identified by myself as *Nephthea elatensis*, have been used by McFadden et al. (2011) for their molecular study.

#### Litophyton viridis

Taxon classificationAnimaliaAlcyonaceaNephtheidae

(May, 1898)

Figures 1F , 83 , 84 , 85 , 86 , 87 , 88 , 89 , 90 , 91 , 92 , 93 , 94 , 95 , 96 , 97

Ammothea viridis May, 1898: 33 (Muemba Island; East Africa); 1899: 139, pl. 2 fig. 23, pl. 5 figs 11a–b.
Litophytum viridis ; Kükenthal 1903: 115 (May's, type + Baui Island; East Africa).
Litophyton viridis ; Ofwegen and Benayahu 1992: 140 (Tanzania).
Litophyton viridis Not Litophytum viride; Roule 1908: 172 (Ambon); Bayer et al. 1983: pl. 17 fig. 121 (= Litophyton arboreum Forskål, 1775).
Litophyton viridis Not Litophytum viridis; Thomson and Dean 1931: 70 (Indonesia).
Litophyton viridis Not Litophyton viridis; Tursch 1976: 2 (Indonesia, Moluccas, Leti island); Bortolotto et al. 1977: 159 (Indonesia, Sunda islands).
Ammothea stuhlmanni May, 1898: 34 (East-Africa); 1899: 140, pl. 3 fig. 25.
Litophytum stuhlmanni ; Kükenthal 1903: 116 (re-examination of May's, types).
Litophyton viridis Not Litophytum stuhlmanni; Thomson and Dean 1931: 70, pl. 23 fig. 9 (Indonesia).
Litophyton viridis Not Litophyton stuhlmanni; Tixier-Durivault 1970a: 223 (Vietnam).
Ammothea sanderi May, 1899: 141, pl. 3 fig. 26, pl. 5 fig. 12 (Zanzibar).
Litophytum sanderi ; Kükenthal 1903: 119 (re-examination of May's, type).
Litophyton sanderi ; Verseveldt and Benayahu 1983: 4 (Eilat, Gulf of Aqaba, 40-45 m, leg. Ch. Lewinsohn; listed only).
Litophytum crosslandi Thomson & McQueen, 1908: 56 (Red Sea, Coral reef of Khor Delaweb, 3-4 feet).
Litophytum acutifolium Kükenthal, 1913: 12, fig. 1, pl. 1 fig. 1 (Egyptian Red Sea coast, Berenice); McFadden et al. 2011: 25.
Litophyton viridis Not Litophyton acutifolium; Verseveldt 1974b: 25, figs 19-18 (Gulf of Aqaba, Red Sea = Litophyton acuticonicum); 1977b: 303 (Gunung Api, Banda Is., Indonesia; 1978: 50 (Palau).

##### Material examined.

**ZMH C2396**, syntype *Litophytum viridis*, Stuhlmann Id. 1889; May 1898; Kükenthal, 1902; Sansibar, Insel Baui; **ZMH C2397**, syntype *Litophytum viridis*, Stuhlmann Id. 1889; May 1898; Kükenthal, 1902; Sansibar, Insel Muemba; **ZMB 6709**, **6710**, syntypes *Litophytum viridis* (May), Sansibar, Stuhlmann leg., Kükth det. 1902, Breslau, not registered as type material (see remarks); **ZMH C2391**, syntype *Ammothea stuhlmanni*; **ZMH C2390**, holotype *Ammothea sanderi*; **BM 1933.3.13.193**, holotype *Litophytum crosslandi*; **NHMW C2347**, part of the holotype of *Litophytum acutifolium*; **ZMB 6682**, part of the holotype of *Litophytum acutifolium*; **ZMB 6683**, part of the holotype of *Litophytum acutifolium*; **RMNH 18916**, identified as *Litophyton viridis* by Ofwegen and Benayahu, 1992, Tanzania, off Dar es Salaam, Funguyasini Island, leeward slope, coll. J.N. Nyanda; **ZMTAU NS 1711**, Red Sea, Gulf of Aqaba, Dahab, coll. L. Fishelson, 13 September 1967; **ZMTAU Co 25968**, Red Sea, South tip Sinai Ras um Sud Temple, 26 March 1988, coll. Y. Benayahu; **ZMTAU Co 26107 3131**, Red Sea, Gulf of Suez, Tawilla Is., depth 6–10 m, 24 September 1989, coll. Y. Benayahu; **ZMTAU Co 26193**, Red Sea, Tiran Isl., depth 4 m, coll. Y. Benayahu, 15 March 1981; **ZMTAU Co 26196**, Red Sea, Tiran Island, depth 4 m, coll. Y. Benayahu, 15 March 1981; **ZMTAU Co 26197**, Red Sea, Gulf of Eilat “Fjord”, depth 2–3 m, coll. Y. Benayahu, 16 April 1979; **ZMTAU Co 26199**, Red Sea, Gulf of Aqaba, Dahab southern oasis, depth 4 m, 4 November 1981, coll. Y. Benayahu; **ZMTAU Co 26204**, Red Sea, Strait of Tiran, South of Ras Nazrani, 7 November 1981, coll. Y. Benayahu; **ZMTAU Co 26222**, Red Sea, Marsa Murach, south of Eilat, 23 July 1968, coll. L. Fishelson; **ZMTAU Co 26241**, Red Sea, Tiran Is. Favel bay lagoon, depth 1–2 m, 22 September 1981, coll. Kerman; **ZMTAU Co 26242**, Red Sea, Gulf of Aqaba, south Muqeibla, coll. Y. Benayahu, 30 March 1976; **ZMTAU Co 34114** Red Sea, Gulf of Aqaba, Elat, 29°30.14'N, 34°55.075'E, depth 10.7–12.2 m, 24 July 2007, coll. Y. Benayahu; ?**ZMTAU Co 26243**, Red Sea, Gulf of Suez Ras Gahra, 27 September 1974, coll. Y. Benayahu; **ZMTAU Co 28591** (E220), Eritrea, Dahlak Archipelago, Madut, depth 3 m, 16 October 1993, coll. Y. Benayahu; ?**ZMTAU 32941**, Eritrea, Dahlak Archipelago, between Nocra Is. and Dahlak Is., southern entrance to the channel, 15°41.36'N, 39°56.08'E, depth 0–5 m, 14 February 2005, coll. Y. Benayahu; ?**ZMTAU Co 33091**, Red Sea, Gulf of Aqaba, Elat, marine lab, IUI reef, May 2005, coll. Y. Benayahu.

##### Removed from the species.

**RMNH 11835**, identified by Verseveldt as *Litophyton acutifolium*, Banda Isl., depth 10 m; **RMNH 12836**, identified by Verseveldt as *Litophyton acutifolium*, Palau Isl, depth 15 ft; **RMNH 12839**, identified by Verseveldt as *Litophyton acutifolium*, Palau Isl.; **RMNH Coel. 11940**, identified by Verseveldt as *Litophyton stuhlmanni*, Indonesia, Moluccas, S of Obi, Poelau Gomumu, 1°50'S, 127°30'45"E, depth 3 m, 30 May 1975, coll. A.G. Humes, 1990 R/V “Alpha Helix”.

##### Diagnosis.

Colonies flabby, end lobes finger-like. Polyps with irregularly arranged, smooth rodlets adaxially and spiny rodlets abaxially; these rodlets are up to 0.1 mm long. Sometimes a few spindles are also present in the polyp stalk; sometimes the polyps are unarmed. Surface base of stalk with radiates, derivatives of these, and unilateral spinose spindles, the latter up to 0.5 mm long; many with side branches. Interior base of stalk with spindles up to 1 mm long; they can have side branches.

##### Re-description of syntype ZMH C2396.

Colony flabby, 10.5 cm long and 7 cm wide (Figure 83A). Catkins finger-like.

**Polyps and branches.** Without sclerites (Figure 87A).

**Surface layer top of stalk.** Capstans, spindles and unilateral spinose spindles; all with closely set tubercles; length up to 0.15 mm (Figure 87B).

**Surface layer base of stalk.** Capstans, spindles and branched spindles; the spindles up to 0.85 mm long (Figure 87C).

**Interior base of stalk.** Spindles with widely placed simple tubercles (Figure 87D); the spindles can be branched or have side branches.

**Colour of colony.** Cream.

##### Distribution.

Red Sea, East Africa.

##### Remarks.

Syntype ZMH C2397 (Figures 83B, 88) shows more cone-shaped catkins, in all other characters it agrees with ZMH C2396.

May (1898) mentioned three specimens. Only two are present in the ZMH, ZMB 6709 and ZMB 6710 probably represent the missing ZMH specimen. Kükenthal (1903) re-examined one of May's, specimens and a specimen collected by Voeltzkow from the Island Baui. As the labels of the ZMB material mention Stuhlmann leg., the same as May's, material I assume I am here dealing with May's, material. ZMB 6709 and ZMB 6710 are not registered as type material and therefore they were not photographed, although a fragment of the top of ZMB 6710 was re-examined.

The difference between *Litophyton viridis*, *Litophyton acutifolium*, *Litophyton crosslandi*, *Litophyton sanderi*, and *Litophyton stuhlmanni* is only based on the polyps, those of *Litophyton viridis* having no sclerites at all, while the other four species have few sclerites in the polyps. I regard the polyps without sclerites of *Litophyton viridis* an extreme case of a species with a few sclerites in the polyps and synonymize *Litophyton acutifolium*, *Litophyton crosslandi*, *Litophyton sanderi*, and *Litophyton stuhlmanni* with *Litophyton viridis*.

May (1898) mentioned two specimens of *Litophyton stuhlmanni*, the specimen examined, ZMH C2391 (Figures 84, 89) is different from the one depicted by May (1899: pl. 3 fig. 25). May (1899) and Kükenthal (1903), who re-examined May's, material, described the polyps as being devoid of sclerites. I assume that both missed the polyp sclerites hidden in detritus inside the polyps.

Apart from being much smaller (Figure 83C), *Litophyton sanderi* has much in common with *Litophyton stuhlmanni*. Kükenthal (1903) already recognized this close resemblance but kept the species separate because he could not find any sclerites in the polyps of *Litophyton stuhlmanni*. For comparison the sclerites of the holotype of *Litophyton sanderi* are depicted (Figures 90–91). The small sclerite differences with *Litophyton stuhlmanni* I consider to be intraspecific variation.

The colony fragment of *Litophyton crosslandi* present in the Natural History Museum (BM 1933.3.13.193) is only part of the colony originally described. The total length of the fragment is 5.3 cm (Figure 83D; notes of Verseveldt) while Thomson and McQueen mentioned branches up to 13 cm long. The two microscope slides examined only show sclerites found in the top of their colony (Figure 92). Some polyps of the holotype of *Litophytum crosslandi* also show the “ring of slerites in the tentacle basis” mentioned by Kükenthal (1913) for his *Litophyton acutifolium*.

The holotype of *Litophyton acutifolium* (Kükenthal, 1913: pl. 1 fig. 1) was cut into pieces, and these are now stored as ZMB 6682, ZMB 6683 (Figure 85B), and NHMW C2347 (Figure 85A). For comparison the sclerites of ZMB 6683 and some of those of NHMW C2347 are depicted (Figures 93–95). The small sclerite differences noted are considered to be intraspecific variation.

ZMTAU Co 32941 and 33091 are only fragments of the top of colonies, the flabby nature of the fragments together with the sclerites matching those of *Litophyton viridis* made me identify them as this species.

ZMTAU Co 34114 (previously identified as *Litophyton acutifolium* by me) has been used by McFadden et al. (2011) for their molecular study.

ZMTAU 26193 (Figure 86) is used for presenting SEM images of sclerites (Figures 96–97).

*Litophyton maldivensis* and *Litophyton acuticonicum* both also have polyps with limited amount of sclerites. *Litophyton acuticonicum* differs from *Litophyton viridis* in having much larger interior stalk sclerites (up to 2 mm long). *Litophyton maldivensis* has overall much smaller interior stalk sclerites which mostly have blunt ends. *Litophyton striatum* has similar looking sclerites in the interior of the base of the stalk as ZMTAU 26193 but in that species the polyps are much stronger armed.

### Figures

**Figure 3.**
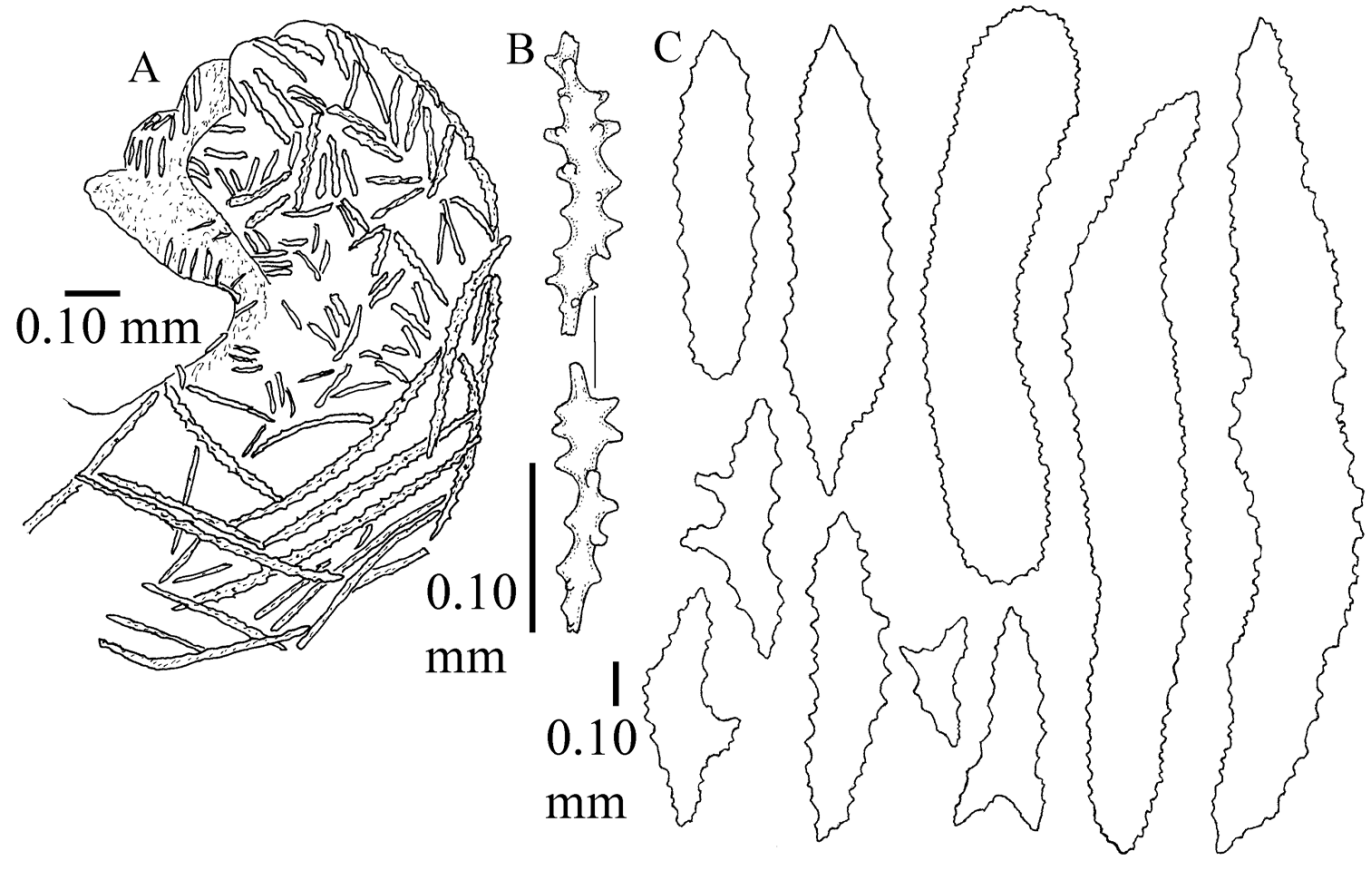
*Litophyton acuticonicum* (Verseveldt, 1974), holotype RMNH Coel. 8920. **A** lateral view of polyp armature **B** polyp body spindles **C** spindles interior base of stalk, outlines only.

**Figure 4.**
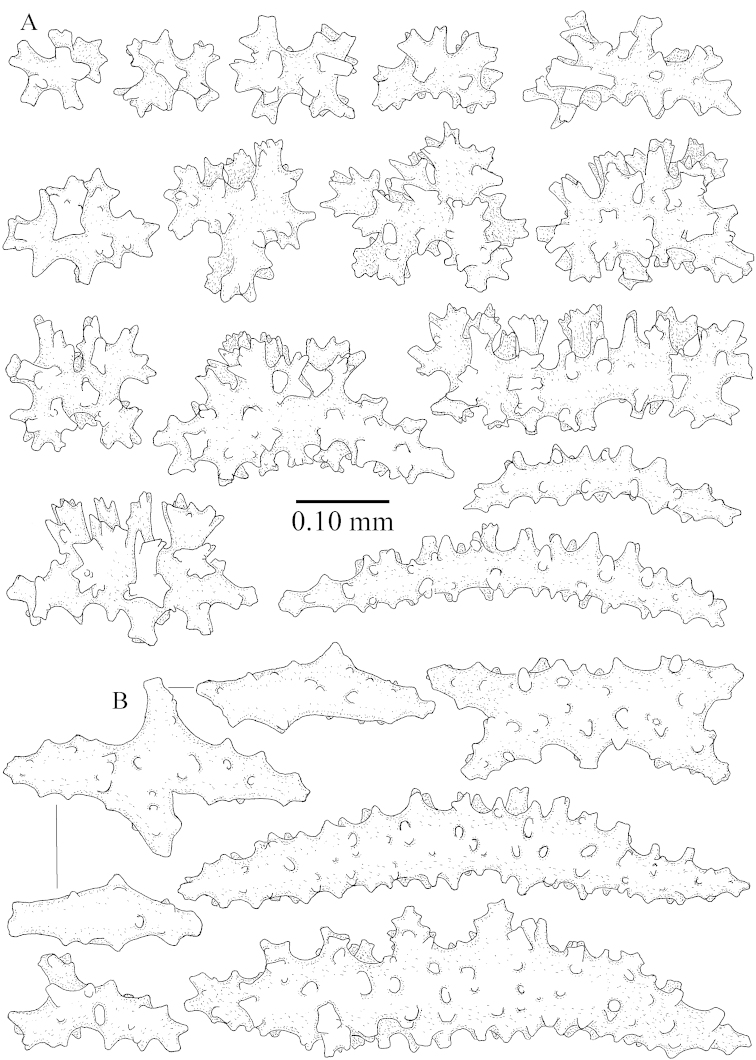
*Litophyton acuticonicum* (Verseveldt, 1974), holotype RMNH Coel. 8920. **A** sclerites surface layer base of stalk **B** spindles of interior base of stalk.

**Figure 5.**
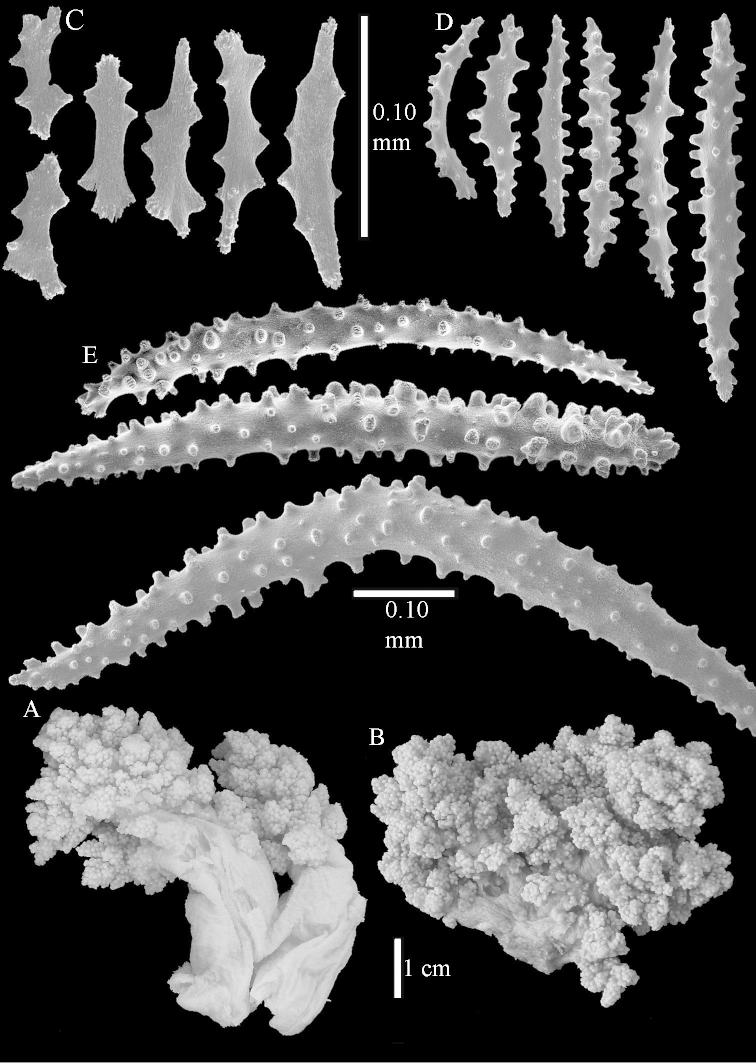
*Litophyton acuticonicum* (Verseveldt, 1974). **A** ZMTAU Co 25867 **B** ZMTAU Co 26239 **C–E** ZMTAU Co 25867 **C** tentacle rodlets **D** polyp body spindles **E** spindles of supporting bundle. Scale at **E** also applies to **D**.

**Figure 6.**
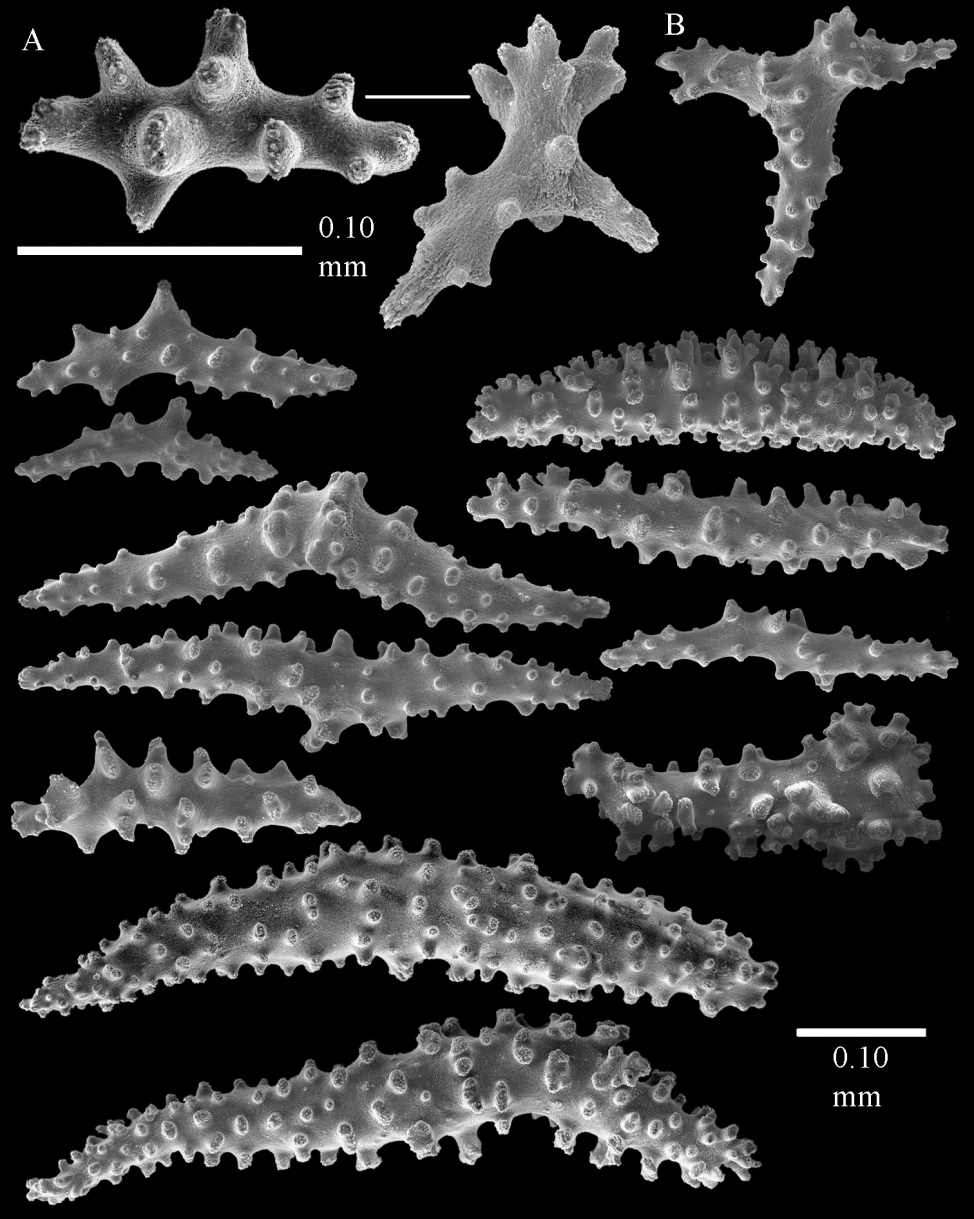
*Litophyton acuticonicum* (Verseveldt, 1974), ZMTAU Co 25867. **A–B** sclerites of surface layer top of stalk.

**Figure 7.**
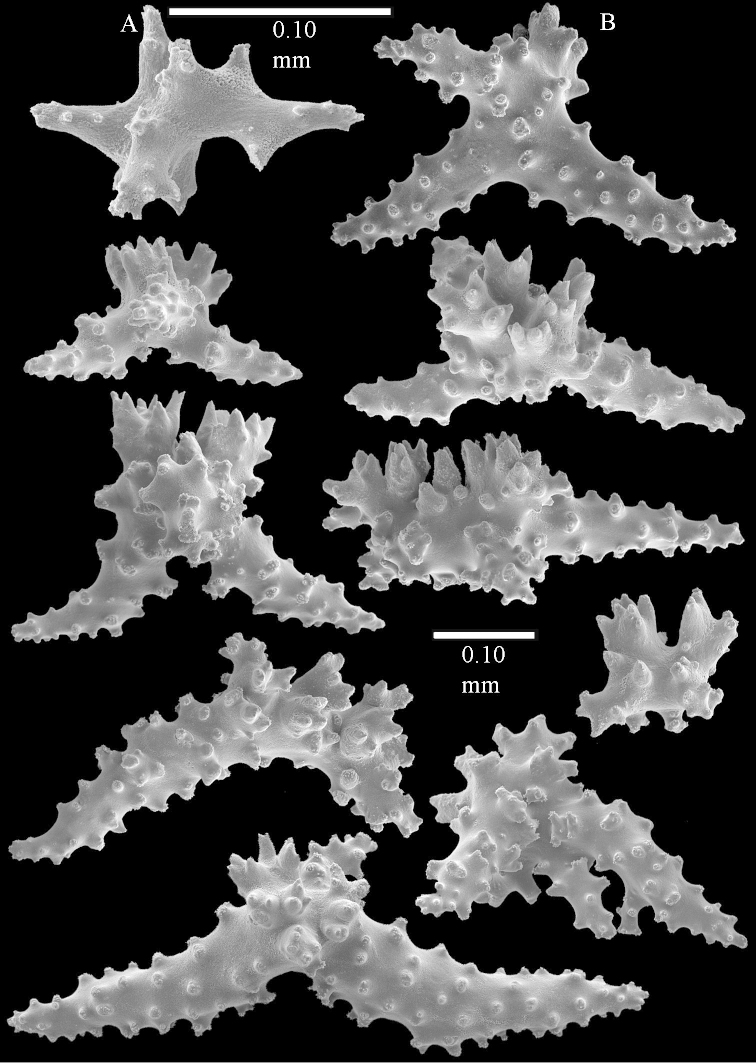
*Litophyton acuticonicum* (Verseveldt, 1974), ZMTAU Co 25867. **A–B** sclerites of surface layer base of stalk.

**Figure 8.**
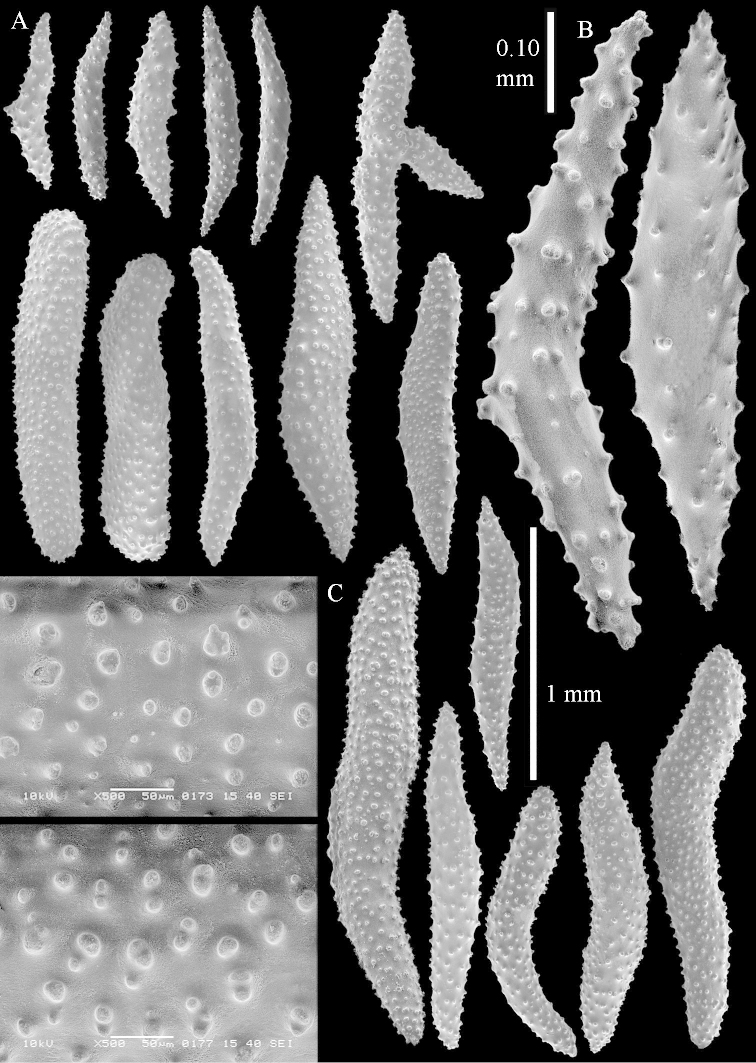
*Litophyton acuticonicum* (Verseveldt, 1974), ZMTAU Co 25867. **A–B** sclerites of interior base of stalk **C** tubercles on spindle.

**Figure 9.**
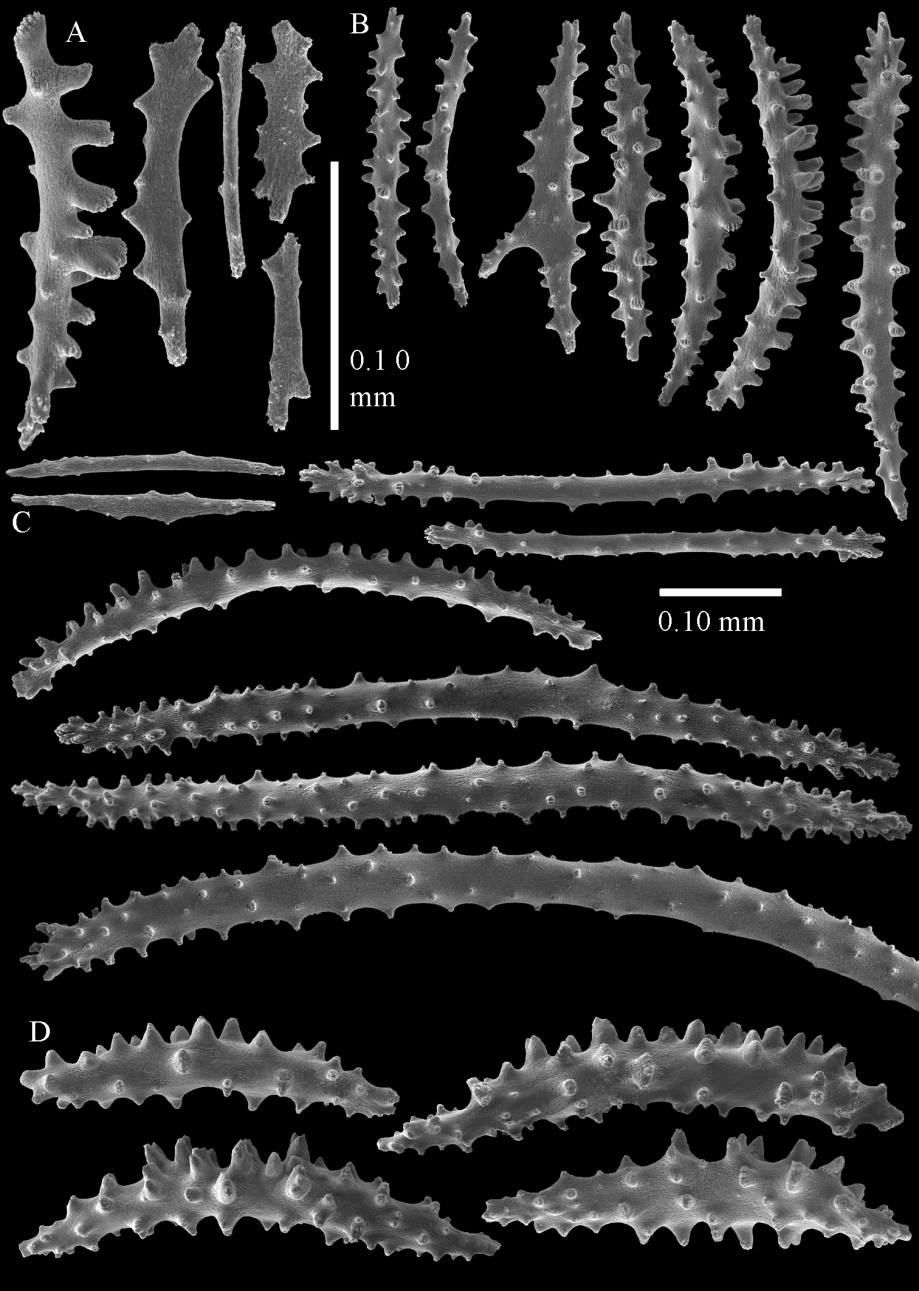
*Litophyton acuticonicum* (Verseveldt, 1974), ZMTAU Co 26239. **A** tentacular and small polyp body sclerites **B** polyp body spindles **C** spindles of supporting bundle **D** sclerites of surface layer top of stalk. Scale at **C** also applies to **B, D**.

**Figure 10.**
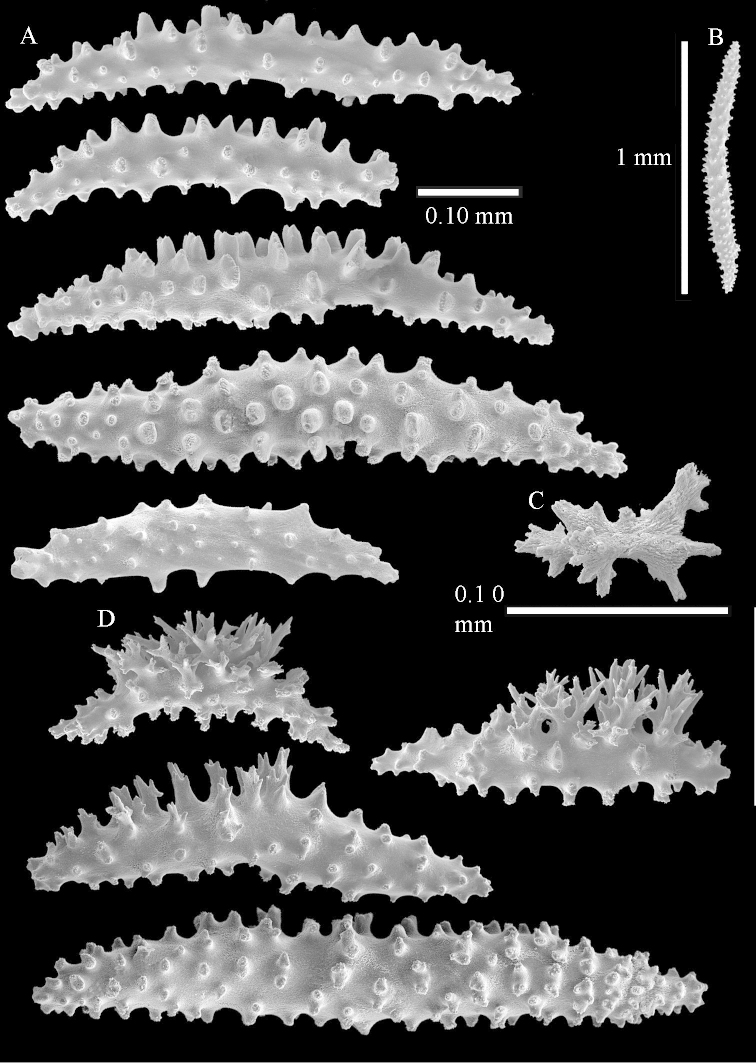
*Litophyton acuticonicum* (Verseveldt, 1974), ZMTAU Co 26239. **A–B** sclerites of surface layer top of stalk **C–D** sclerites of surface layer base of stalk. Scale at **A** also applies to **D**.

**Figure 11.**
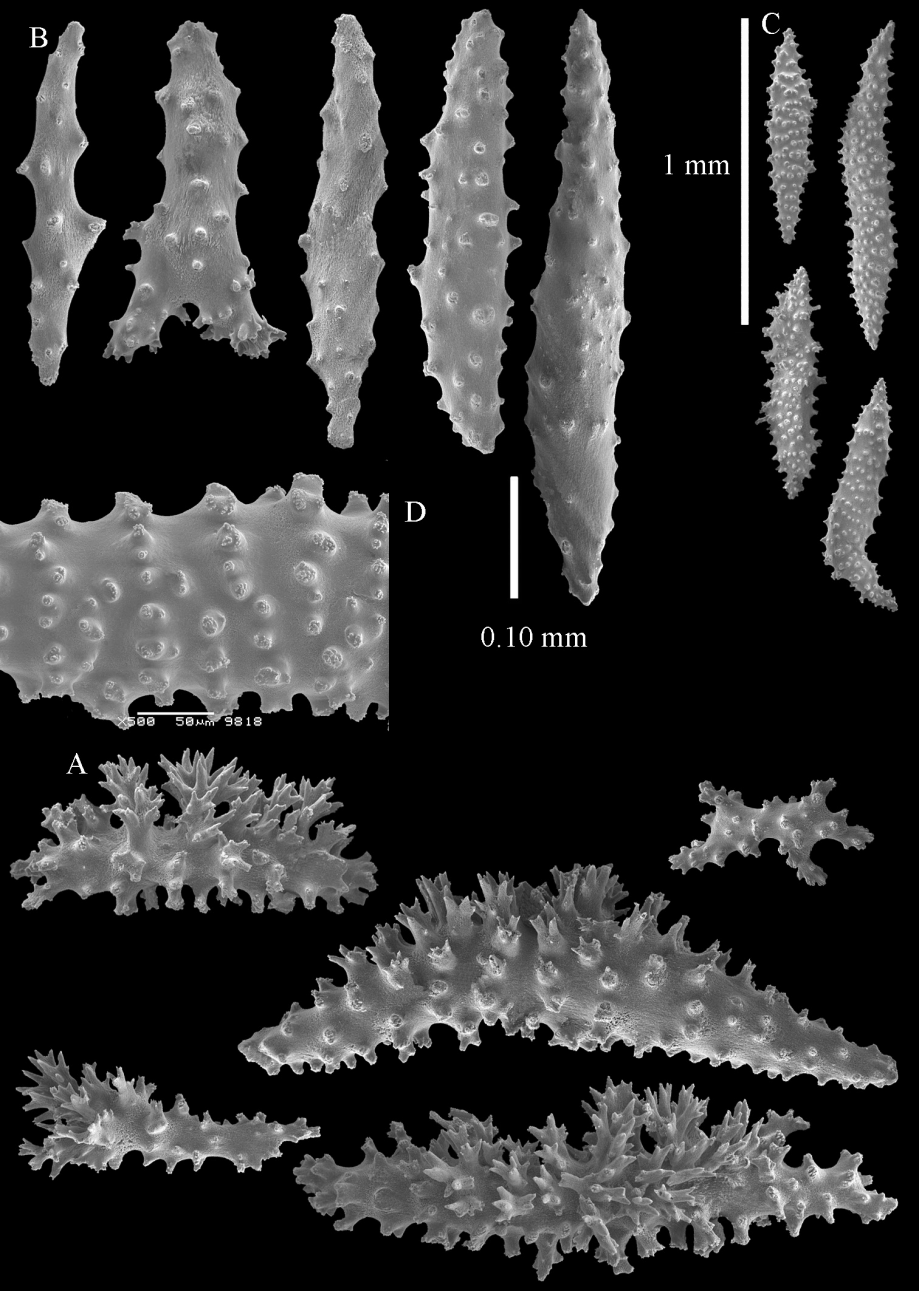
*Litophyton acuticonicum* (Verseveldt, 1974), ZMTAU Co 26239. **A** sclerites of surface layer base of stalk **B–C** sclerites of interior base of stalk **D** tubercles on spindle. Scale at **B** also applies to **A**.

**Figure 12.**
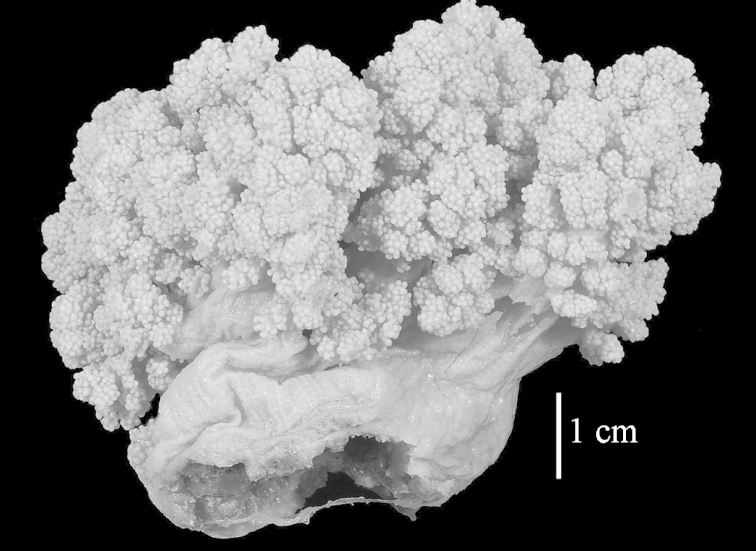
*Litophyton arboreum* Forskål, 1775, neotype ZMTAU Co 26246.

**Figure 13.**
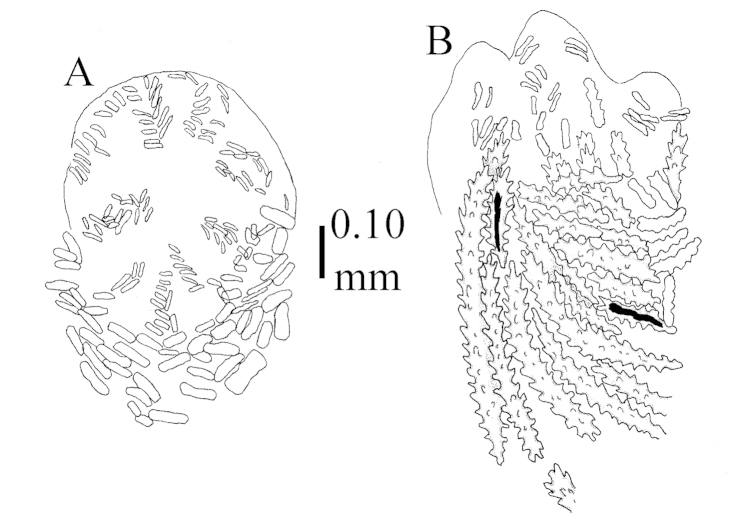
*Litophyton arboreum* Forskål, 1775, neotype ZMTAU Co 26246, polyp armature. **A** adaxial view **B** lateral view.

**Figure 14.**
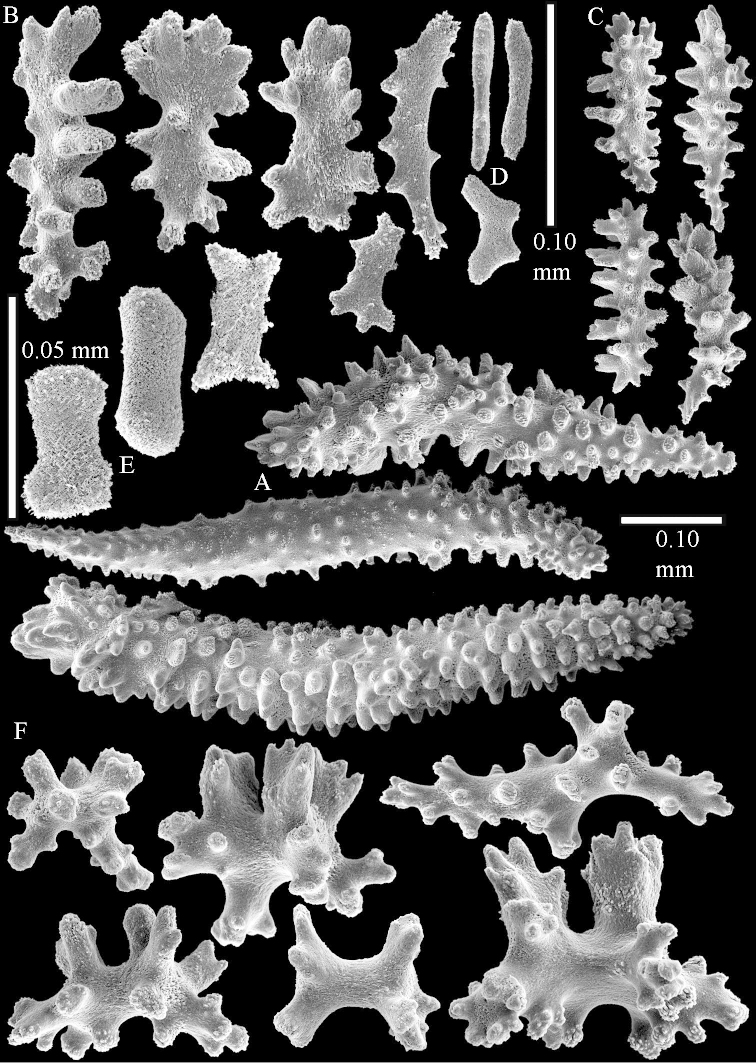
*Litophyton arboreum* Forskål, 1775, neotype ZMTAU Co 26246. **A** spindles of supporting bundle **B** small polyp body sclerites **C** large polyp body spindles **D** tentacle rodlets **E** polyp stalk scales **F** sclerites surface layer top of stalk. Scale at **A** also applies to **C**, scale at **D** also to **B** and **F**.

**Figure 15.**
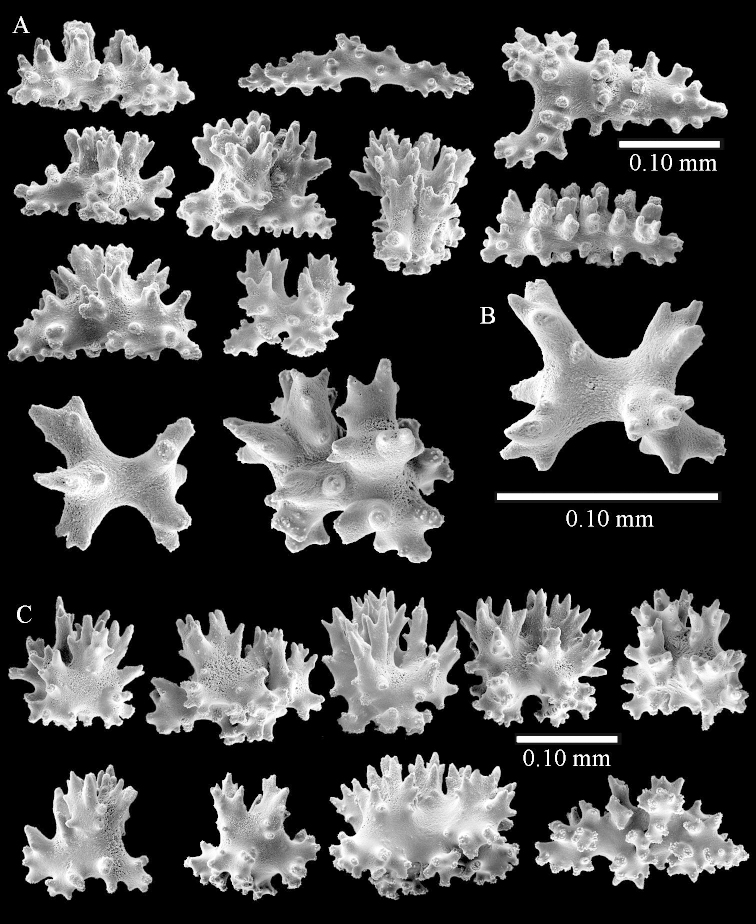
*Litophyton arboreum* Forskål, 1775, neotype ZMTAU Co 26246. **A** sclerites of surface layer top of stalk **B–C** sclerites of surface layer base of stalk.

**Figure 16.**
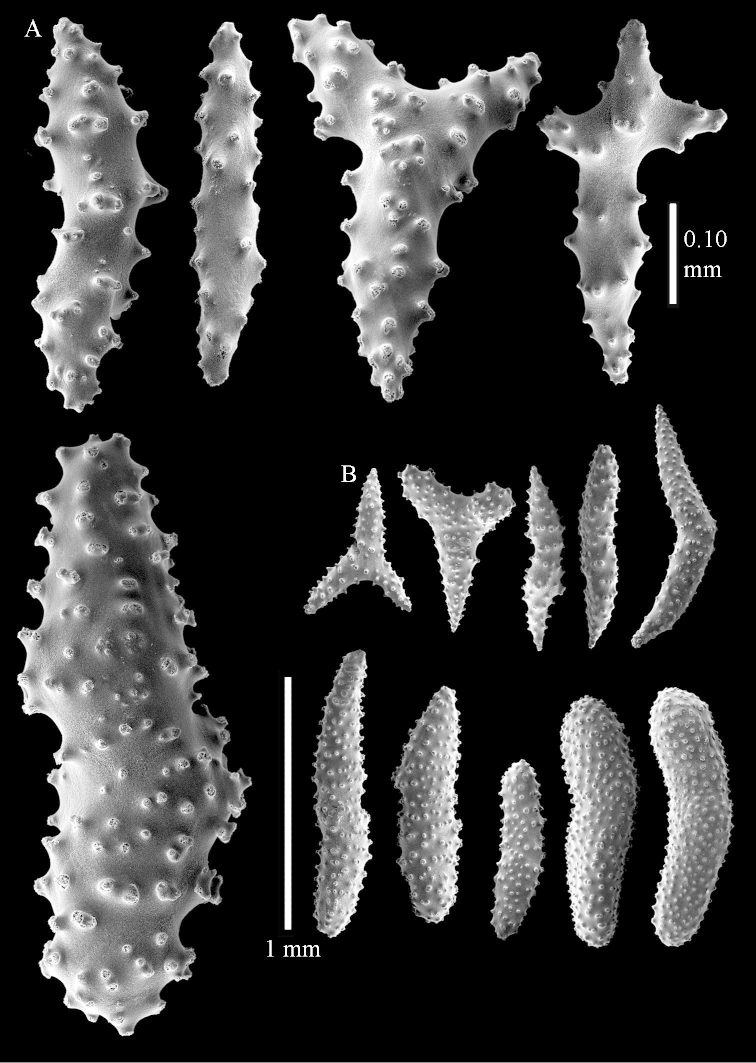
*Litophyton arboreum* Forskål, 1775, neotype ZMTAU Co 26246. **A–B** sclerites of interior base of stalk.

**Figure 17.**
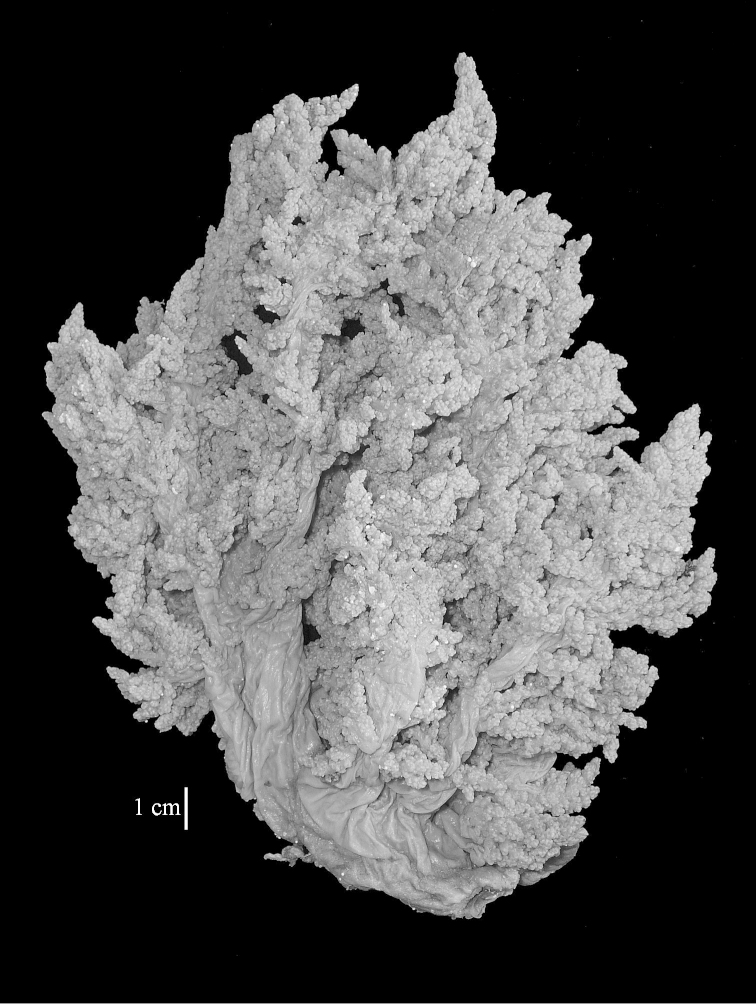
*Litophyton bumastum* (Verseveldt, 1973), holotype RMNH Coel. 8045.

**Figure 18.**
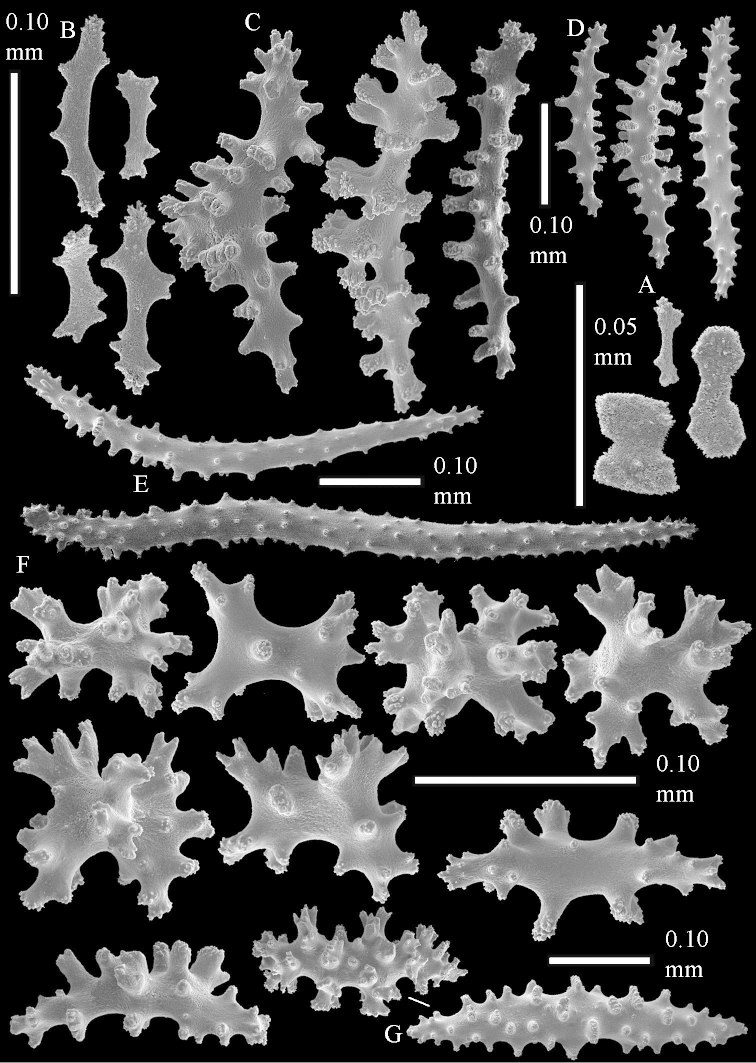
*Litophyton bumastum* (Verseveldt, 1973), holotype RMNH Coel. 8045. **A–B** tentacle rodlets **C–D** polyp body sclerites **E** spindles of supporting bundle **F–G** sclerites of surface layer top of stalk. Scale at **B** also applies to **C**.

**Figure 19.**
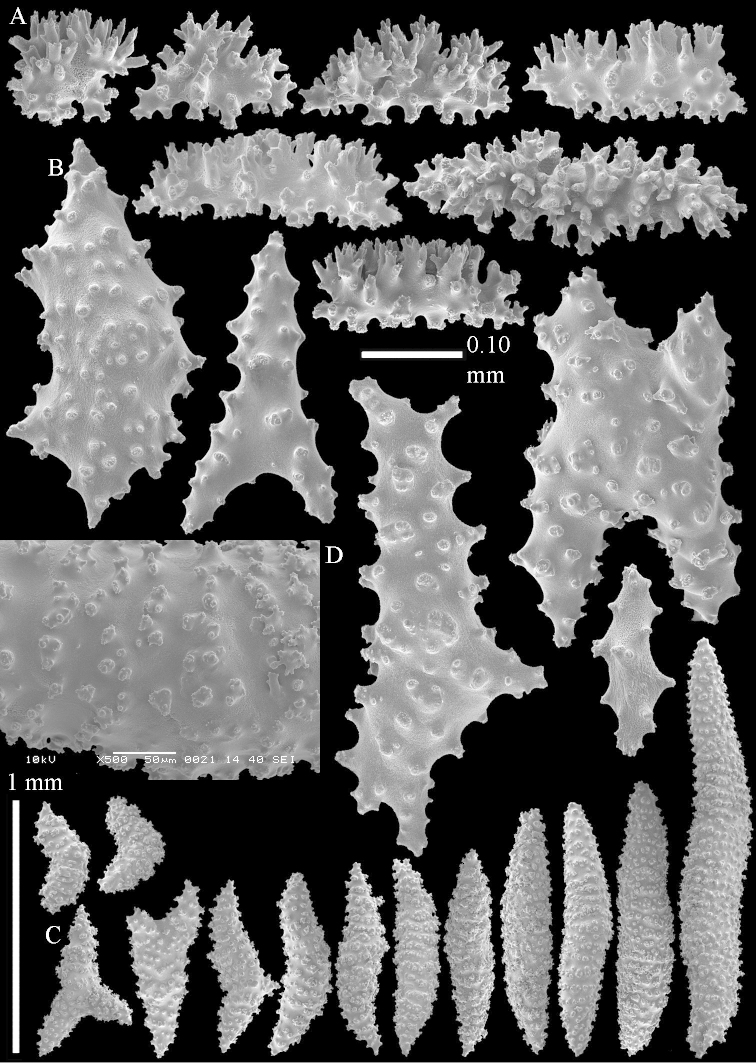
*Litophyton bumastum* (Verseveldt, 1973), holotype RMNH Coel. 8045. **A** sclerites of surface layer base of stalk **B–C** sclerites of interior base of stalk **D** tubercles on spindle. Scale at **B** also applies to **A**.

**Figure 20.**
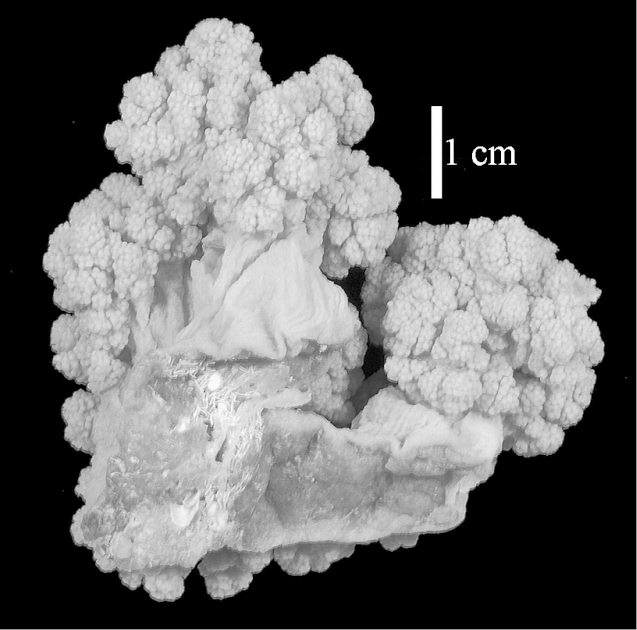
*Litophyton chabrolii* (Andouin, 1828), neotype ZMTAU Co 26244.

**Figure 21.**
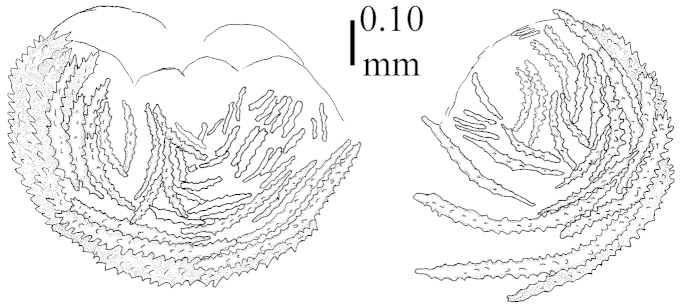
*Litophyton chabrolii* (Andouin, 1828), neotype ZMTAU Co 26244. Polyp armature, lateral views.

**Figure 22.**
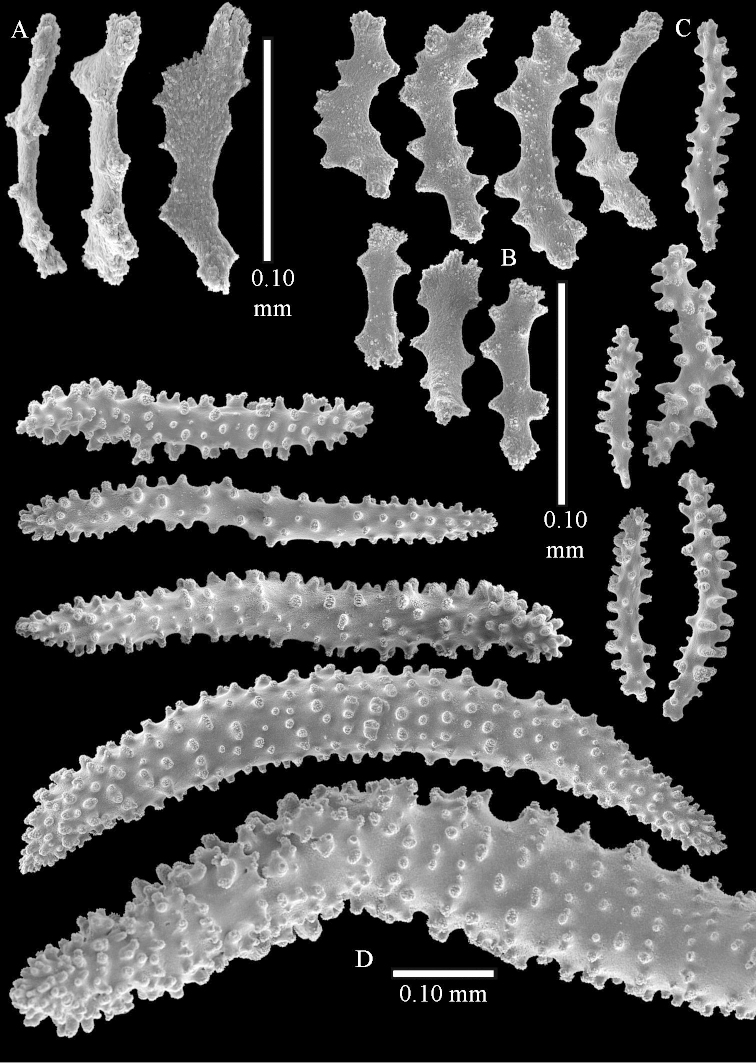
*Litophyton chabrolii* (Andouin, 1828), neotype ZMTAU Co 26244. **A** tentacle rodlets **B–C** polyp body sclerites **D** spindles of supporting bundle. Scale at **D** also applies to **C**.

**Figure 23.**
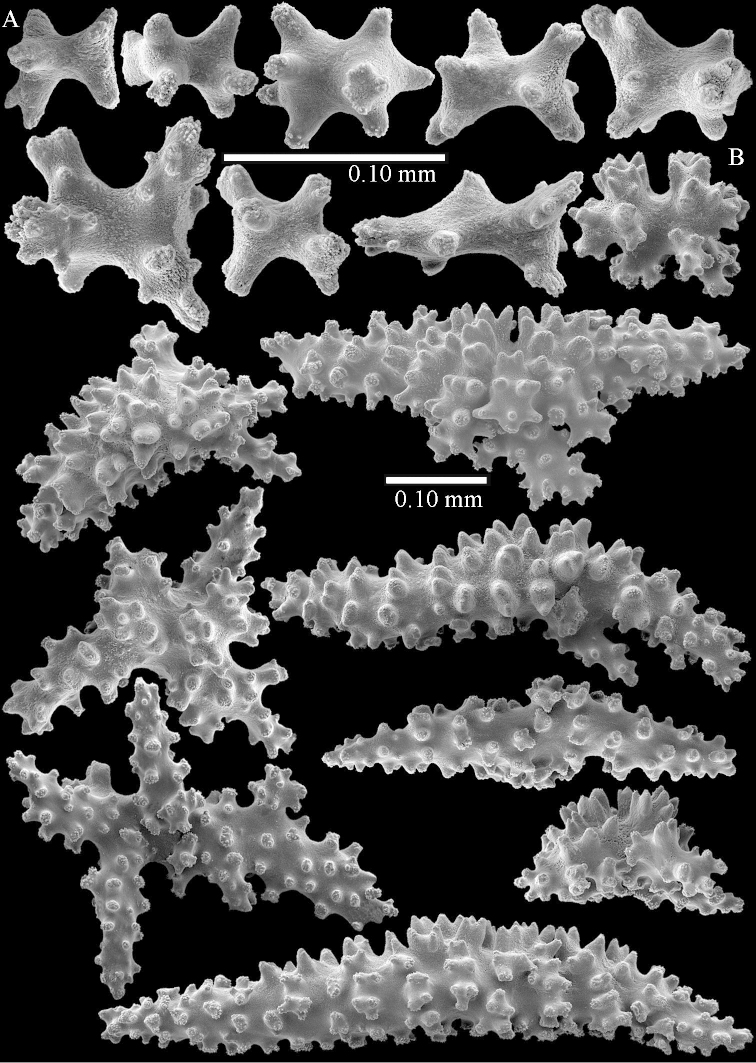
*Litophyton chabrolii* (Andouin, 1828), neotype ZMTAU Co 26244. **A–B** sclerites of surface layer top of stalk.

**Figure 24.**
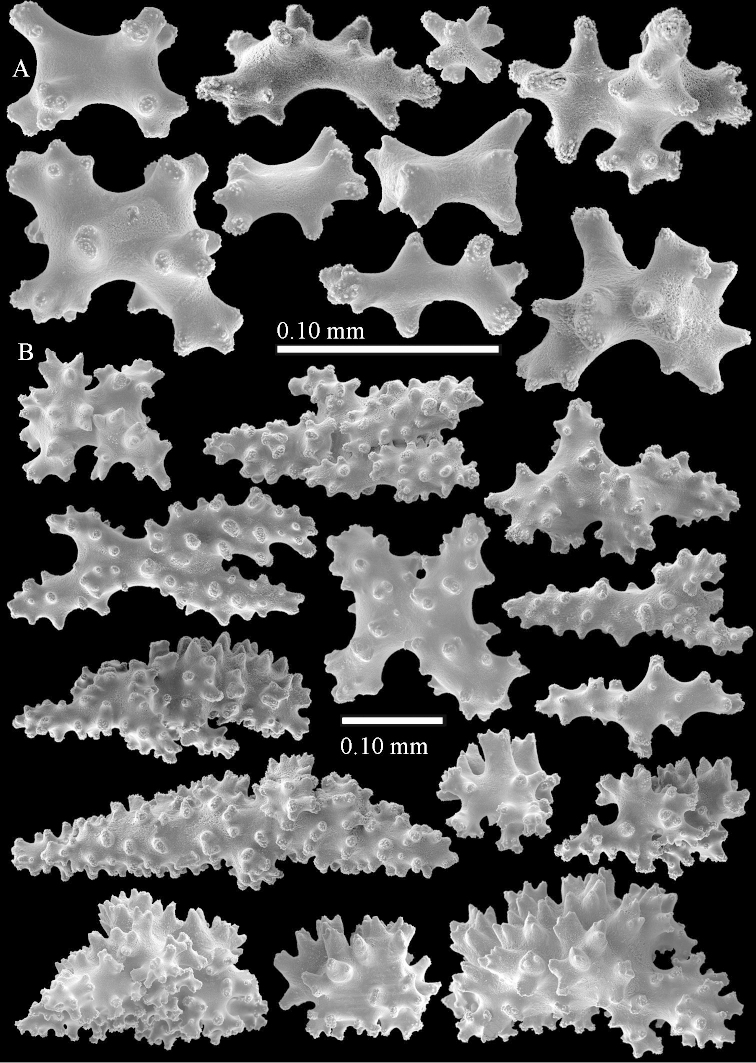
*Litophyton chabrolii* (Andouin, 1828), neotype ZMTAU Co 26244. **A–B** sclerites of surface layer base of stalk.

**Figure 25.**
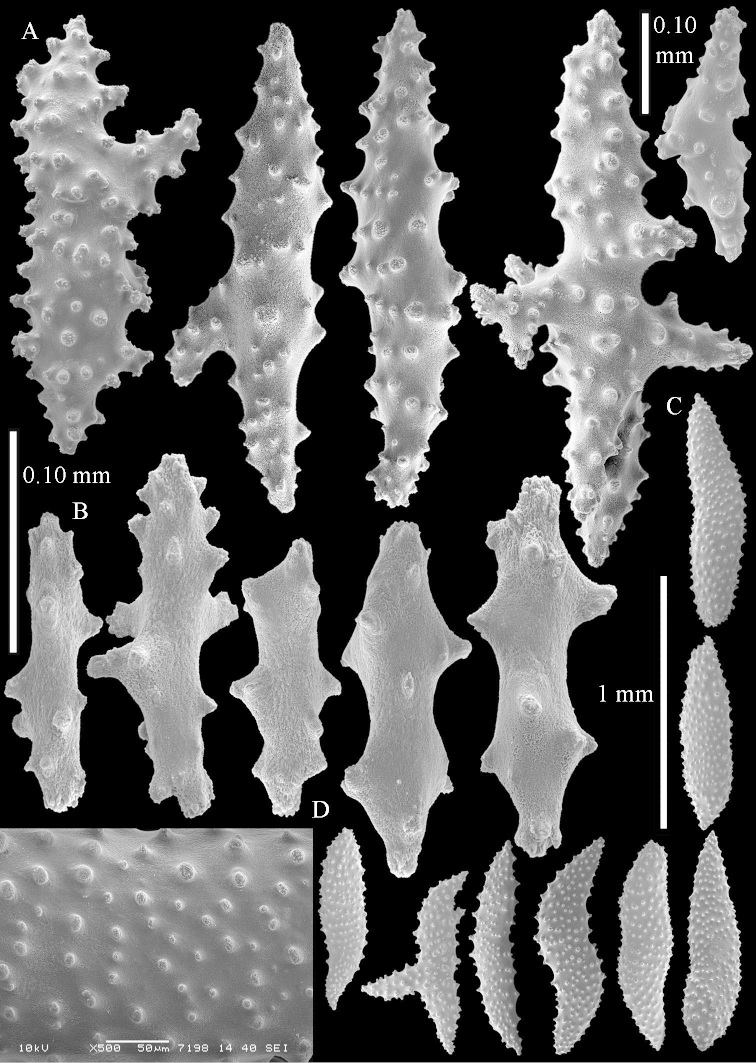
*Litophyton chabrolii* (Andouin, 1828), neotype ZMTAU Co 26244. **A–C** sclerites of interior base of stalk **D** tubercles on spindle.

**Figure 26.**
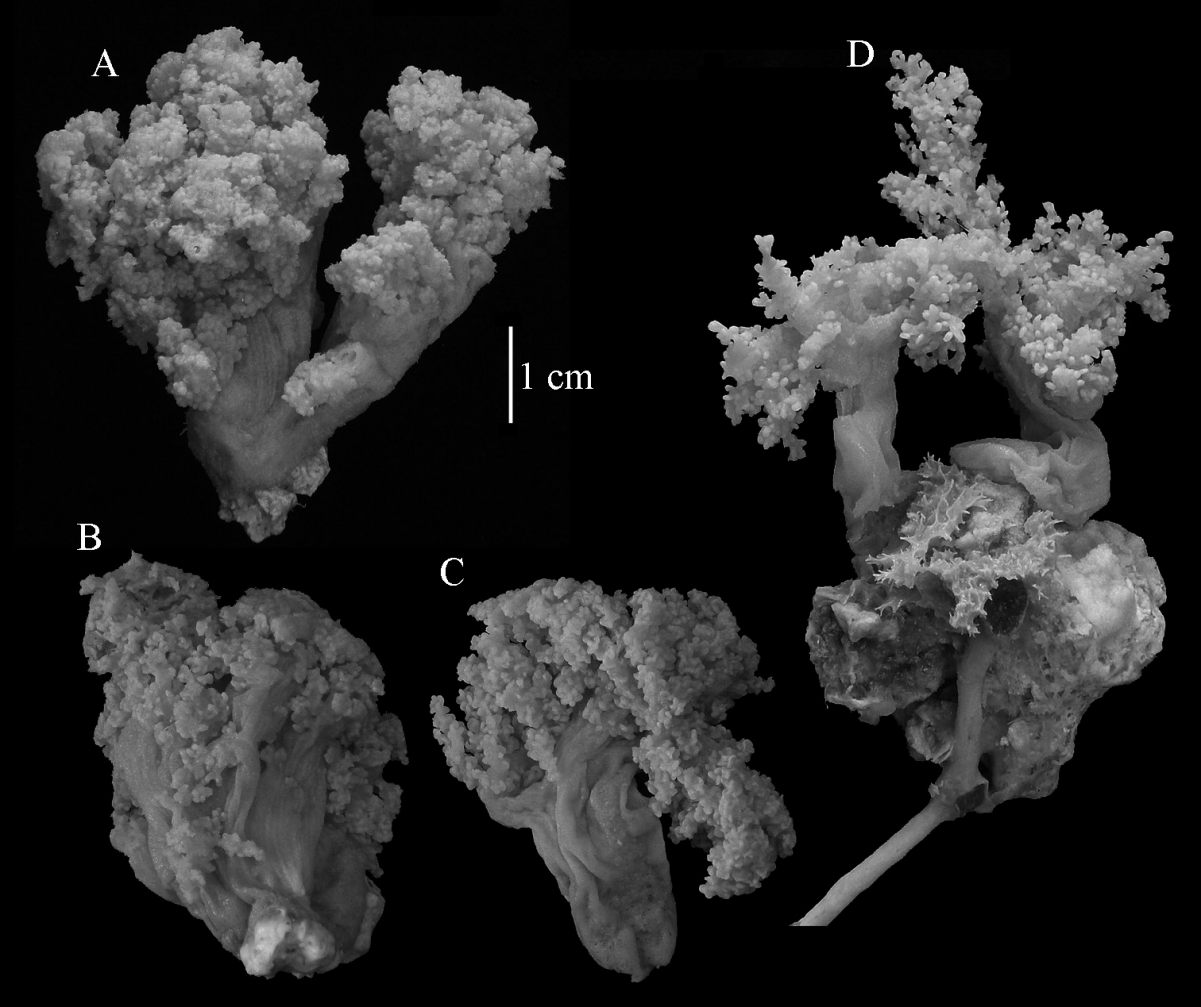
*Litophyton curvum* sp. n., **A** holotype ZMTAU Co 28555 **B–C** paratypes ZMTAU Co 28555 **D** paratype ZMTAU Co 26225.

**Figure 27.**
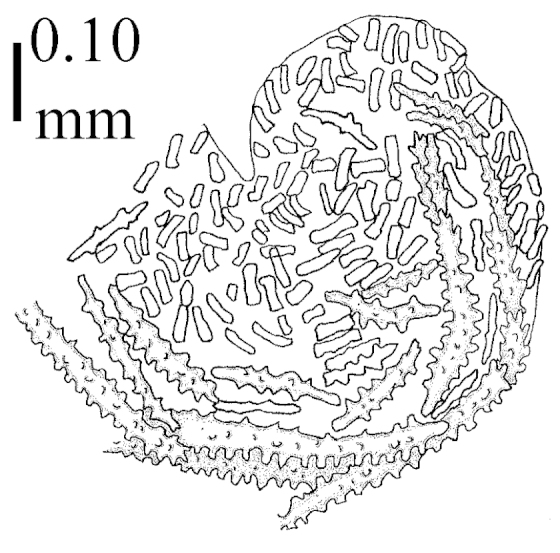
*Litophyton curvum* sp. n., holotype ZMTAU Co 28555. Polyp armature, lateral view.

**Figure 28.**
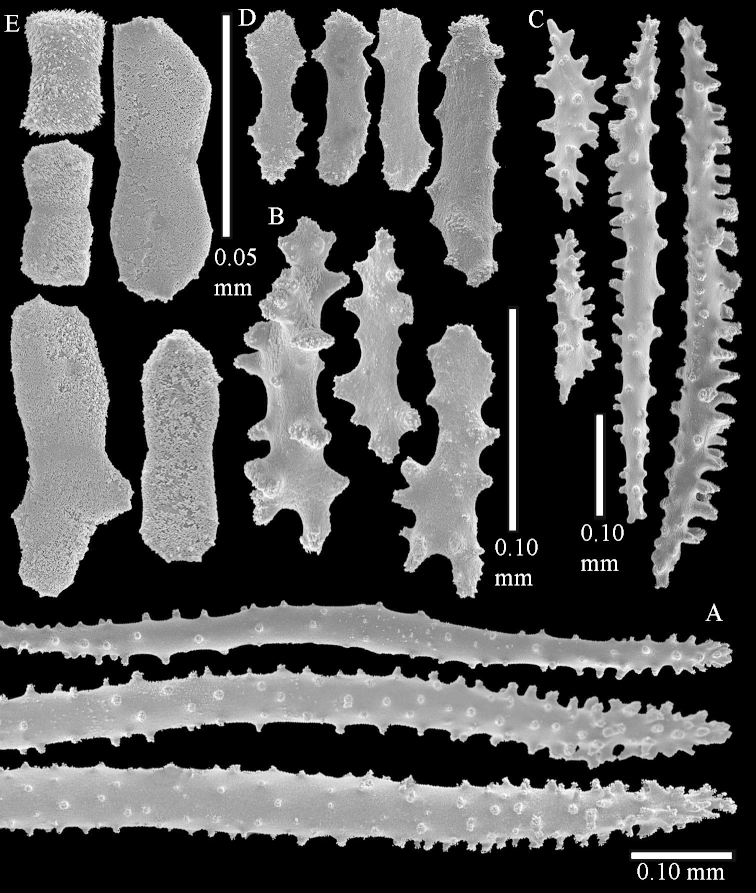
*Litophyton curvum* sp. n., holotype ZMTAU Co 28555. **A** spindles of supporting bundle **B–C** polyp body sclerites **D** tentacle rodlets **E** polyp stalk scales.

**Figure 29.**
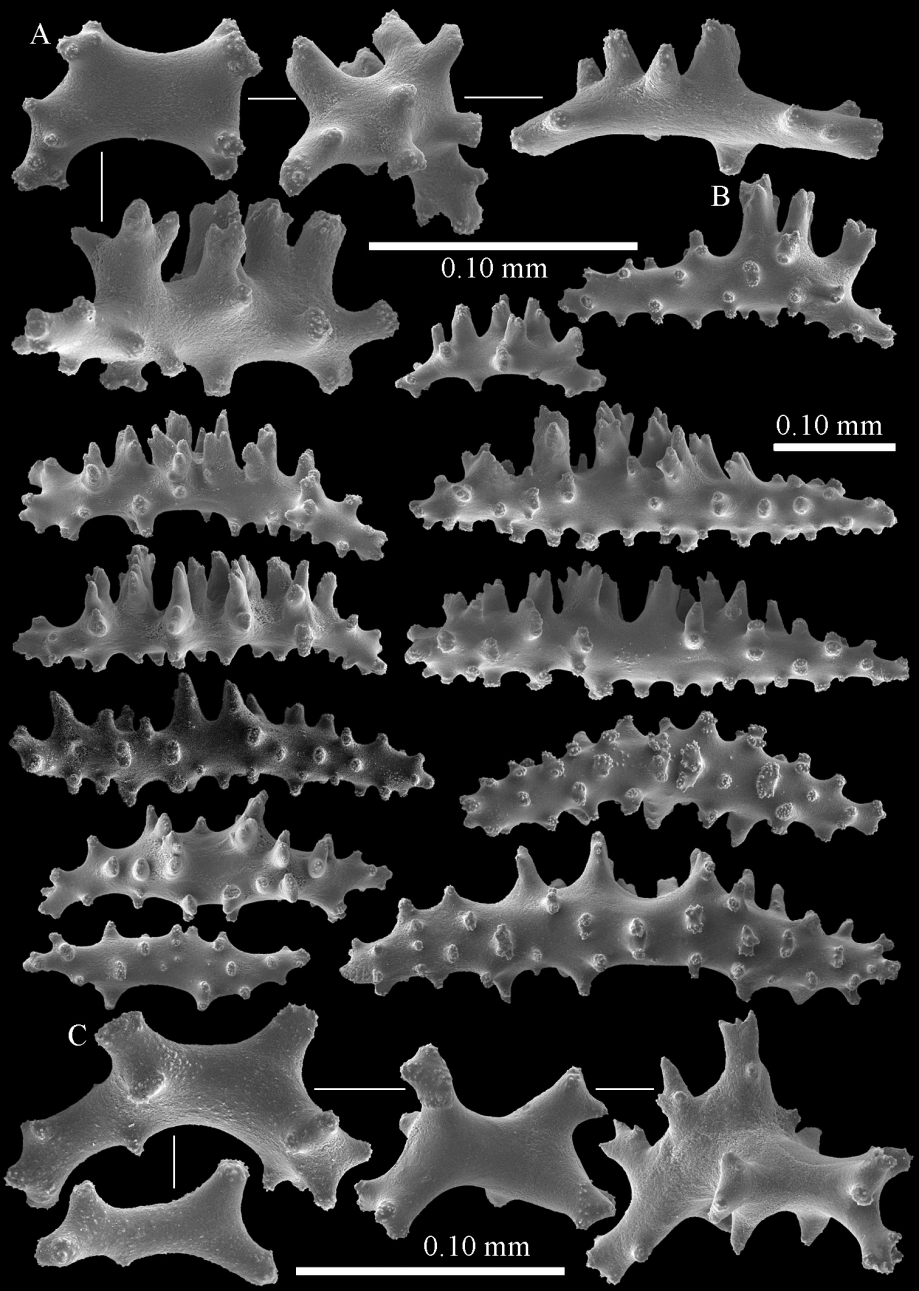
*Litophyton curvum* sp. n., holotype ZMTAU Co 28555. **A–B** sclerites of surface layer top of stalk **C** sclerites of surface layer base of stalk.

**Figure 30.**
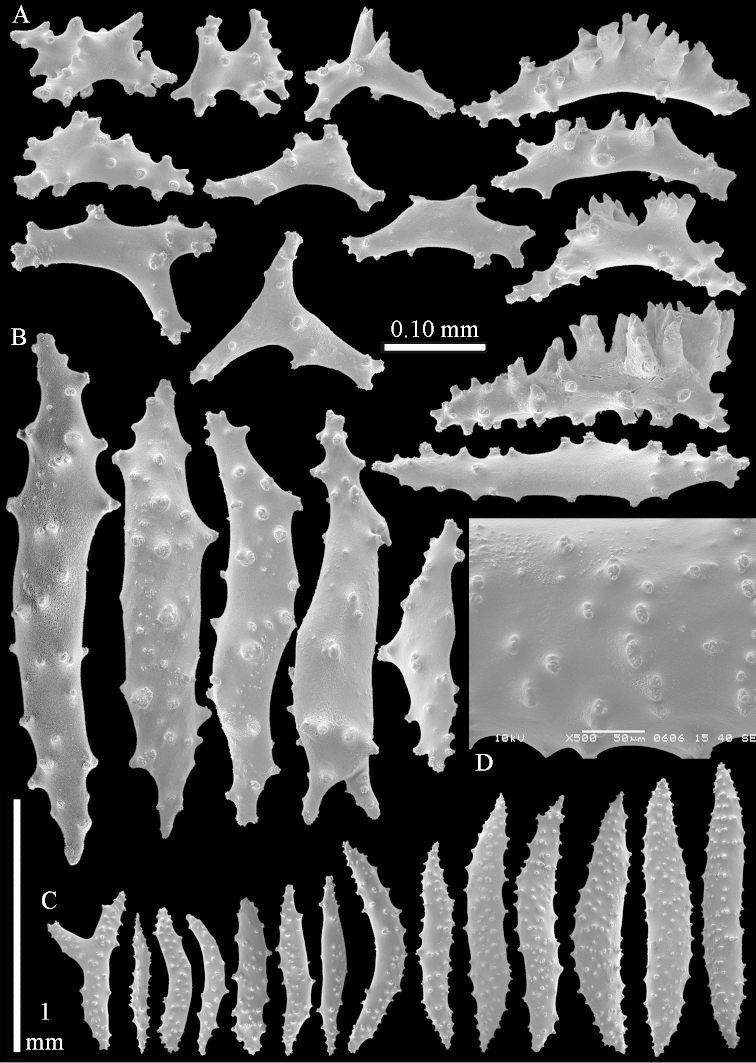
*Litophyton curvum* sp. n., holotype ZMTAU Co 28555. **A** sclerites of surface layer base of stalk **B–C** sclerites of interior base of stalk **D** tubercles on spindle.

**Figure 31.**
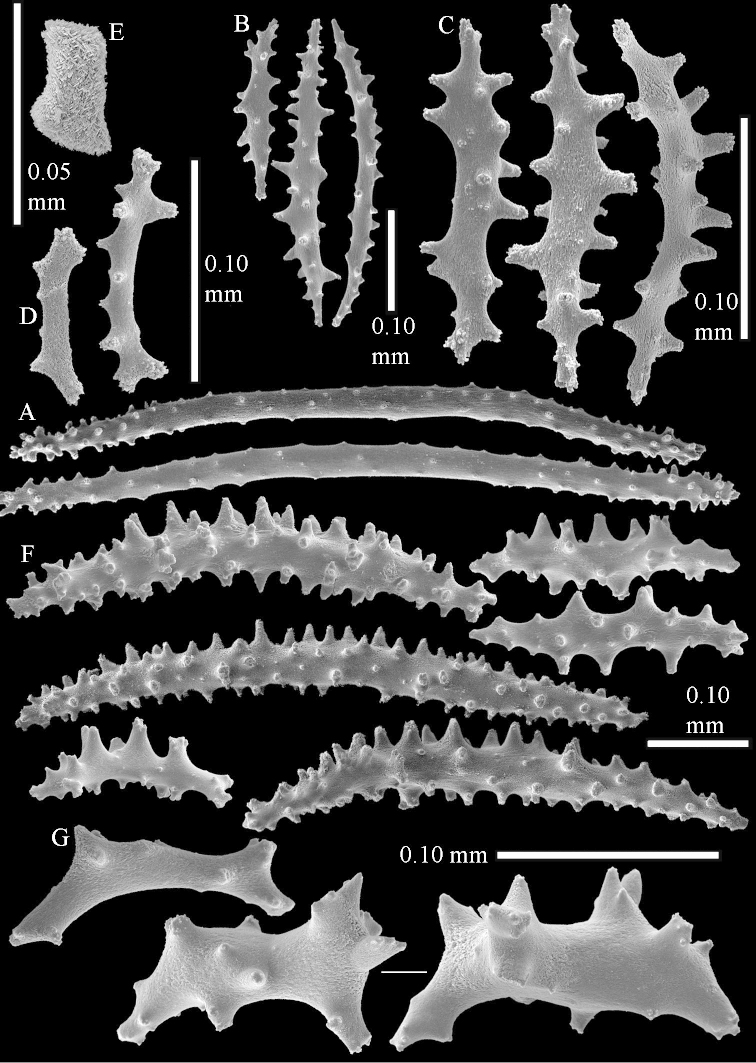
*Litophyton curvum* sp. n., paratype ZMTAU Co 28552. **A** spindles of supporting bundle **B–C** polyp body sclerites **D** tentacle rodlets **E** polyp stalk scale **F–G** sclerites of surface layer top of stalk.

**Figure 32.**
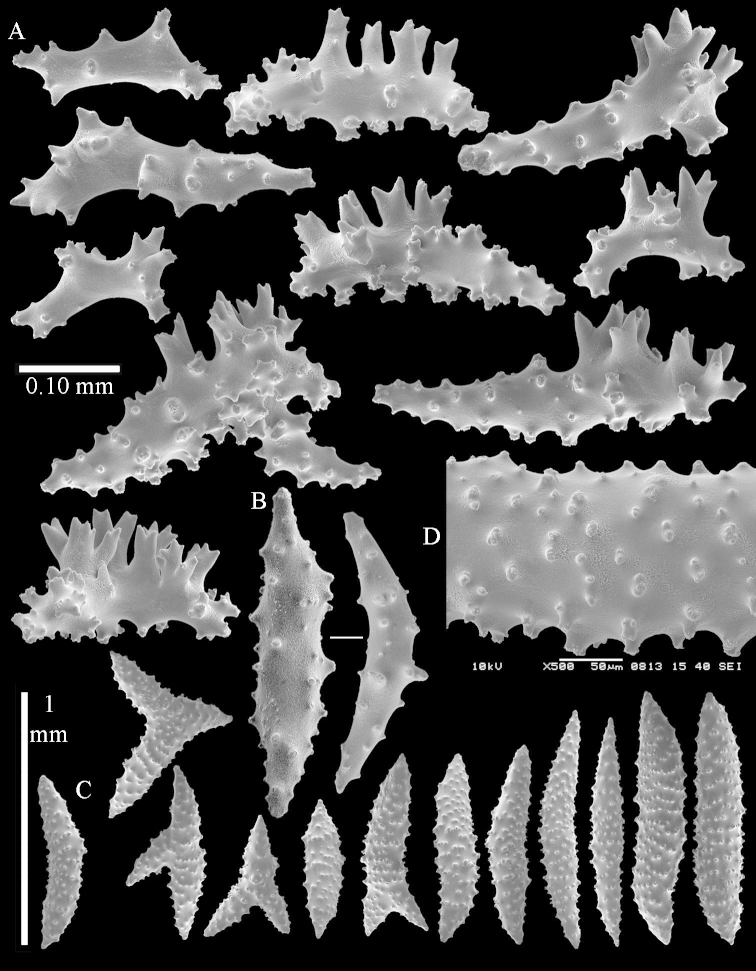
*Litophyton curvum* sp. n., paratype ZMTAU Co 28552. **A** sclerites of surface layer base of stalk **B–C** sclerites of interior base of stalk **D** tubercles on spindle.

**Figure 33.**
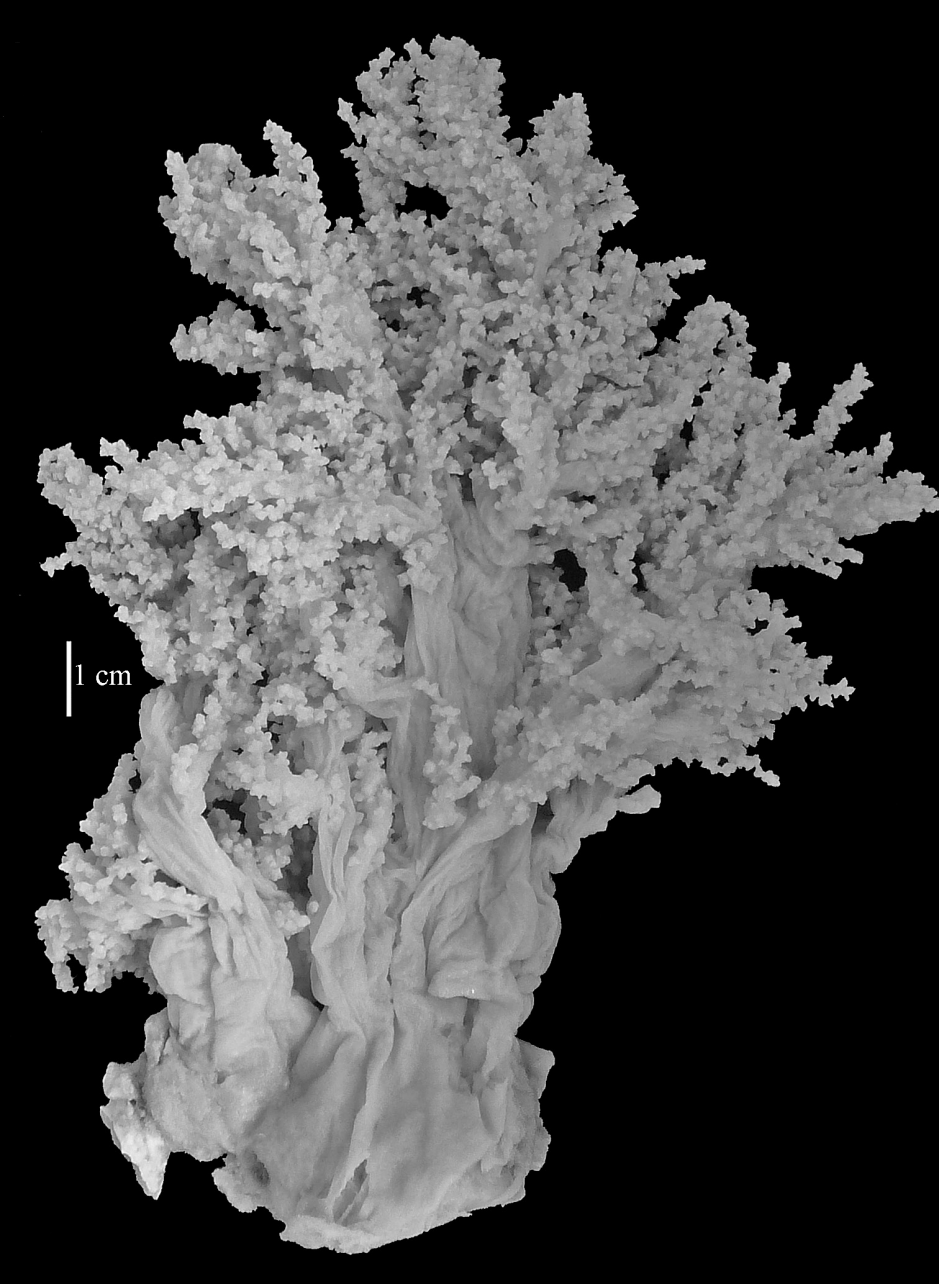
*Litophyton filamentosum* Verseveldt, 1973, holotype RMNH Coel. 8046.

**Figure 34.**
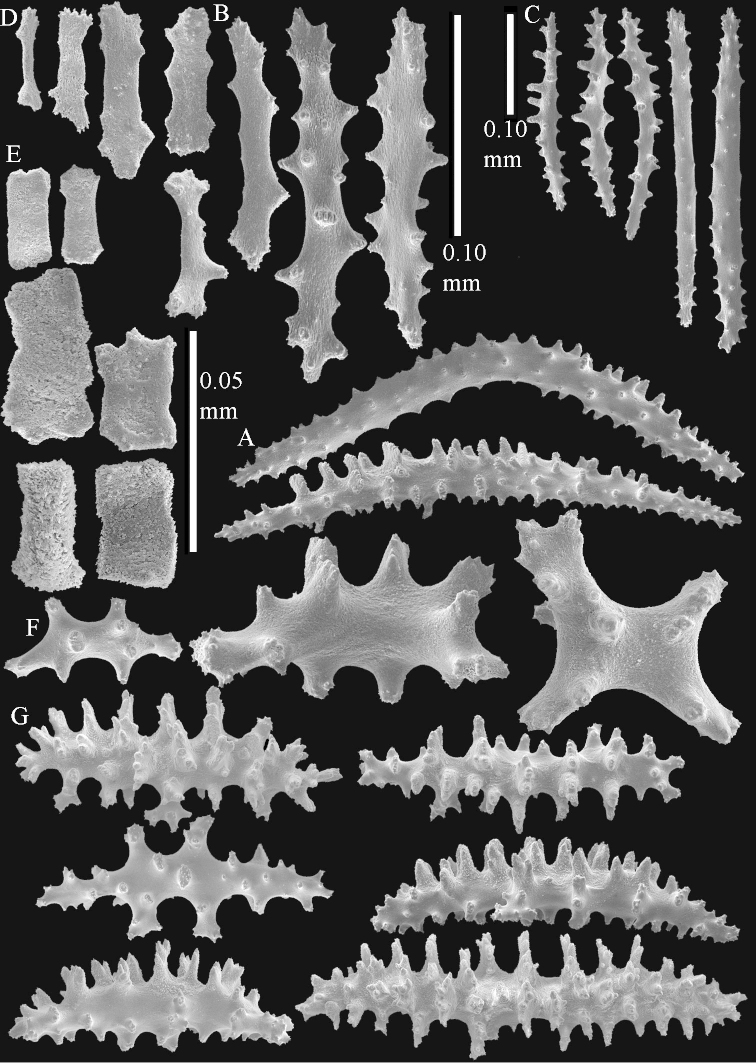
*Litophyton filamentosum*Verseveldt, 1973, holotype RMNH Coel. 8046. **A** spindles of supporting bundle **B–C** polyp body sclerites **D** tentacle rodlets **E** polyp stalk scales **F–G** sclerites of surface layer top of stalk. Scale at **B** also applies to **D, F**, scale at **C** also to **A, G**.

**Figure 35.**
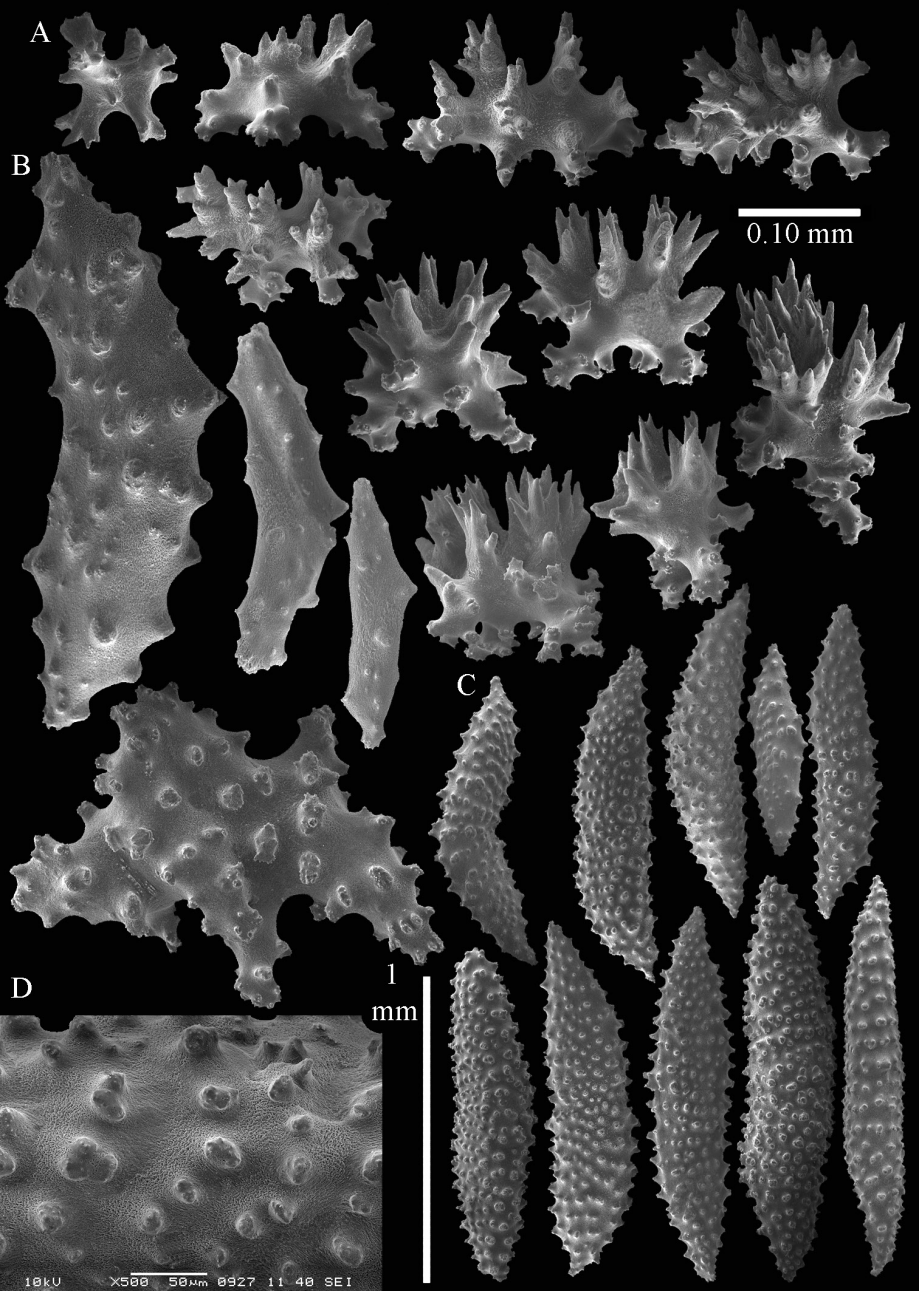
*Litophyton filamentosum* Verseveldt, 1973, holotype RMNH Coel. 8046. **A** sclerites of surface layer base of stalk **B–C** sclerites of interior base of stalk **D** tubercles on spindle. Scale at **A** also applies to **B**.

**Figure 36.**
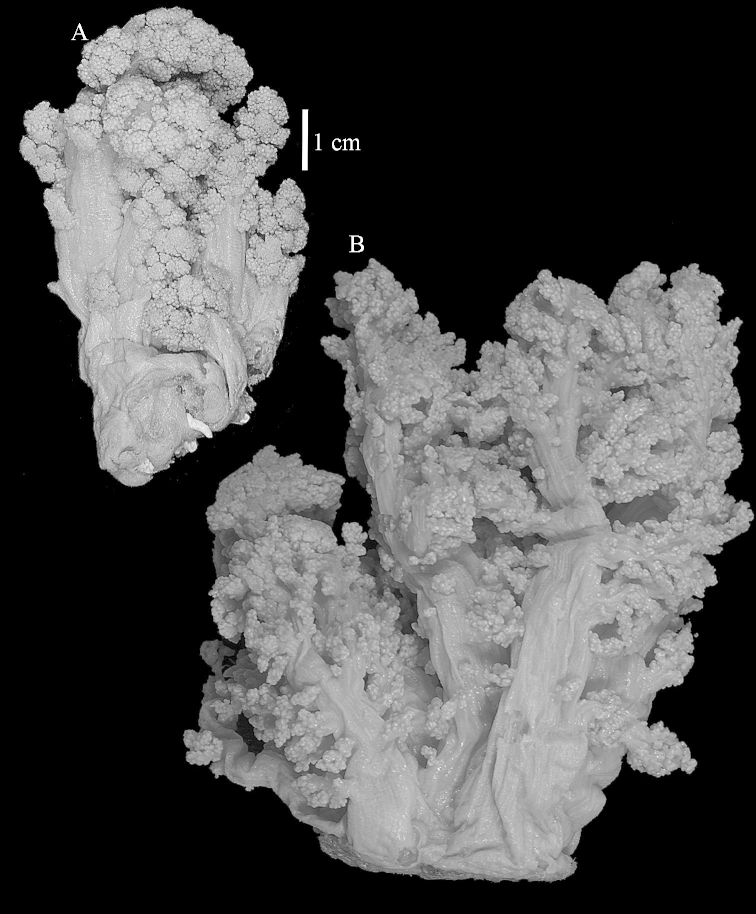
*Litophyton laevis* (Kükenthal, 1913). **A** holotype ZMB 6818 **B** ZMTAU Co 26126.

**Figure 37.**
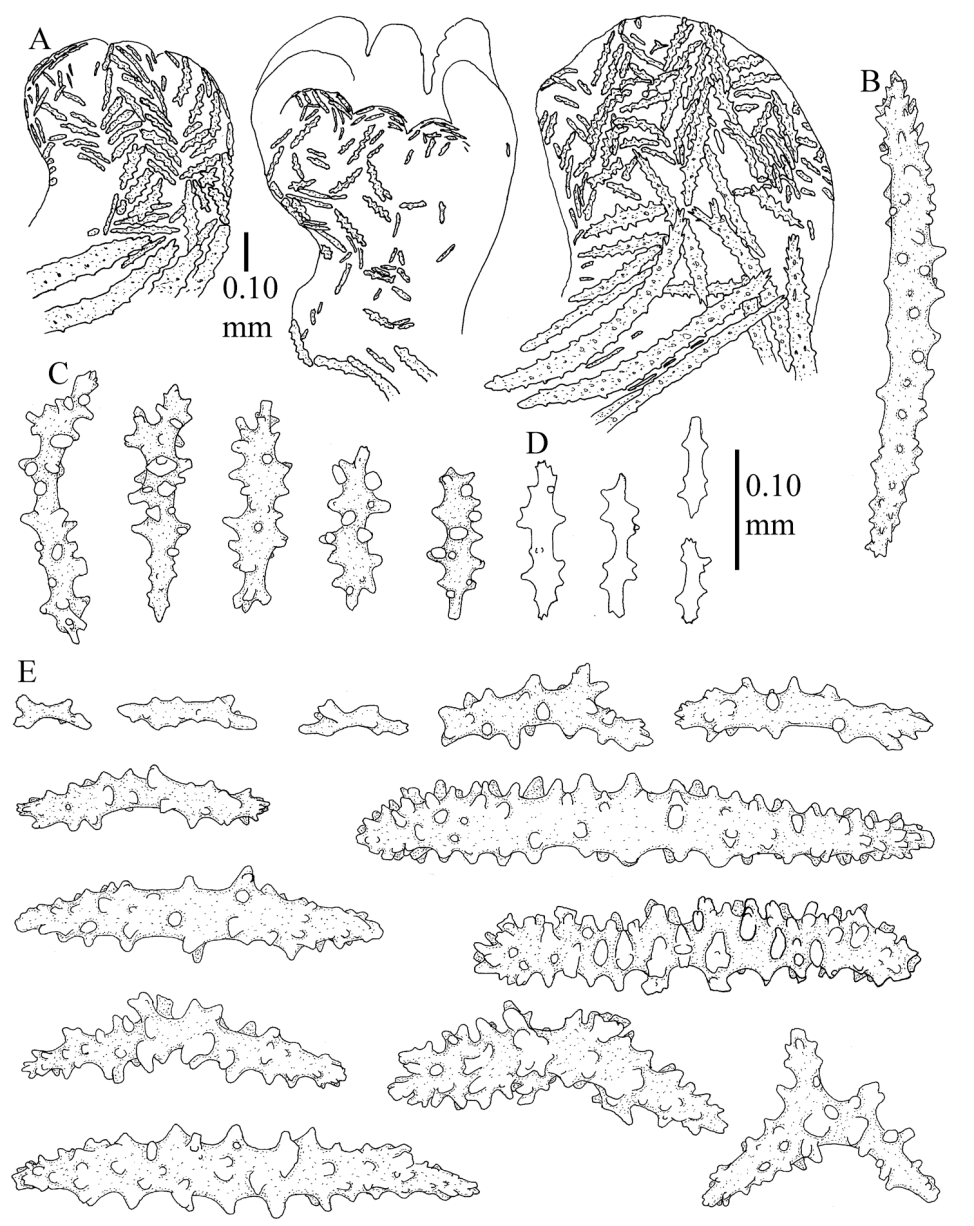
*Litophyton laevis* (Kükenthal, 1913), holotype ZMB 6818. **A** lateral, adaxial and abaxial views of polyp armature **B** supporting bundle spindle **C** polyp body sclerites **D** tentacle rodlets **E** sclerites, surface layer top of stalk. Scale at **A** only applies to **A**.

**Figure 38.**
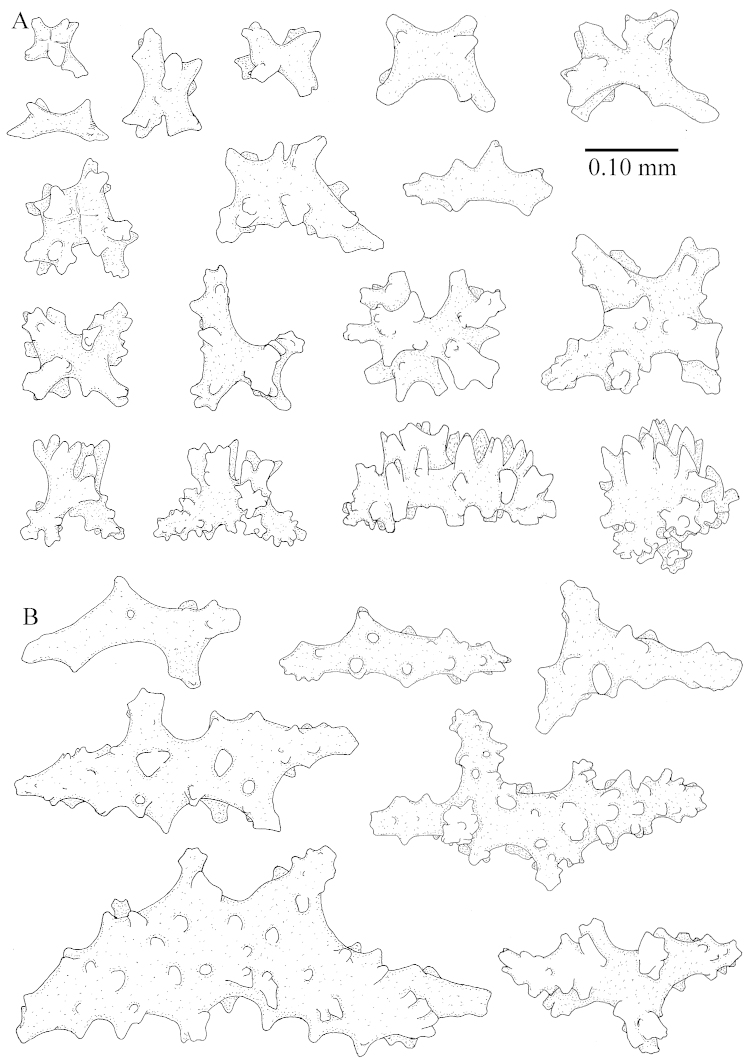
*Litophyton laevis* (Kükenthal, 1913), holotype ZMB 6818. **A** sclerites of surface layer base stalk **B** spindles of interior base of stalk.

**Figure 39.**
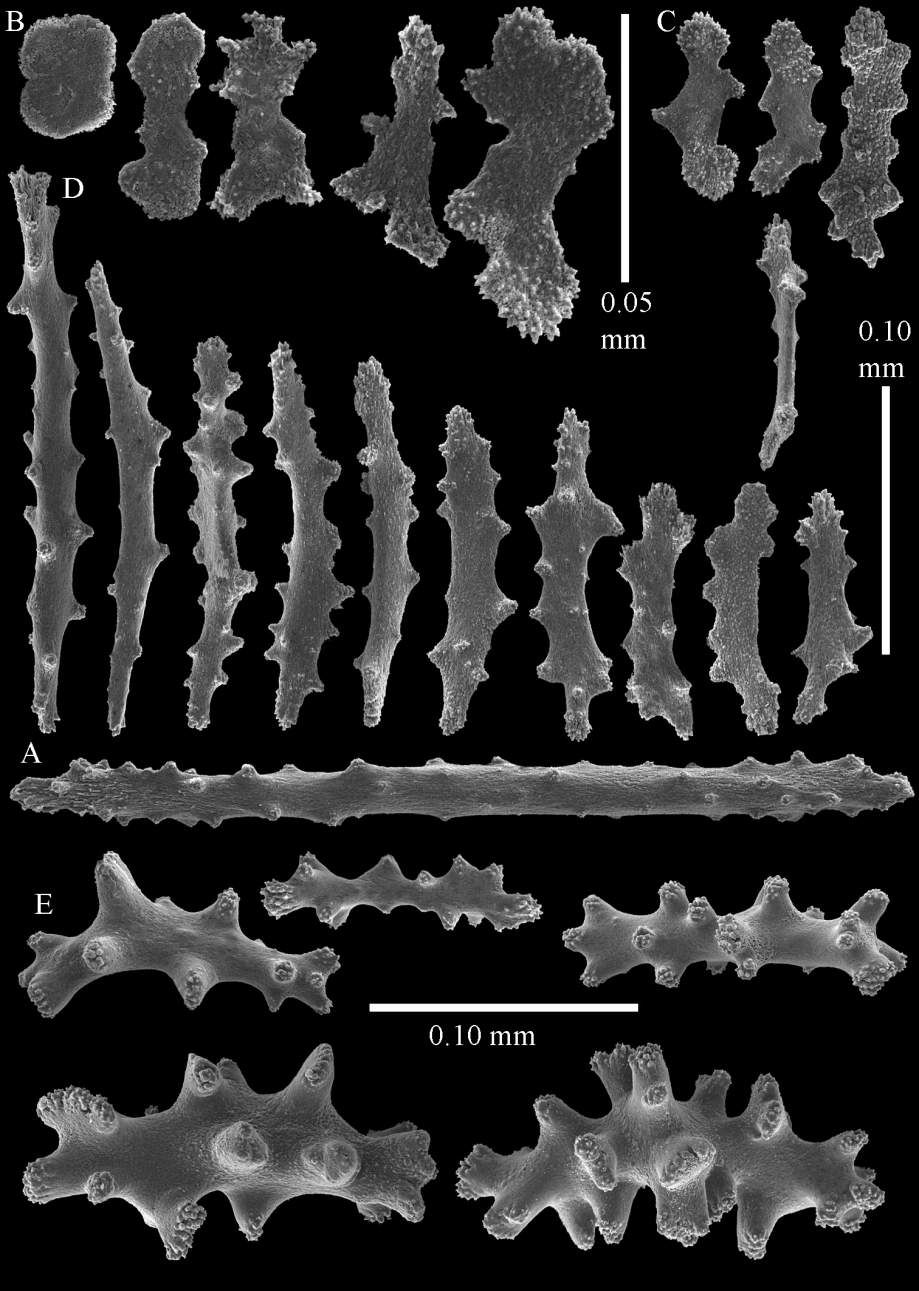
*Litophyton laevis* (Kükenthal, 1913), ZMTAU Co 26126. **A** spindle of supporting bundle **B–C** tentacle rodlets **D** polyp body sclerites **E** sclerites of surface layer top of stalk. Scale at **B** only applies to **B**.

**Figure 40.**
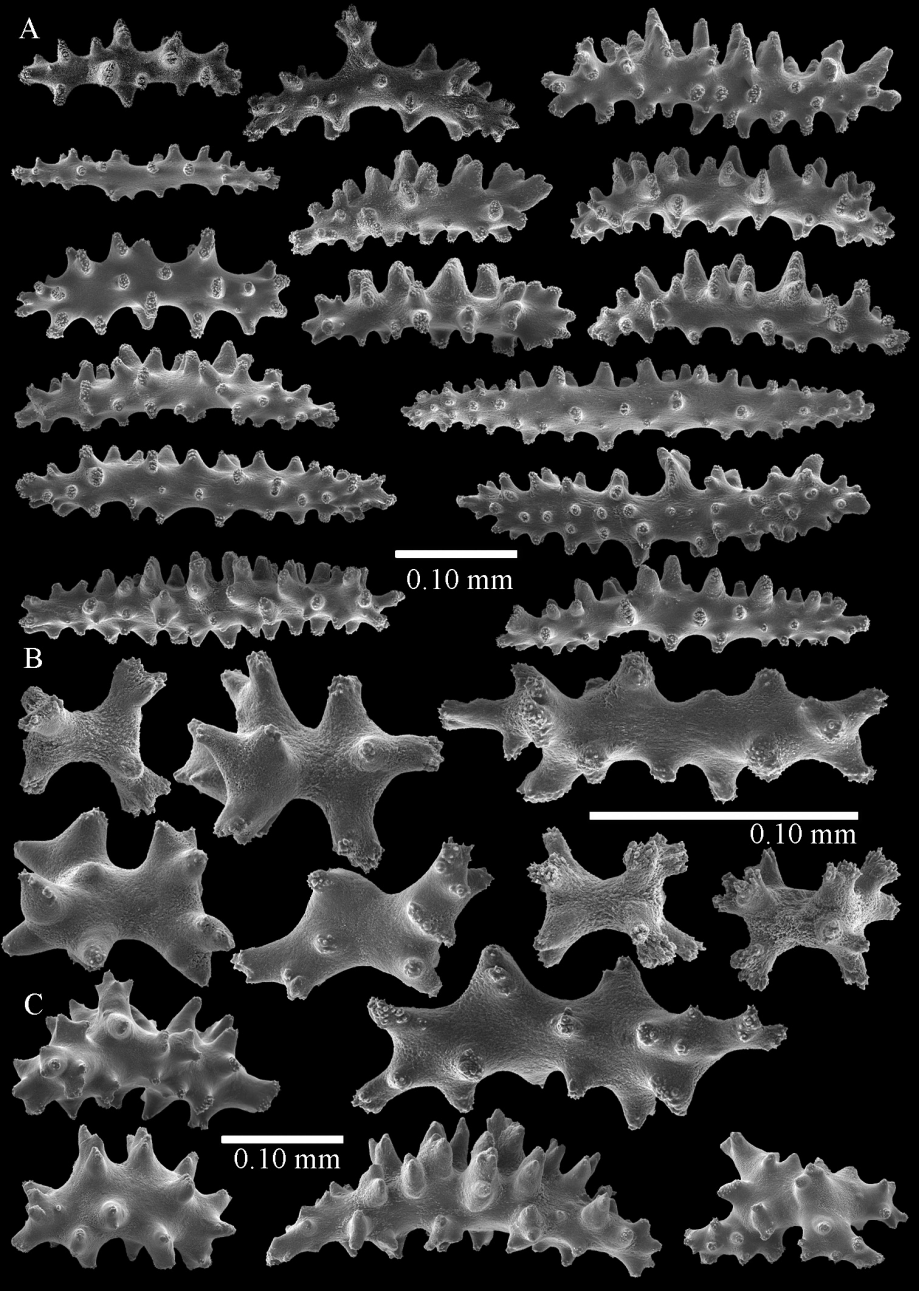
*Litophyton laevis* (Kükenthal, 1913), ZMTAU Co 26126. **A** sclerites of surface layer top of stalk **B–C** sclerites surface layer base of stalk.

**Figure 41.**
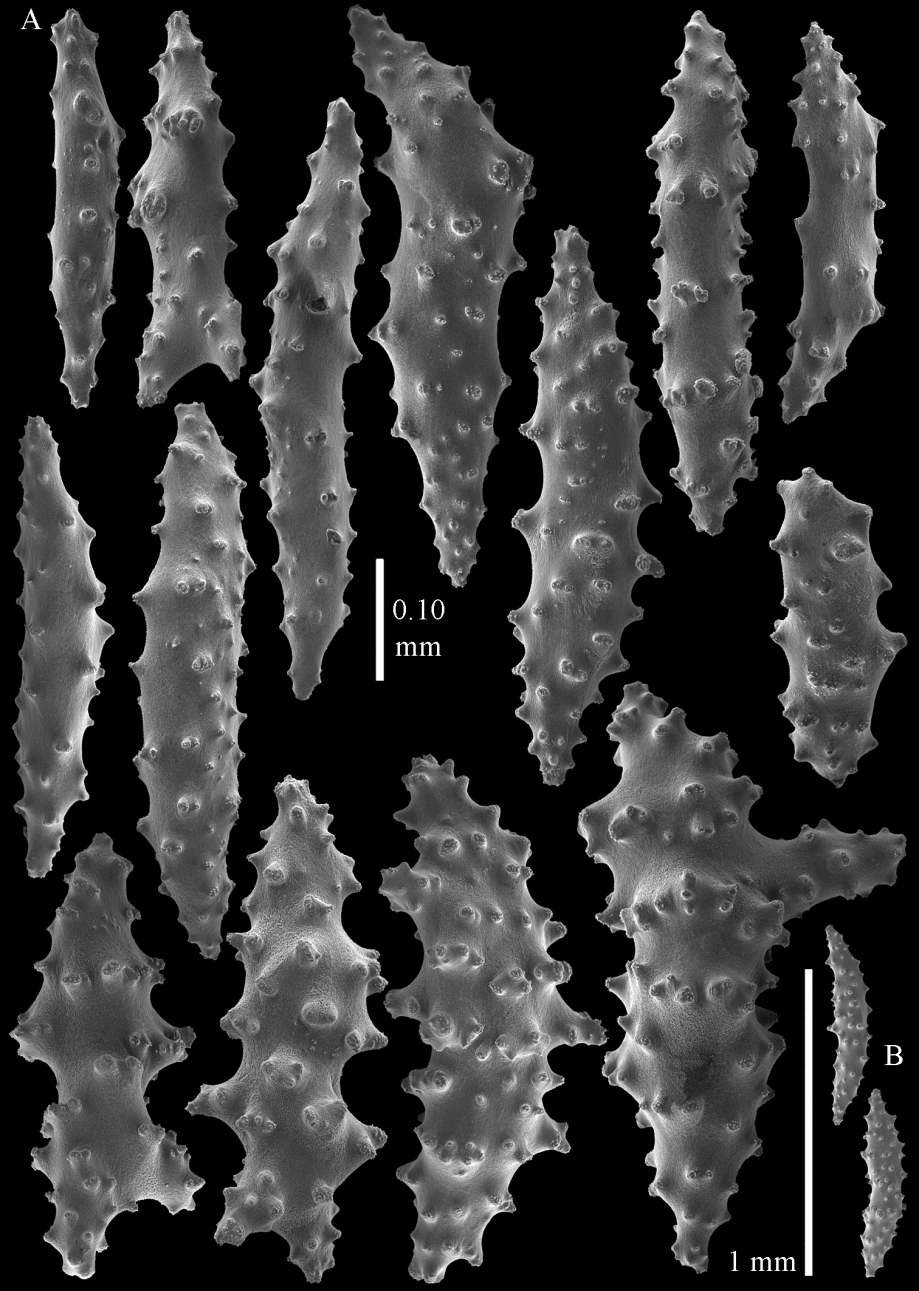
*Litophyton laevis* (Kükenthal, 1913), ZMTAU Co 26126. **A–B** spindles interior base of stalk.

**Figure 42.**
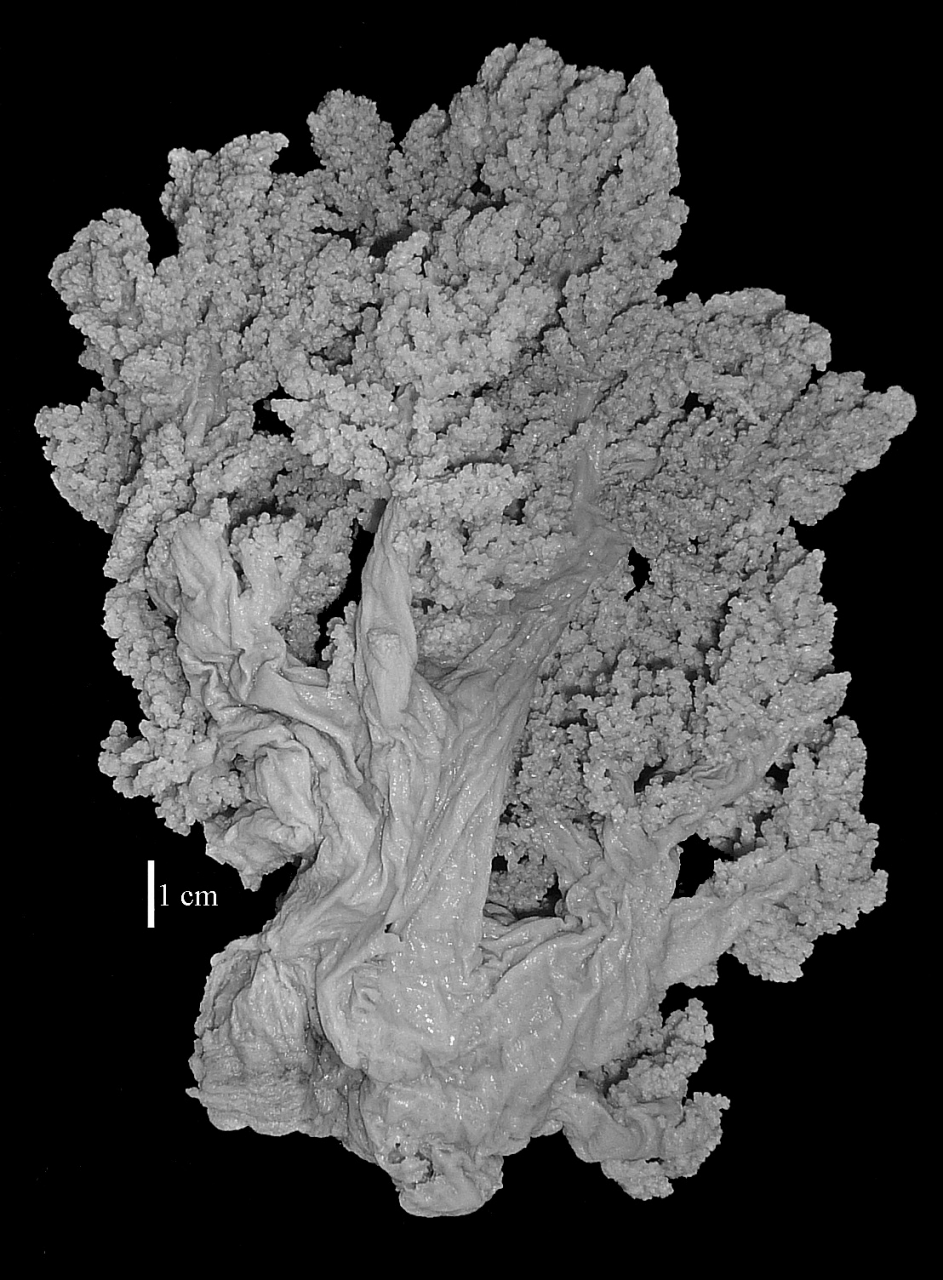
*Litophyton lanternarium* (Verseveldt, 1973), holotype RMNH Coel. 8052.

**Figure 43.**
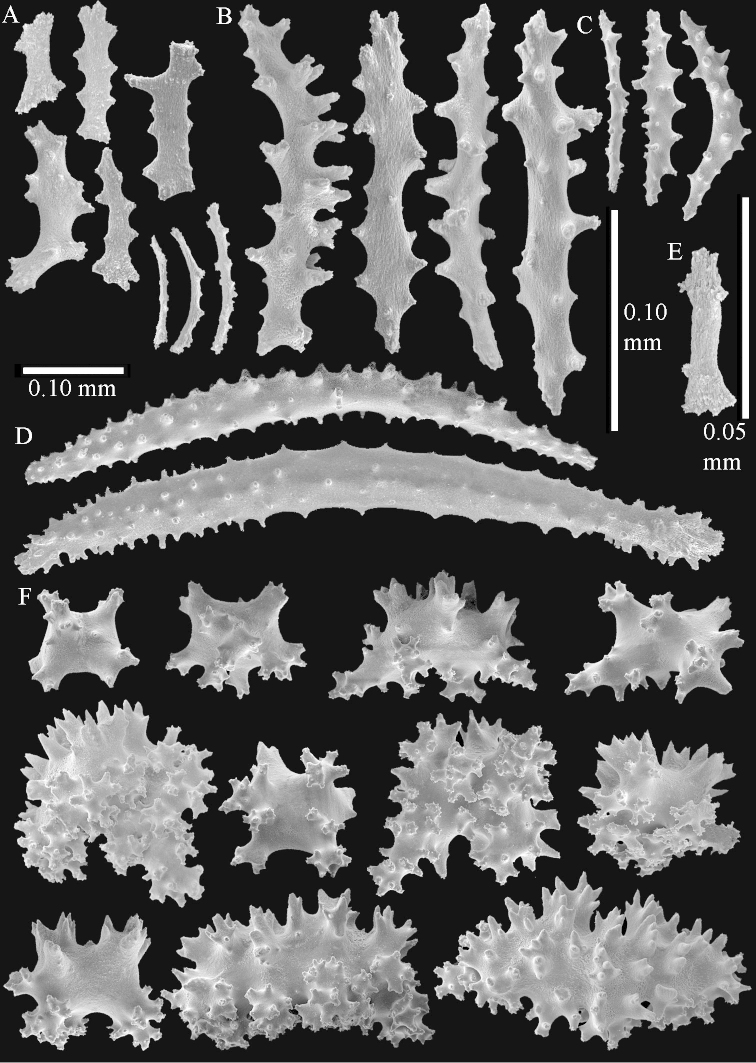
*Litophyton lanternarium* (Verseveldt, 1973), holotype RMNH Coel. 8052. **A** tentacle rodlets **B–C** polyp body sclerites **D** spindles of supporting bundle **E** polyp stalk rodlet **F** sclerites surface layer top of stalk. Scale at **D** also applies to **C** and **F**.

**Figure 44.**
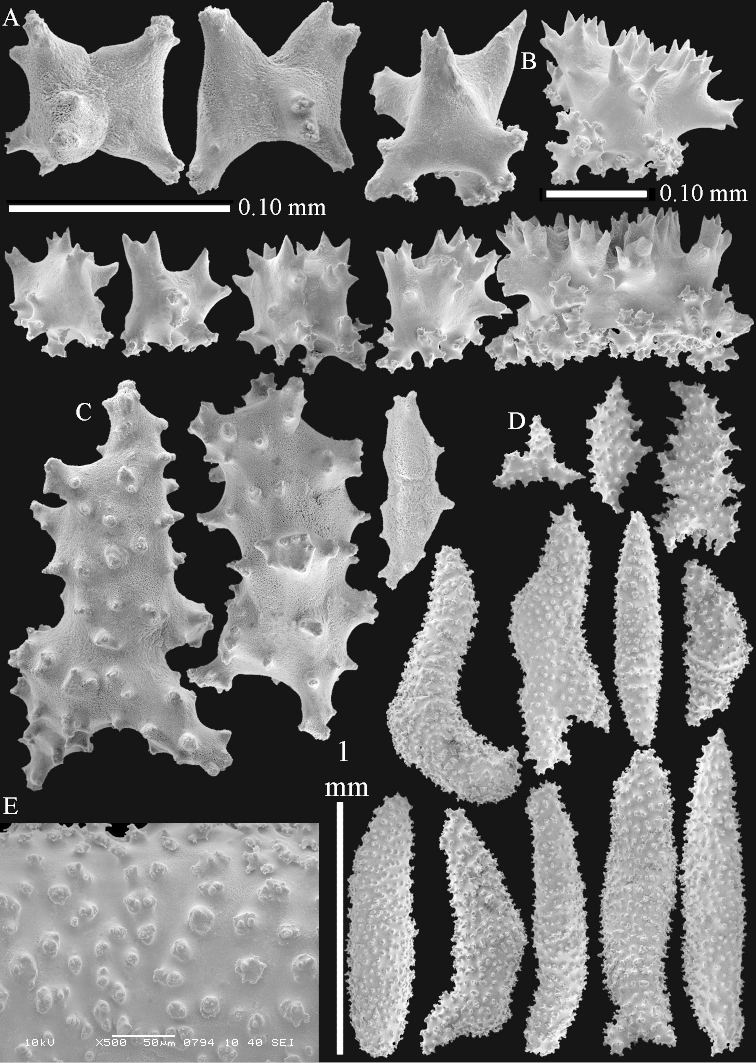
*Litophyton lanternarium* (Verseveldt, 1973), holotype RMNH Coel. 8052. **A–B** sclerites of surface layer base of stalk **C–D** spindles interior base of stalk **E** tubercles on spindle. Scale at **B** also applies to **C**.

**Figure 45.**
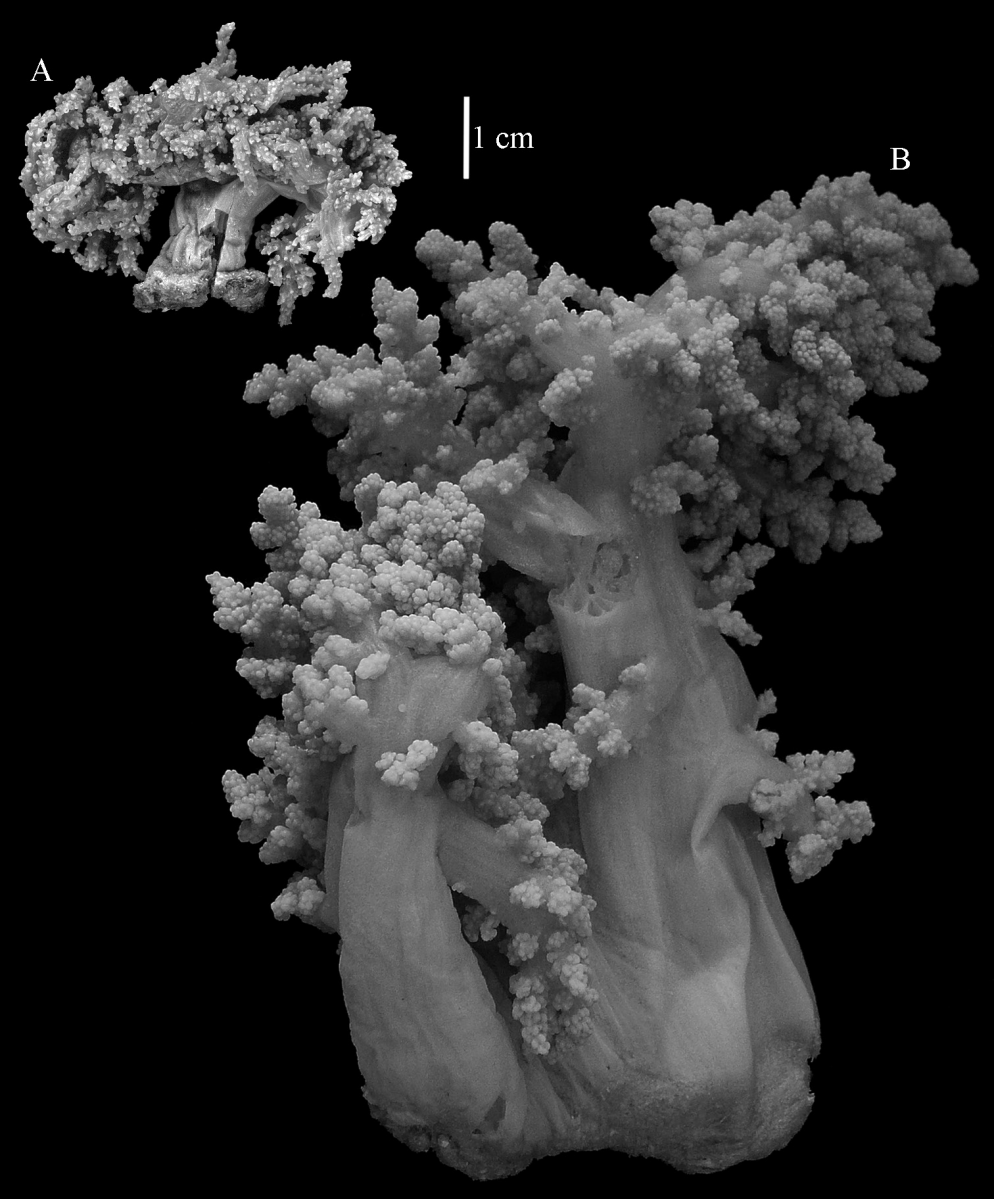
*Litophyton maldivensis* (Hickson, 1905). **A** holotype BMNH 1962.7.20.124 **B** ZMTAU Co 26249.

**Figure 46.**
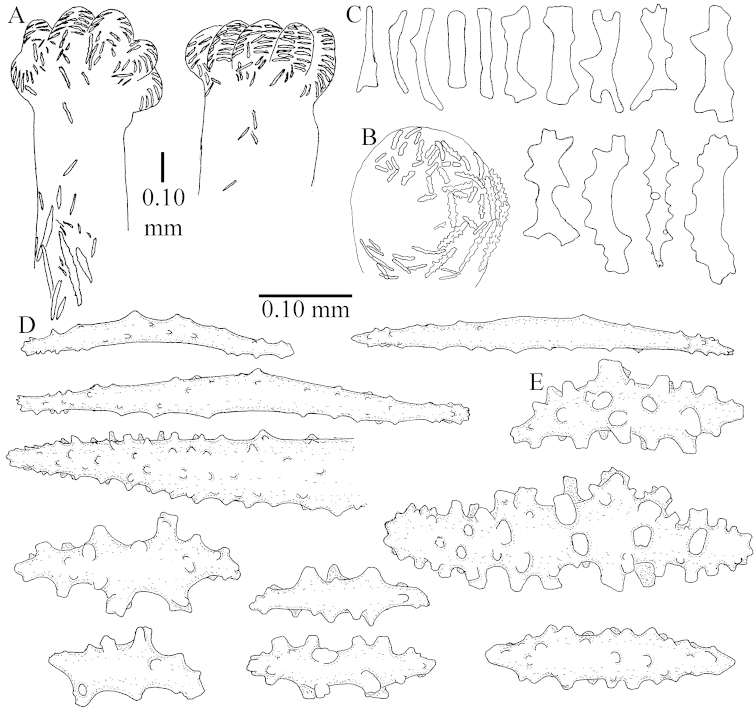
*Litophyton maldivensis* (Hickson, 1905). **A, C–E** holotype BMNH 1962.7.20.124 **B** ZMTAU Co 26249 **A–B** polyp armature **C** polyp rodlets **D** spindles of lobe **E** sclerites surface layer top of stalk. Scale at **A** applies to **A–B**.

**Figure 47.**
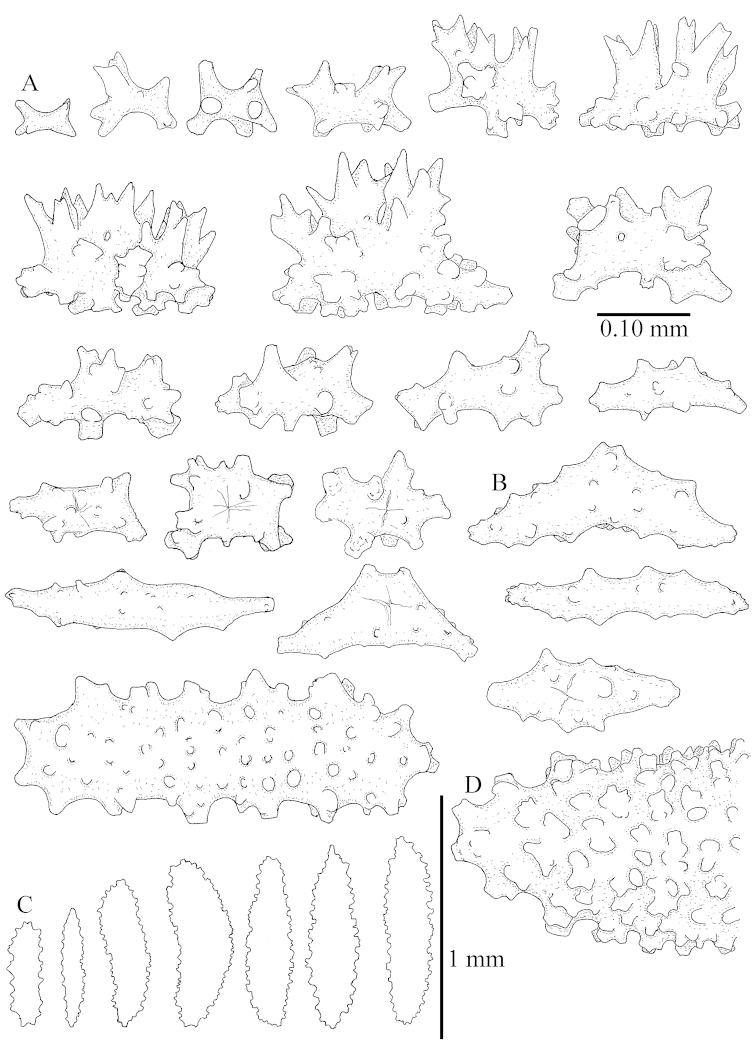
*Litophyton maldivensis* (Hickson, 1905) holotype BMNH 1962.7.20.124. **A** sclerites surface (bracket after Hickson, 1905) layer base of stalk **B–C** spindles interior base of stalk **D** tubercles on spindle.

**Figure 48.**
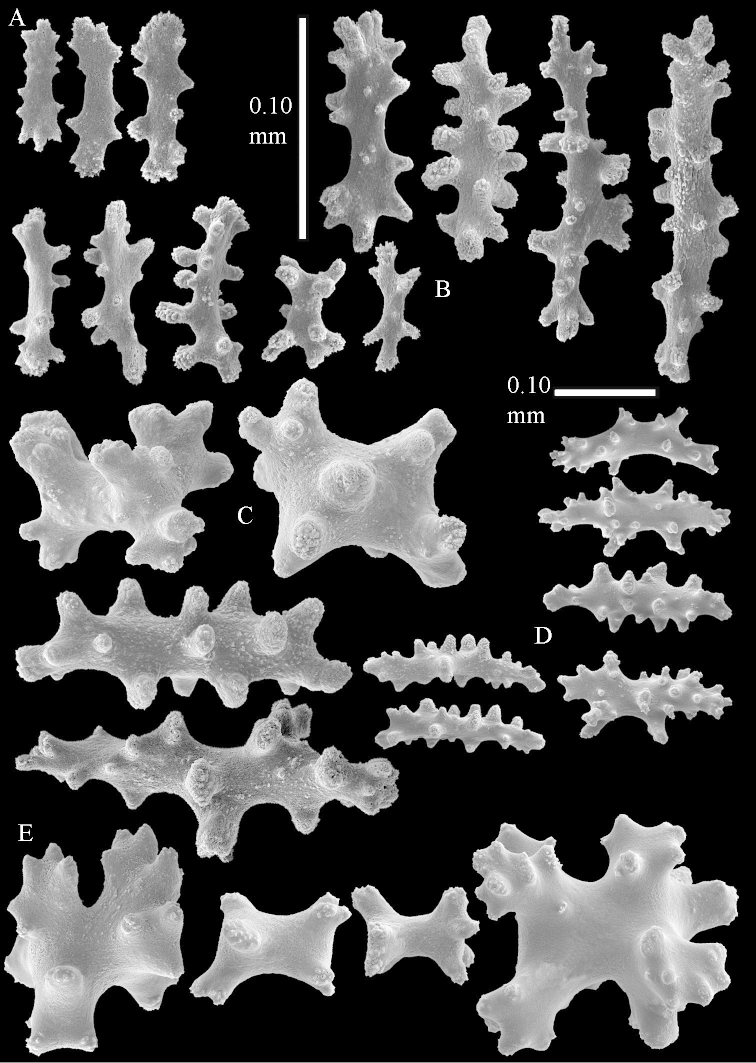
*Litophyton maldivensis* (Hickson, 1905) ZMTAU Co 26249. **A** tentacle rodlets **B** polyp body spindles **C–D** sclerites surface layer top of stalk **E** sclerites of surface layer base of stalk. Scale at **D** only applies to **D**.

**Figure 49.**
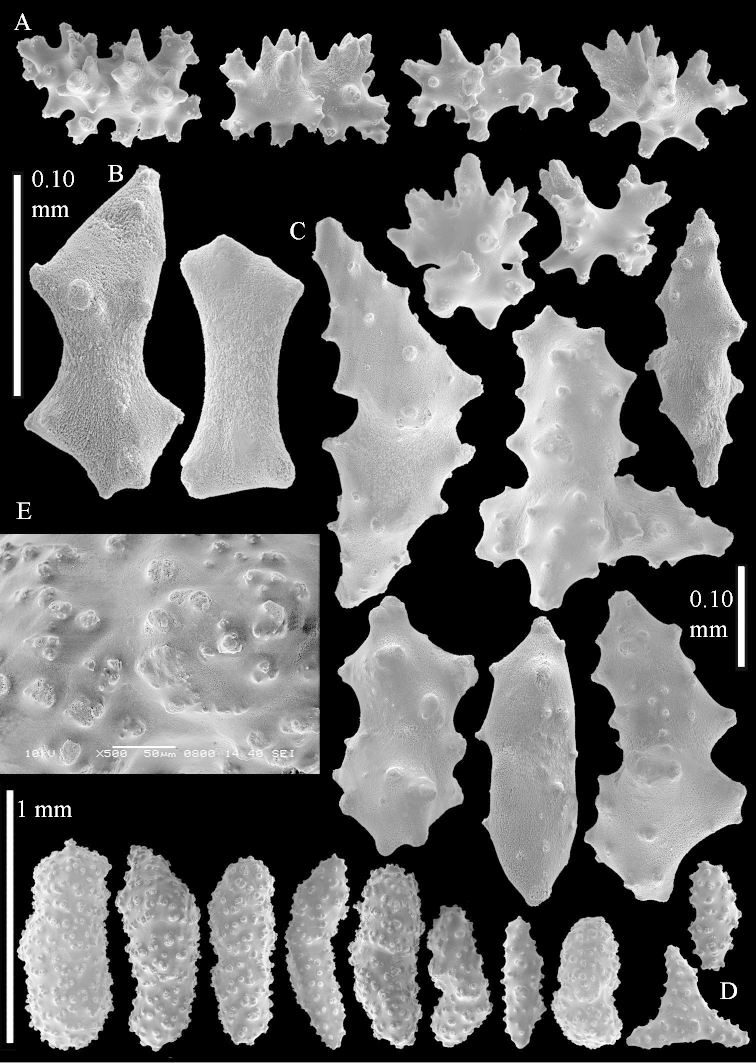
*Litophyton maldivensis* (Hickson, 1905) ZMTAU Co 26249. **A** sclerites surface layer base of stalk **B–D** spindles interior base of stalk **E** tubercles on spindle. Scale at **C** also applies to **A**.

**Figure 50.**
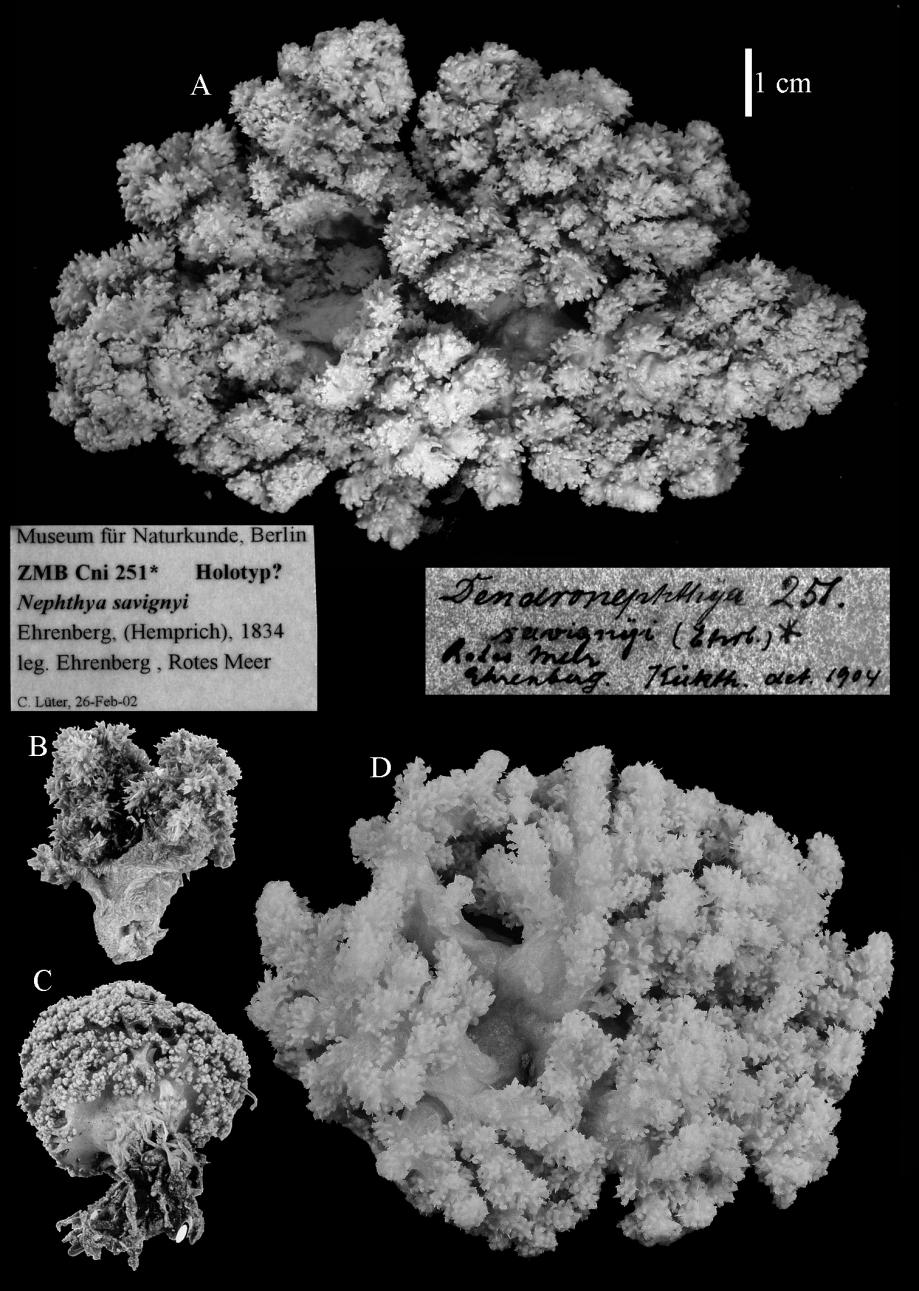
Litophyton ?savignyi (Ehrenberg, 1834). **A** Probable holotype ZMB Cni 251 **B–C** NHMW 2407 **B** *Litophyton savignyi* **C** *Dendronephthya* spec. **D** UUZM 417, type *Nephthya jaegerskioeldi*.

**Figure 51.**
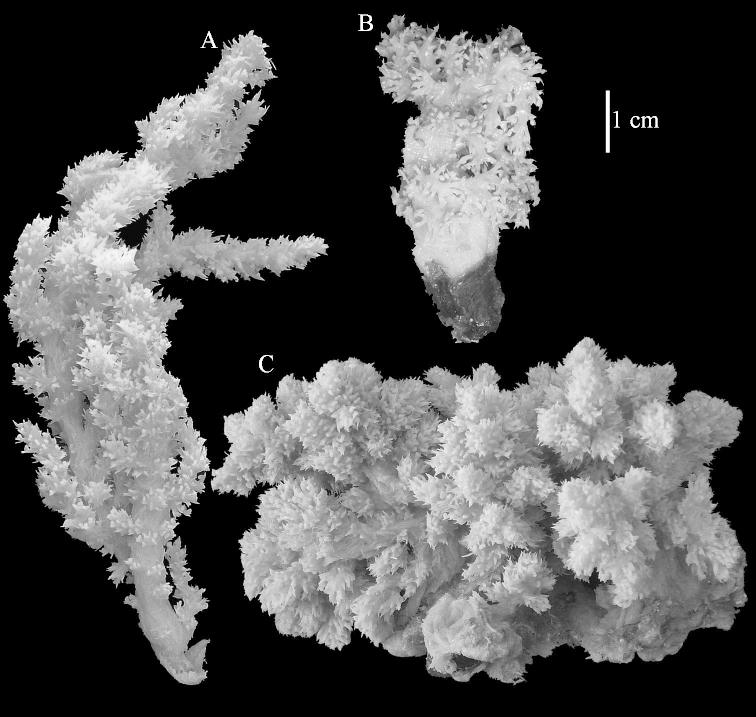
Litophyton ?savignyi (Ehrenberg, 1834). **A** ZMTAU Co 25829 **B** ZMTAU Co 34067 **C** ZMTAU Co 26245.

**Figure 52.**
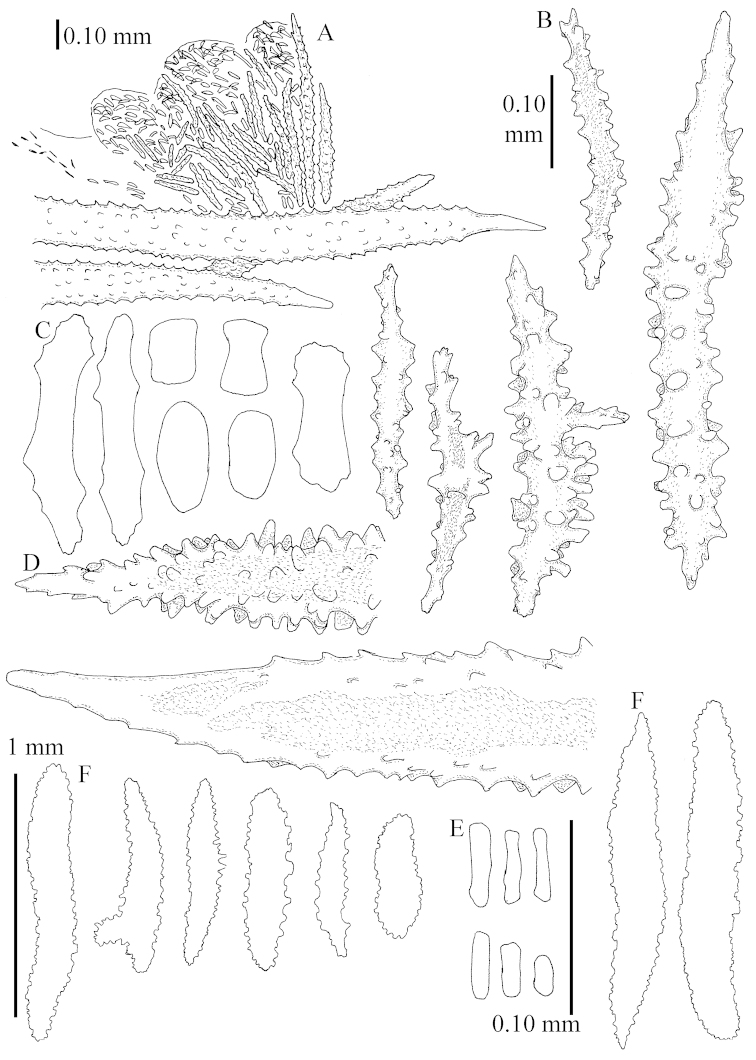
Litophyton ?savignyi (Ehrenberg, 1834), NHMW 2407. **A** lateral view of polyp armature **B** polyp body spindles **C** tentacle rodlets **D** supporting bundle spindles (partly) **E** rodlets from polyp stalk **F** spindles of interior base of stalk. Scale at **B** also applies to **D**, scale at **E** also to **C**.

**Figure 53.**
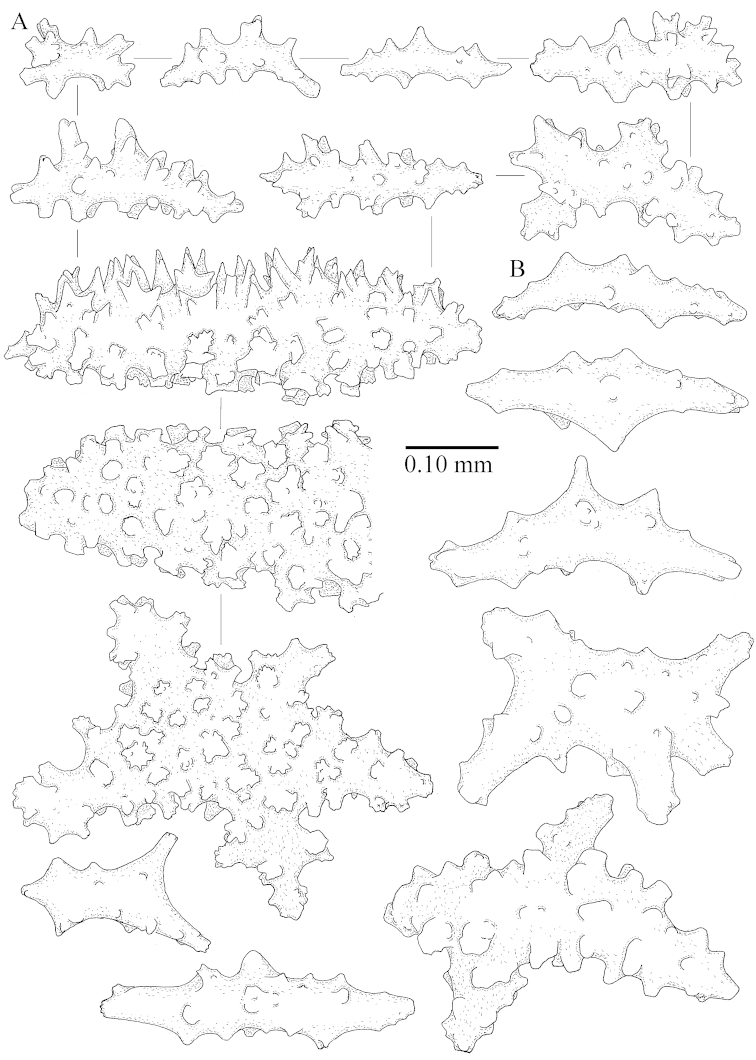
Litophyton ?savignyi (Ehrenberg, 1834), NHMW 2407. **A** sclerites surface layer base of stalk **B** spindles interior base of stalk.

**Figure 54.**
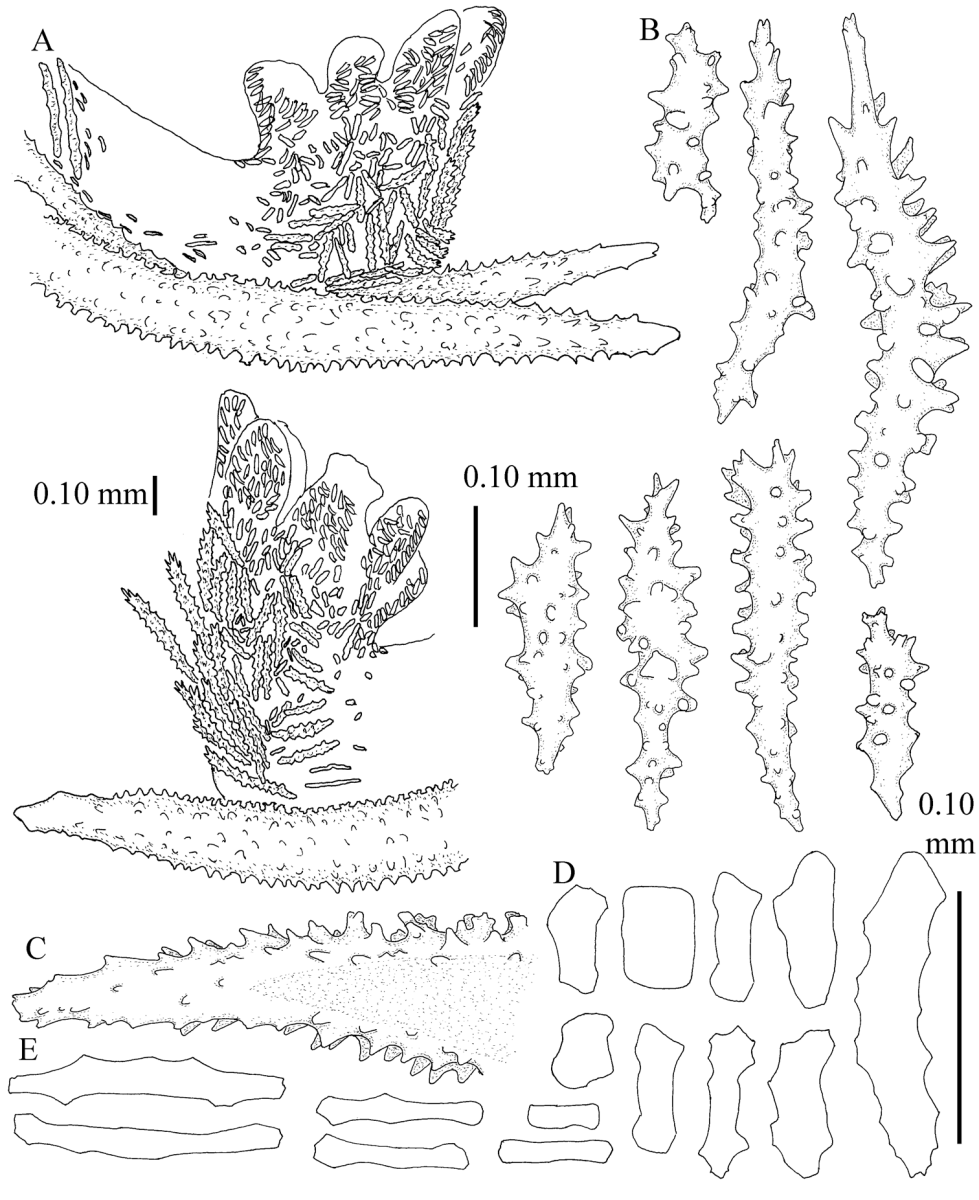
Litophyton ?savignyi (Ehrenberg, 1834), UUZM 417, type of *Nephthya jaegerskioeldi*. **A** lateral views of polyp armature **B** polyp body spindles **C** supporting bundle spindle (partly) **D** tentacle rodlets **E** rodlets from polyp stalk. Scale at **B** also applies to **C**, scale at **D** also to **E**.

**Figure 55.**
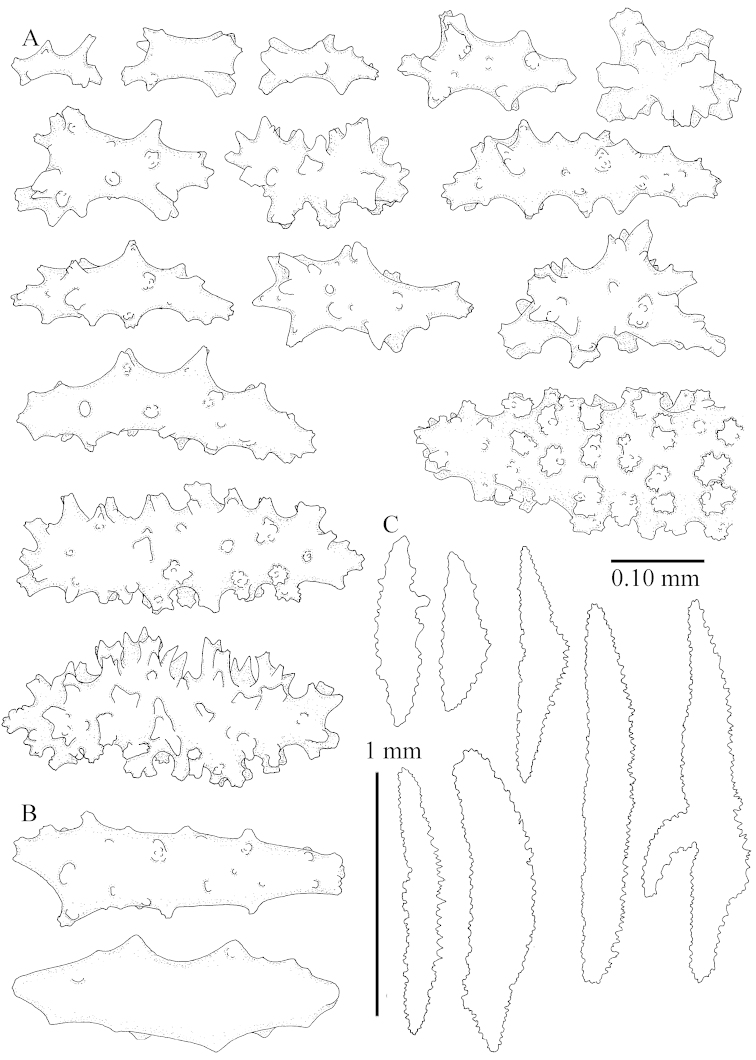
Litophyton ?savignyi (Ehrenberg, 1834), UUZM 417, type of *Nephthya jaegerskioeldi*. **A** sclerites surface layer base of stalk **B–C** spindles interior base of stalk. Scale at **C** only applies to **C**.

**Figure 56.**
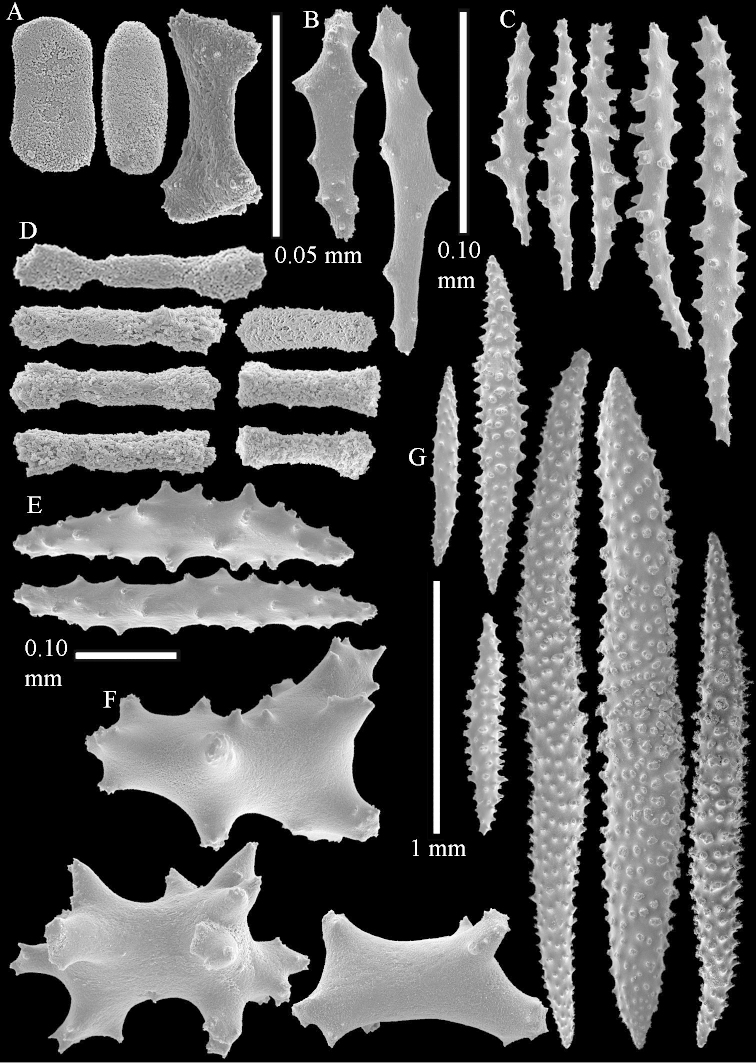
Litophyton ?savignyi (Ehrenberg, 1834), ZMTAU 26245. **A** tentacle rodlets **B–C** polyp body spindles **D** rodlets from polyp stalk **E–G** sclerites surface layer top of stalk. Scale at **B** also applies to **F**, scale at **E** also to **C**, scale at **A** also to **D**.

**Figure 57.**
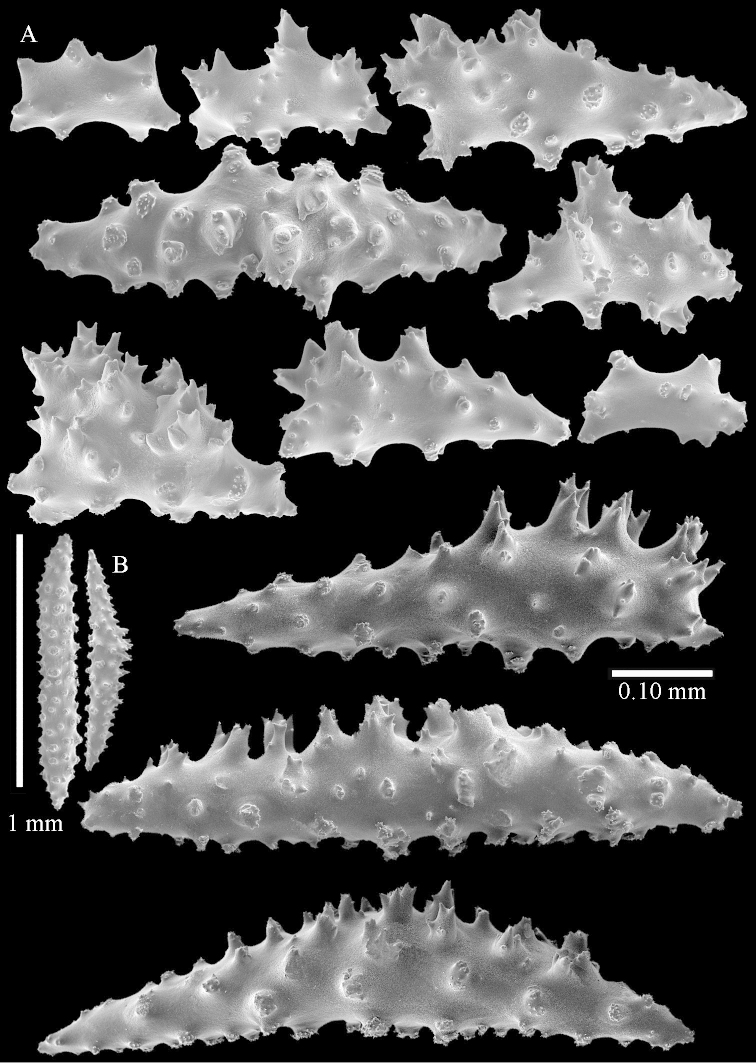
Litophyton ?savignyi (Ehrenberg, 1834), ZMTAU 26245. **A–B** sclerites surface layer base of stalk.

**Figure 58.**
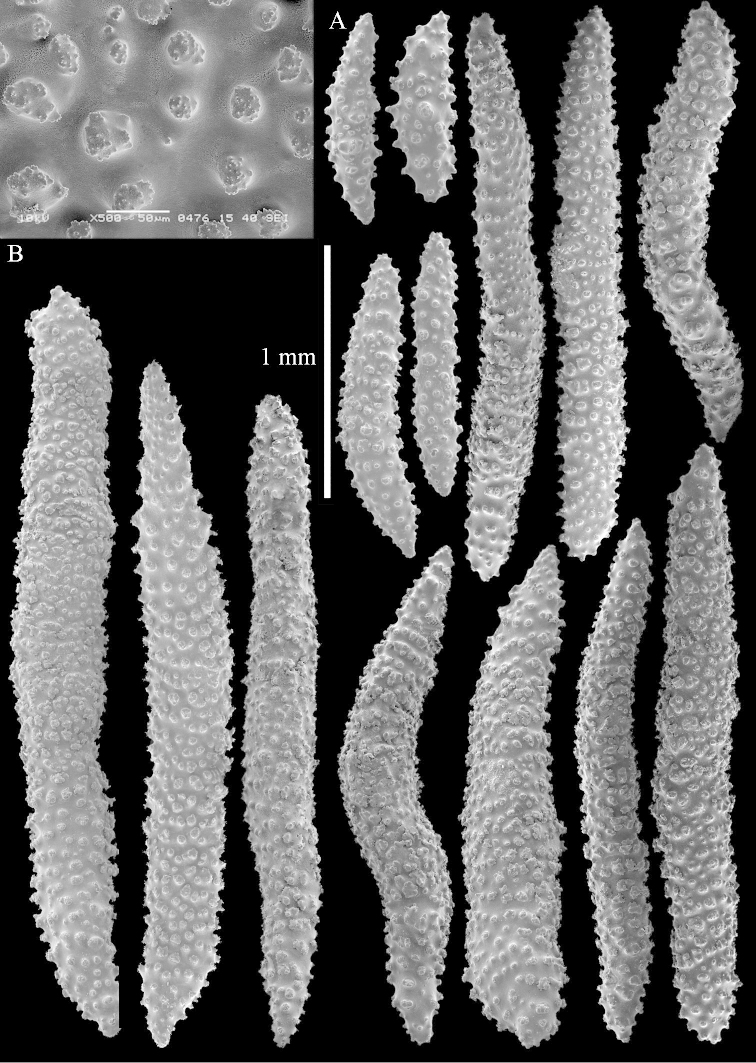
Litophyton ?savignyi (Ehrenberg, 1834), ZMTAU 26245. **A** spindles interior base of stalk **B** tubercles on spindle.

**Figure 59.**
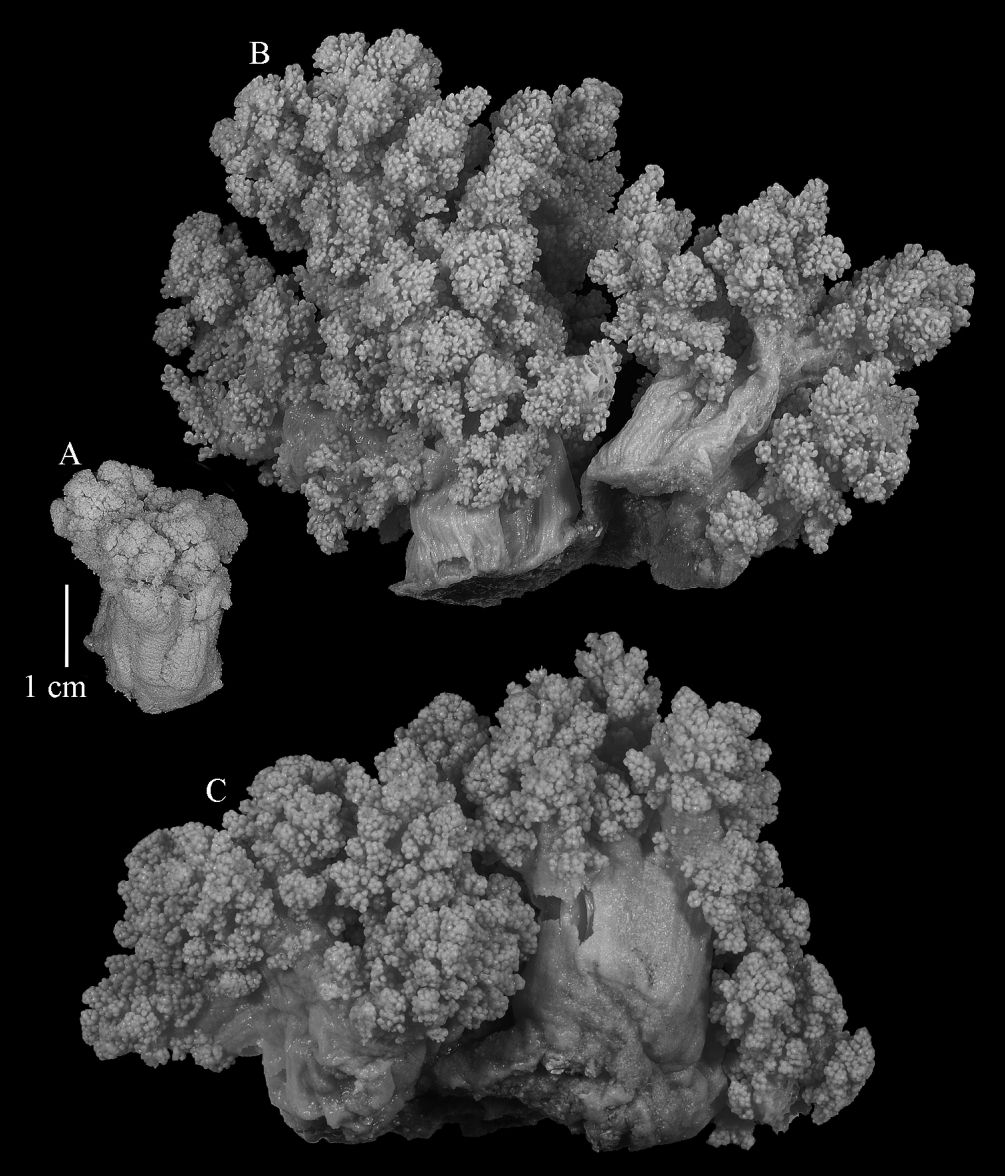
*Litophyton simulatum* (Verseveldt, 1970). **A** ZMB 6838, syntype of *Litophyton striatum* (Kükenthal, 1903) **B** ZMTAU Co 25874 **C** ZMTAU Co26201.

**Figure 60.**
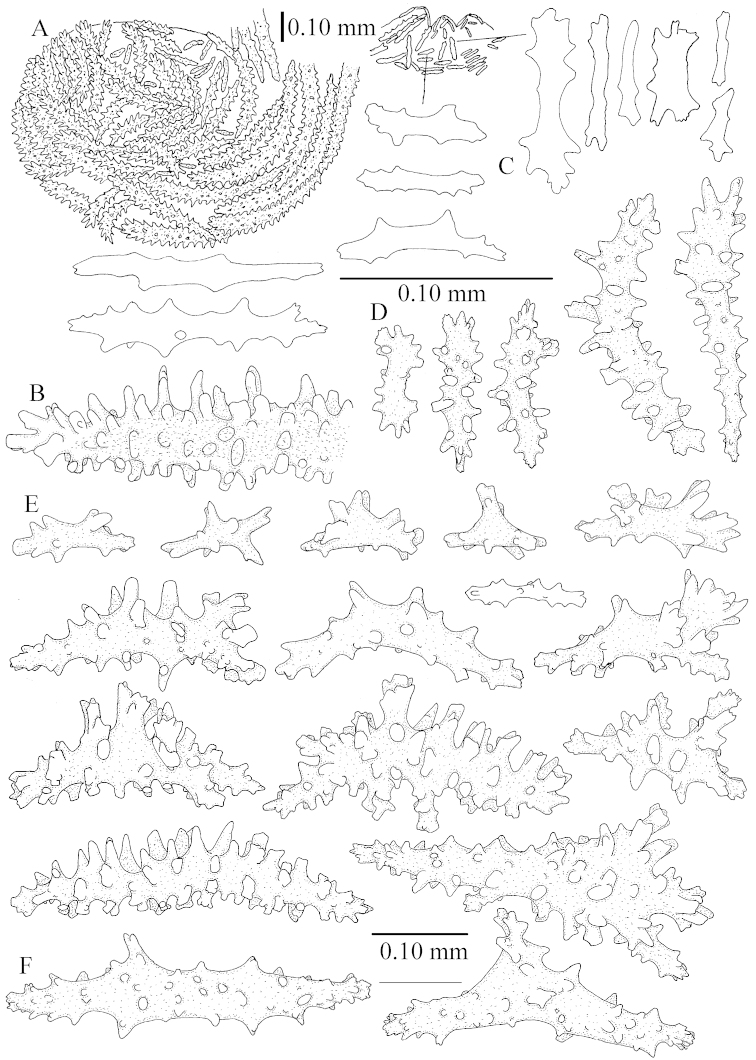
*Litophyton simulatum* (Verseveldt, 1970), ZMB 6838, syntype of *Litophyton striatum* (Kükenthal, 1903) **A** lateral view of polyp armature and adaxial view of part of it **B** supporting bundle sclerite (partly) **C–D** polyp body sclerites **E** sclerites, surface layer top of stalk **F** spindles interior top of stalk. Scale at **F** also applies to **B**, **D, E**.

**Figure 61.**
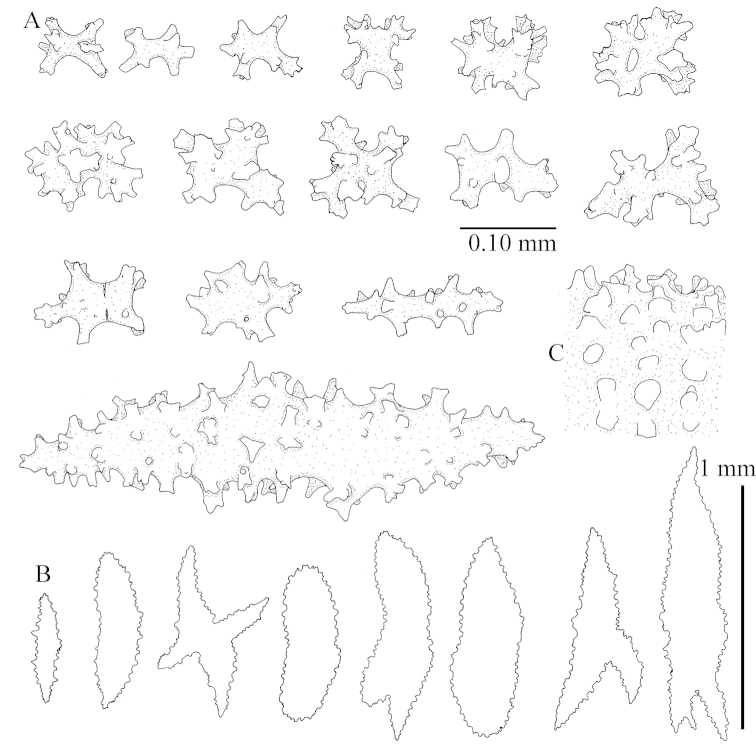
*Litophyton simulatum* (Verseveldt, 1970), ZMB 6838, syntype of *Litophyton striatum* (Kükenthal, 1903). **A** sclerites surface layer base of stalk **B** spindles interior base of stalk, outlines only **C** tubercles on spindle. Scale at **A** also applies to **C**.

**Figure 62.**
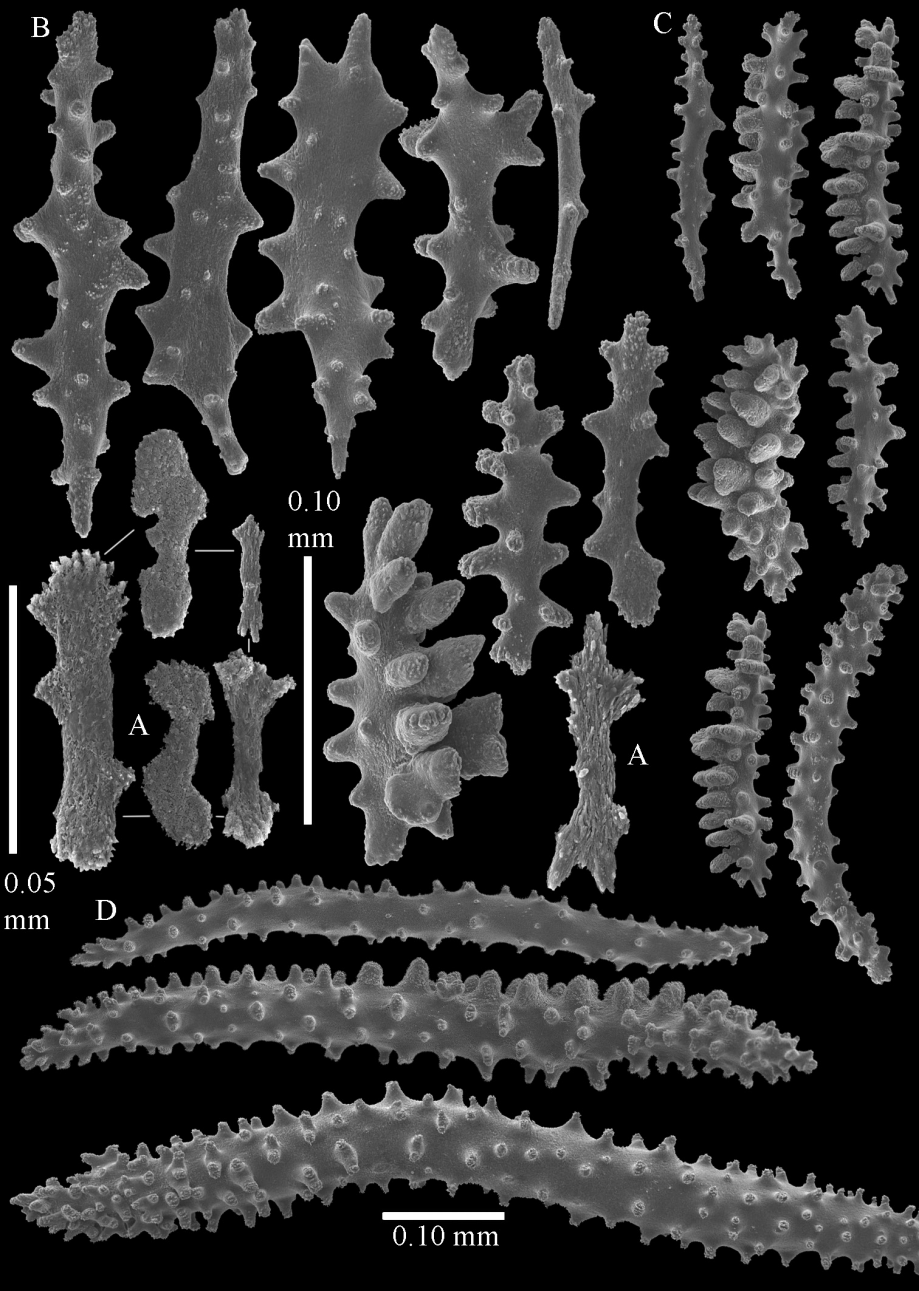
*Litophyton simulatum* (Verseveldt, 1970), ZMTAU Co 25874. **A** tentacle rodlets **B–C** polyp body spindles **D** spindles of supporting bundle. Scale at **D** also applies to **C**.

**Figure 63.**
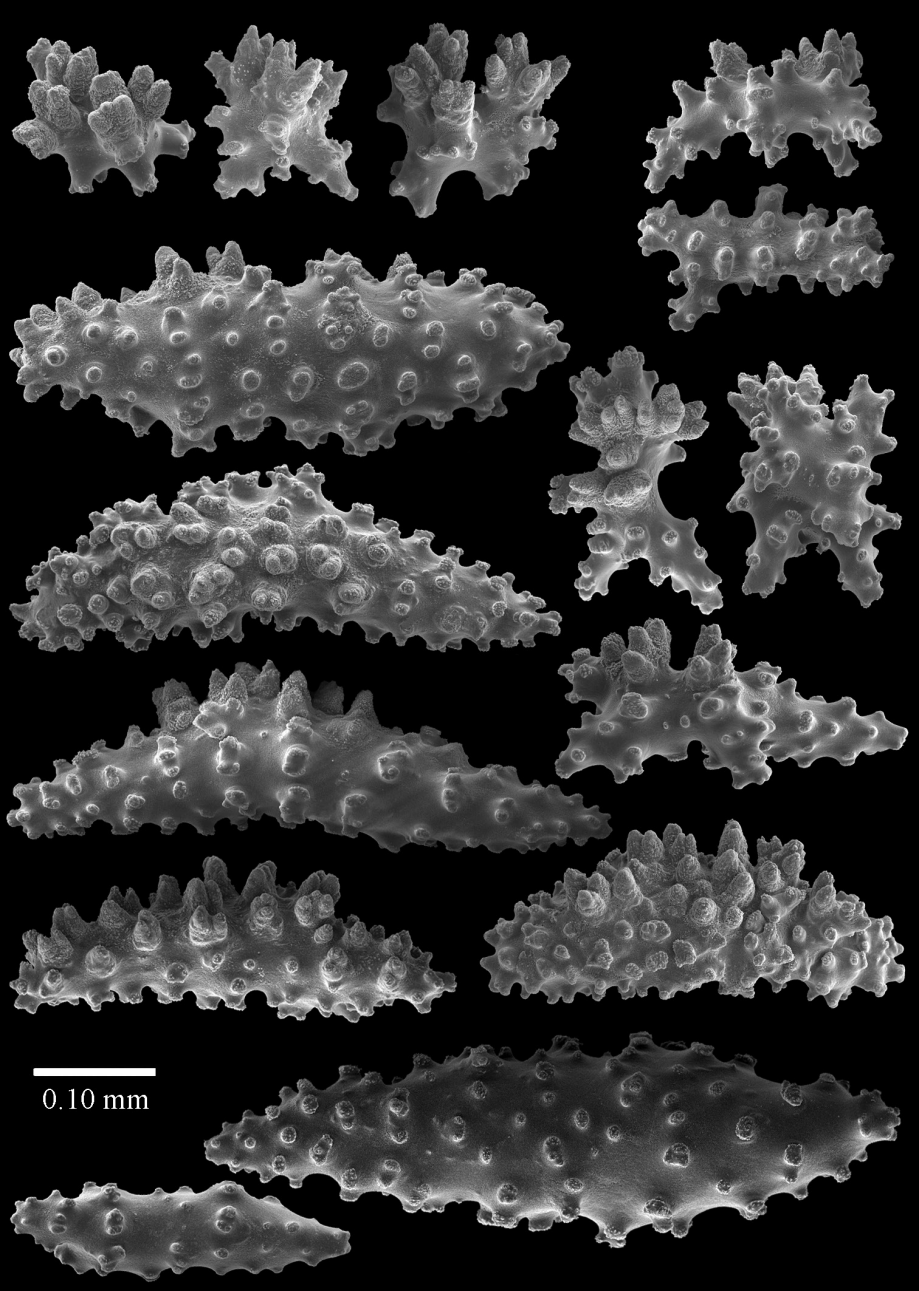
*Litophyton simulatum* (Verseveldt, 1970), ZMTAU Co 25874. Sclerites surface layer top of stalk.

**Figure 64.**
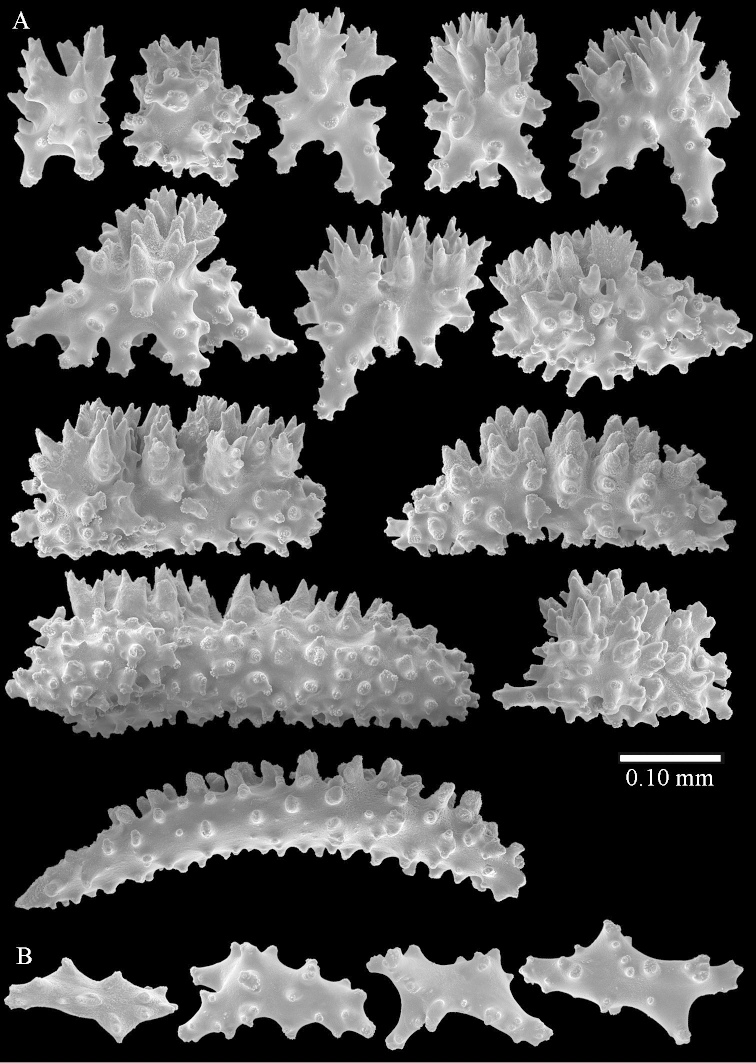
*Litophyton simulatum* (Verseveldt, 1970), ZMTAU Co 25874. **A** sclerites surface layer base of stalk **B** spindles of interior of base of stalk.

**Figure 65.**
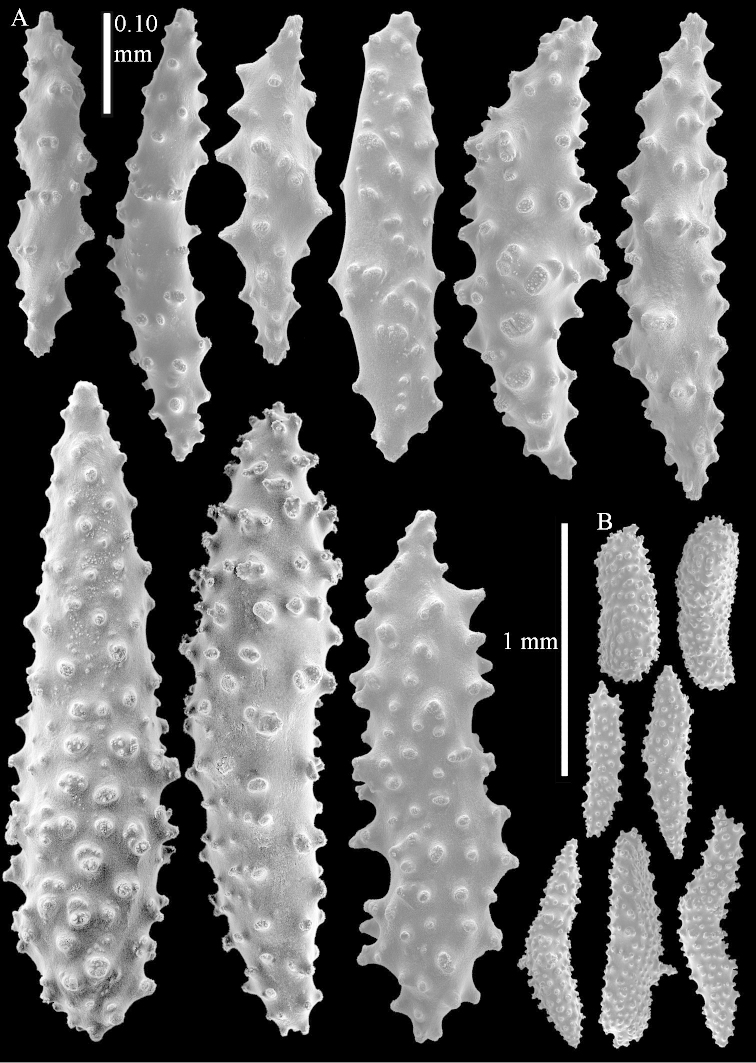
*Litophyton simulatum* (Verseveldt, 1970), ZMTAU Co 25874. **A–B** spindles interior base of stalk.

**Figure 66.**
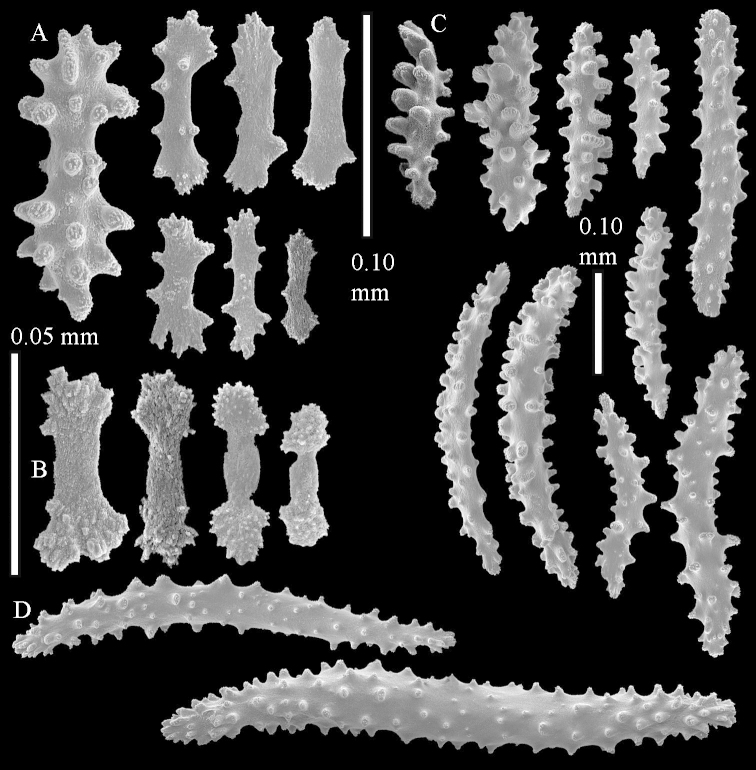
*Litophyton simulatum* (Verseveldt, 1970), ZMTAU Co 26201. **A–B** tentacle rodlets **C** polyp body spindles **D** spindles of supporting bundle. Scale at **C** also applies to **D**.

**Figure 67.**
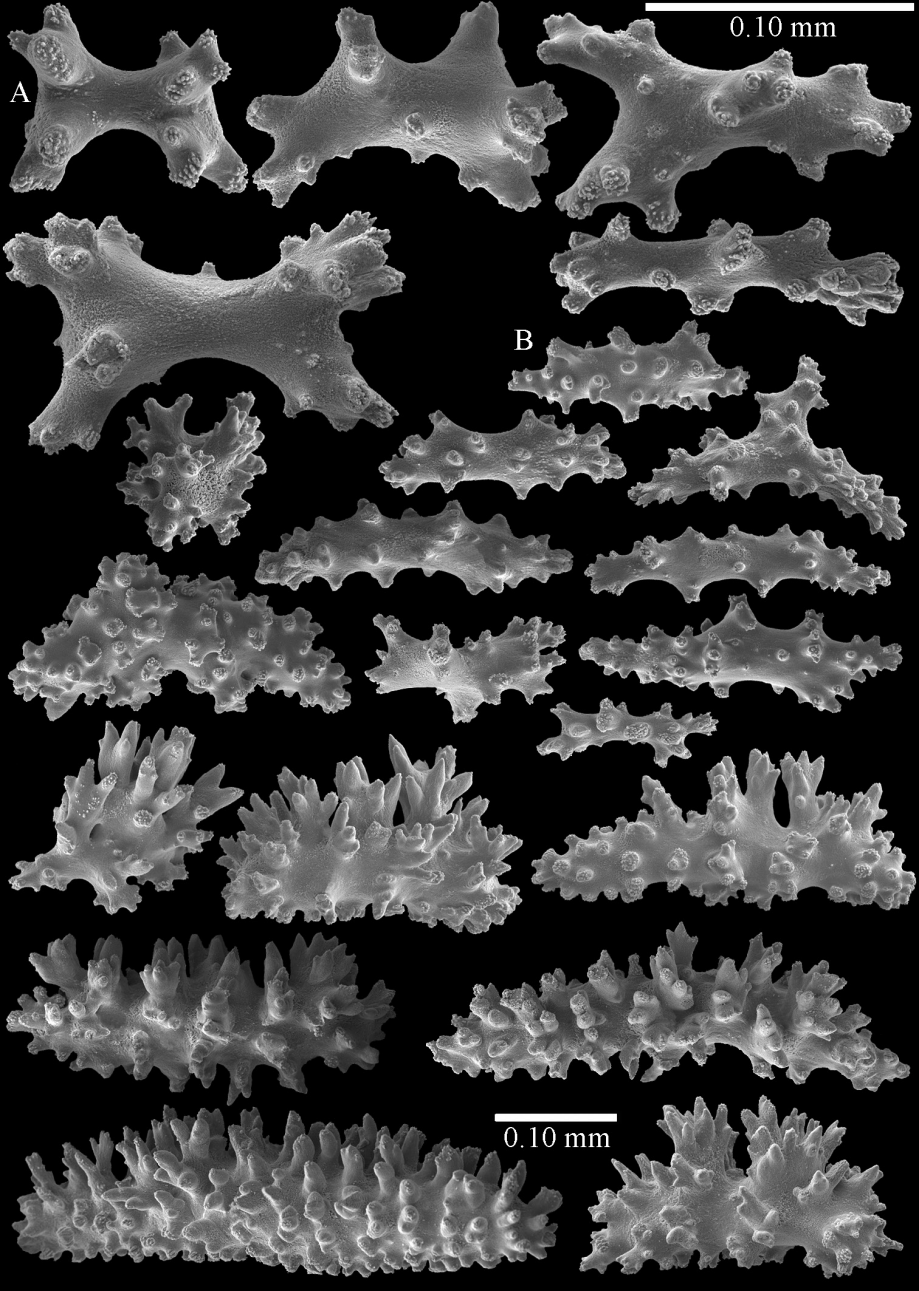
*Litophyton simulatum* (Verseveldt, 1970), ZMTAU Co 26201. **A–B** sclerites surface layer top of stalk.

**Figure 68.**
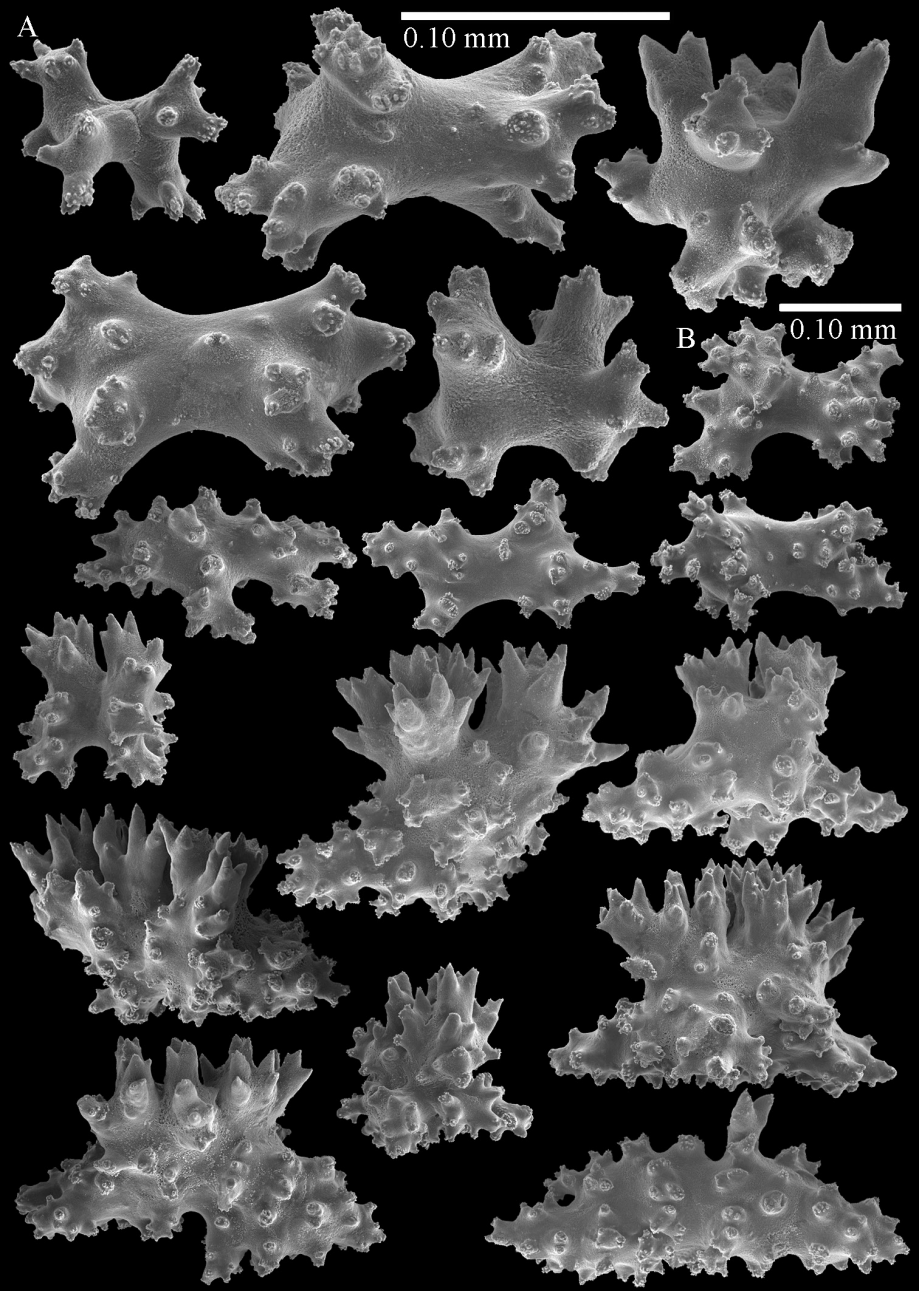
*Litophyton simulatum* (Verseveldt, 1970), ZMTAU Co 26201 **A–B** sclerites surface layer base of stalk.

**Figure 69.**
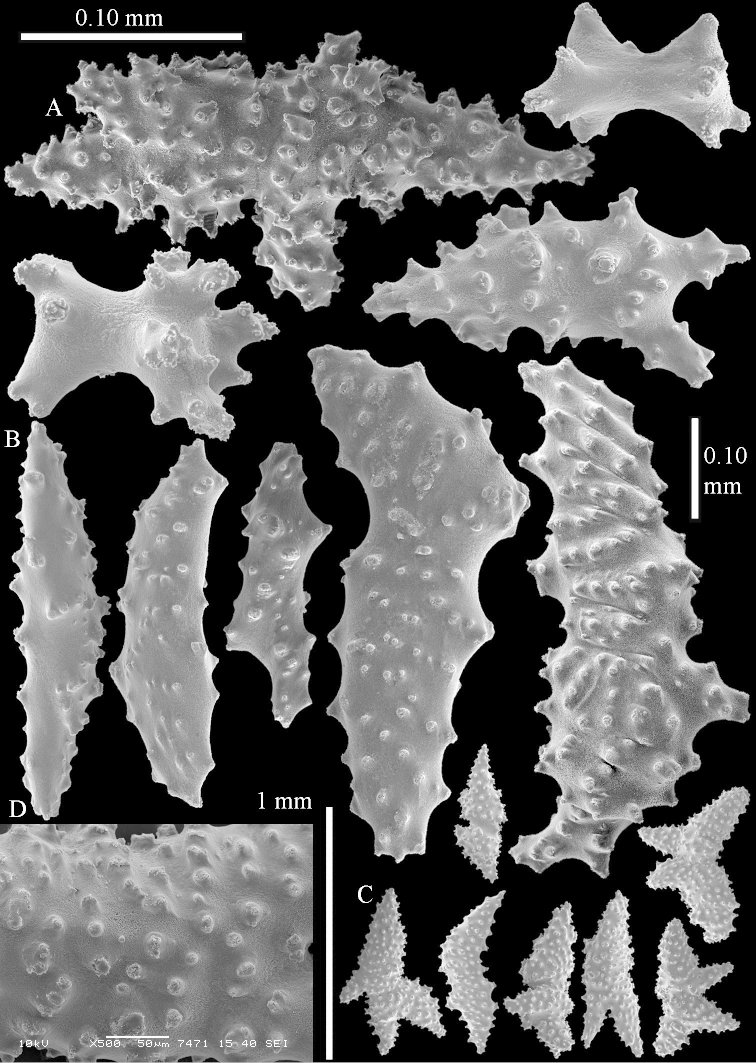
*Litophyton simulatum* (Verseveldt, 1970), ZMTAU Co 26201. **A** sclerites surface layer base of stalk **B–C** spindles of interior base of stalk **D** tubercles on spindle.

**Figure 70.**
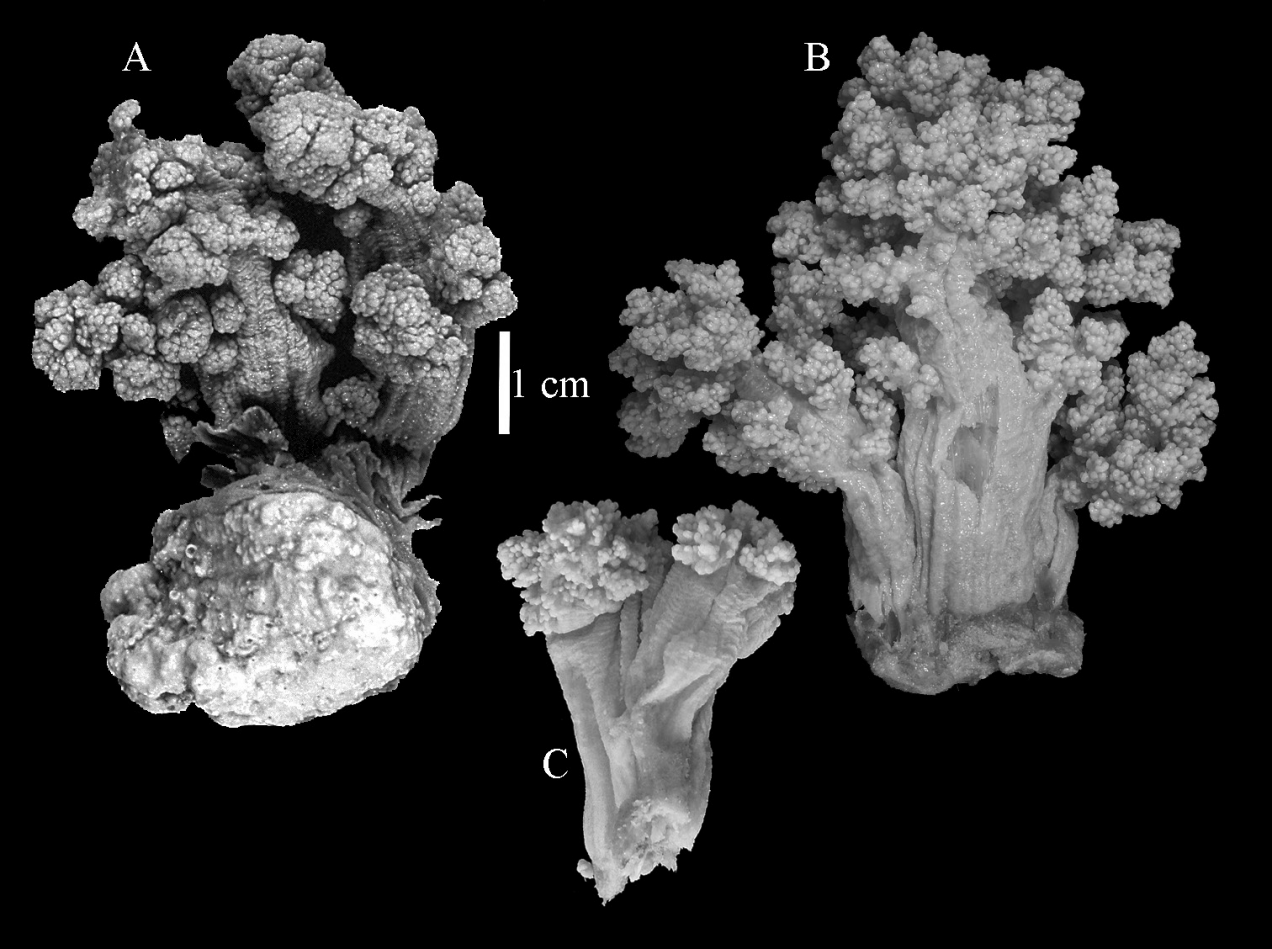
*Litophyton striatum* (Kükenthal, 1903). **A** syntype SMF 1279 **B** ZMTAU Co 25851 **C** ZMTAU Co 26216.

**Figure 71.**
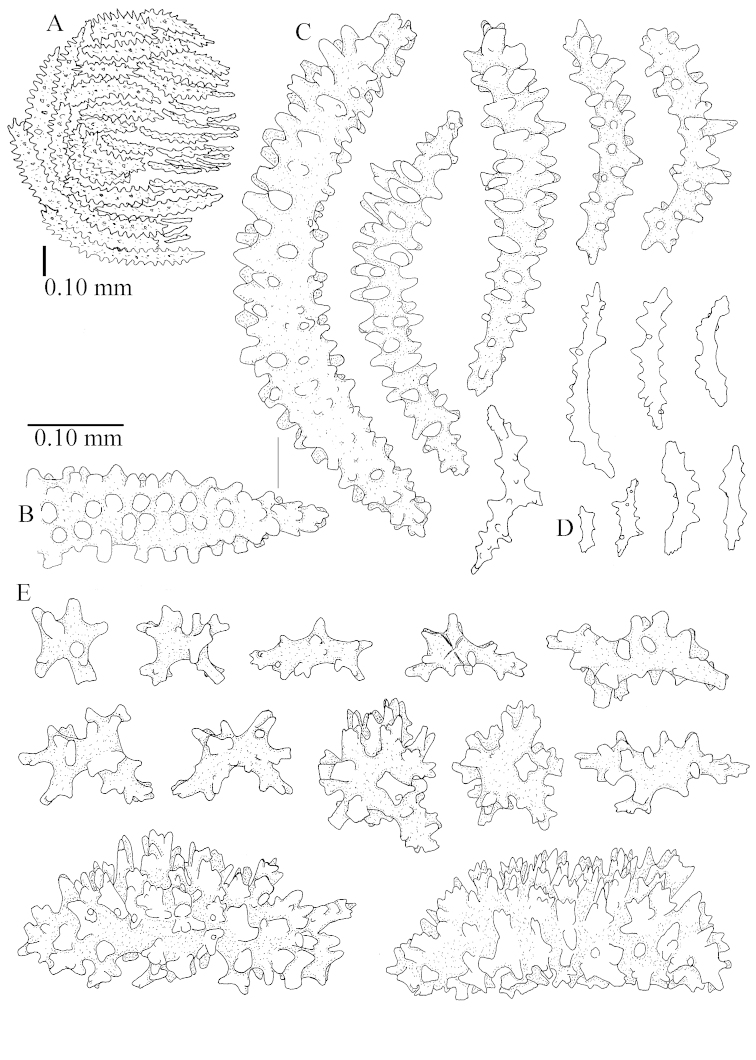
*Litophyton striatum* (Kükenthal, 1903), syntype SMF 1279. **A** lateral view of polyp armature **B** supporting bundle sclerite (partly) **C** polyp body sclerites **D** tentacular rodlets **E** sclerites surface layer top of stalk. Scale at **A** only applies to **A**.

**Figure 72.**
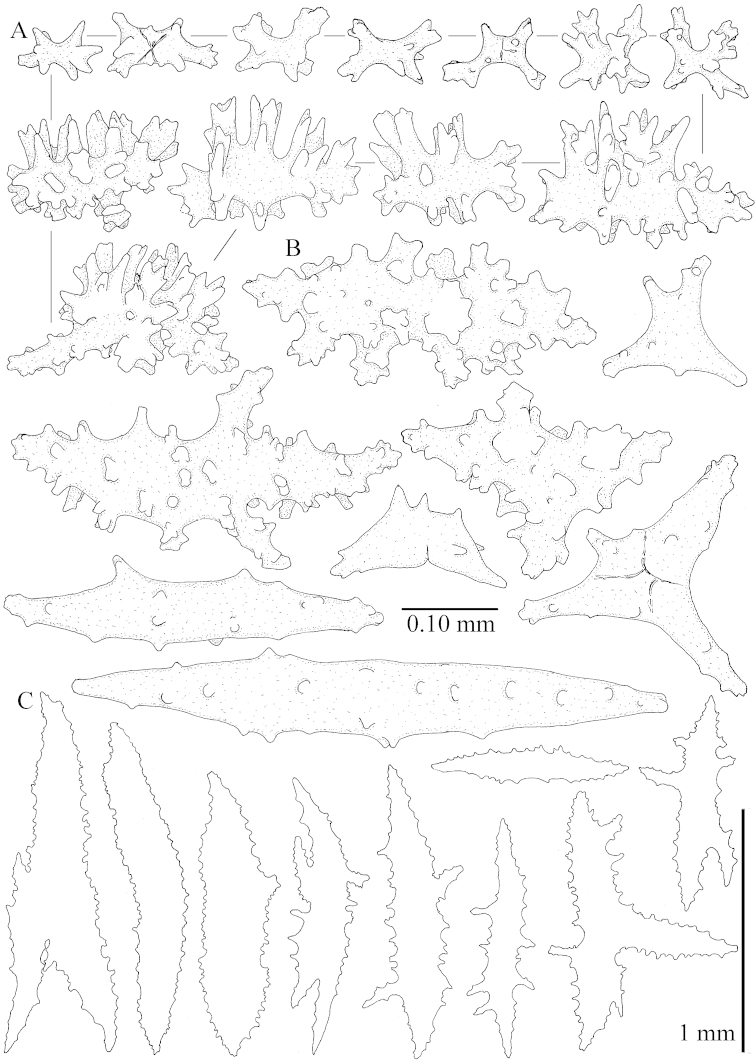
*Litophyton striatum* (Kükenthal, 1903), syntype SMF 1279. **A** sclerites surface layer base of stalk **B–C** spindles interior base of stalk **C** outlines only. Scale at **C** only applies to **C**.

**Figure 73.**
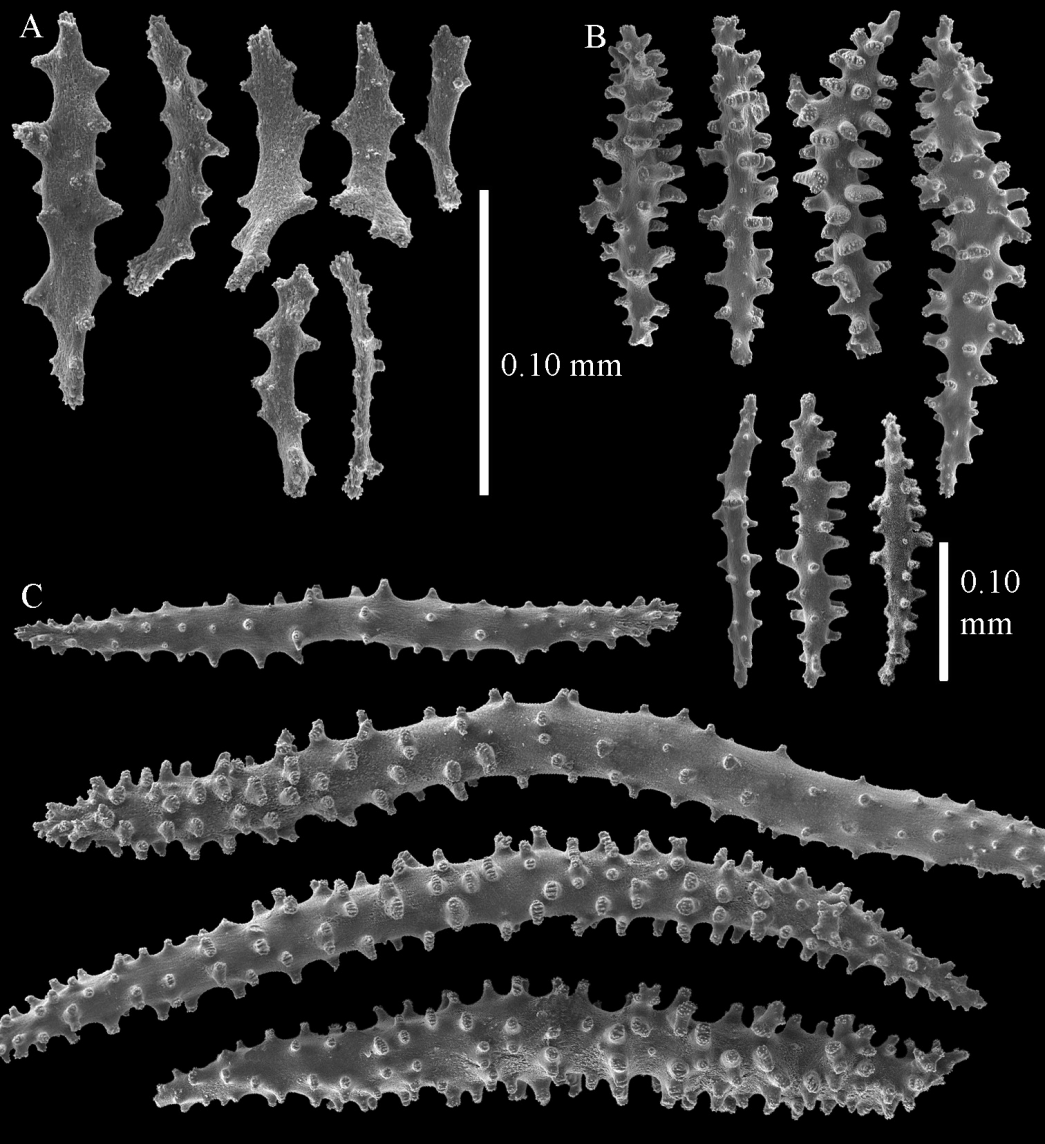
*Litophyton striatum* (Kükenthal, 1903), ZMTAU Co 25851. **A** tentacle rodlets **B** polyp body spindles **C** spindles of supporting bundle. Scale at **B** also applies to **C**.

**Figure 74.**
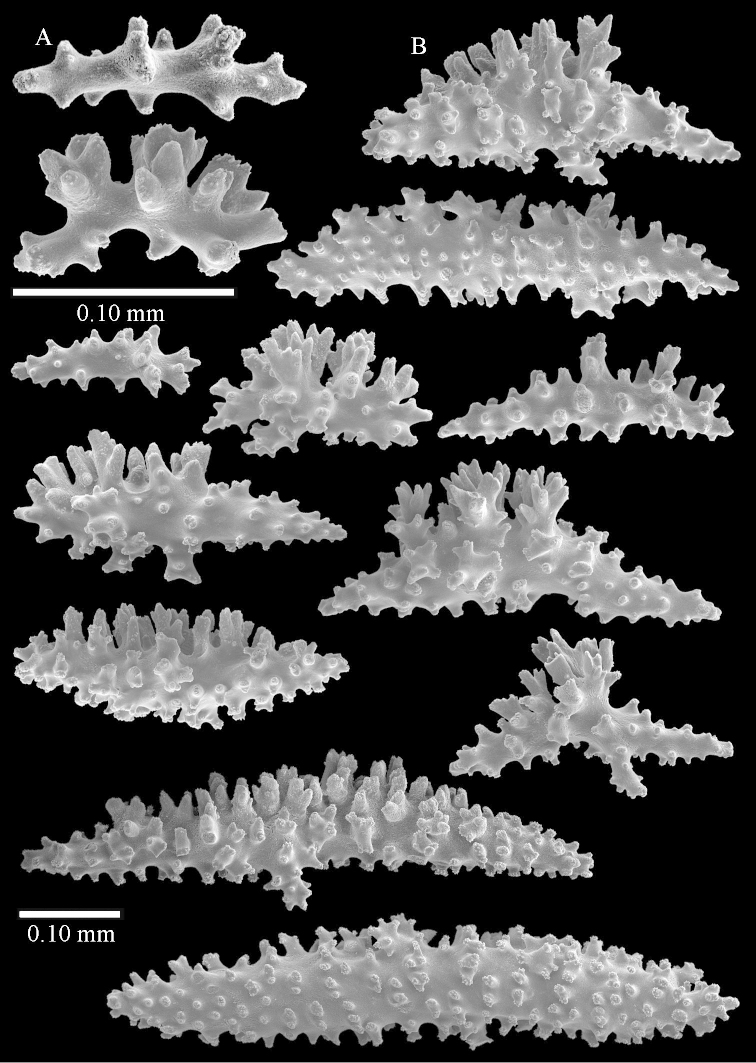
*Litophyton striatum* (Kükenthal, 1903), ZMTAU Co 25851. **A–B** sclerites surface layer top of stalk.

**Figure 75.**
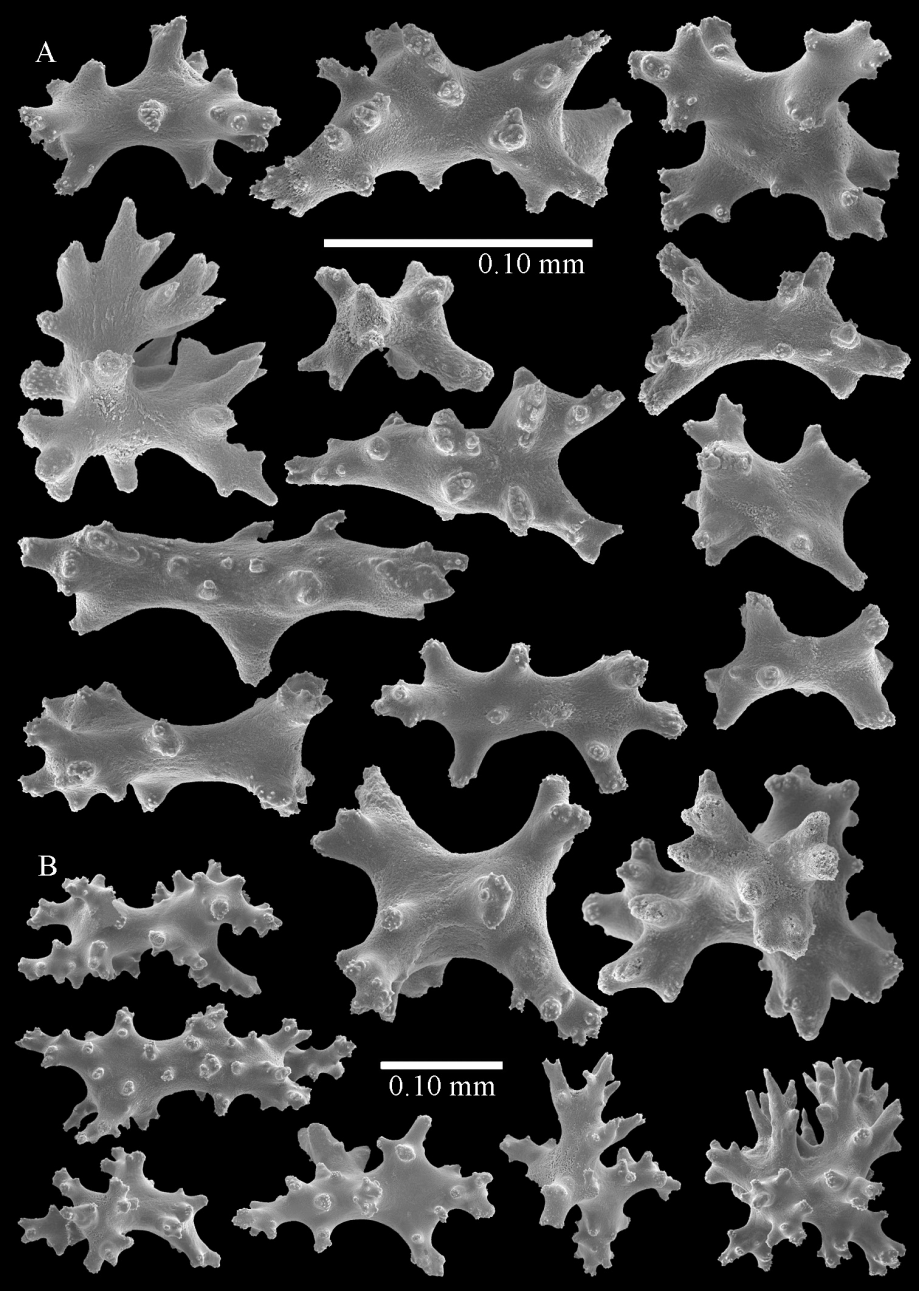
*Litophyton striatum* (Kükenthal, 1903), ZMTAU Co 25851. **A–B** sclerites surface layer base of stalk.

**Figure 76.**
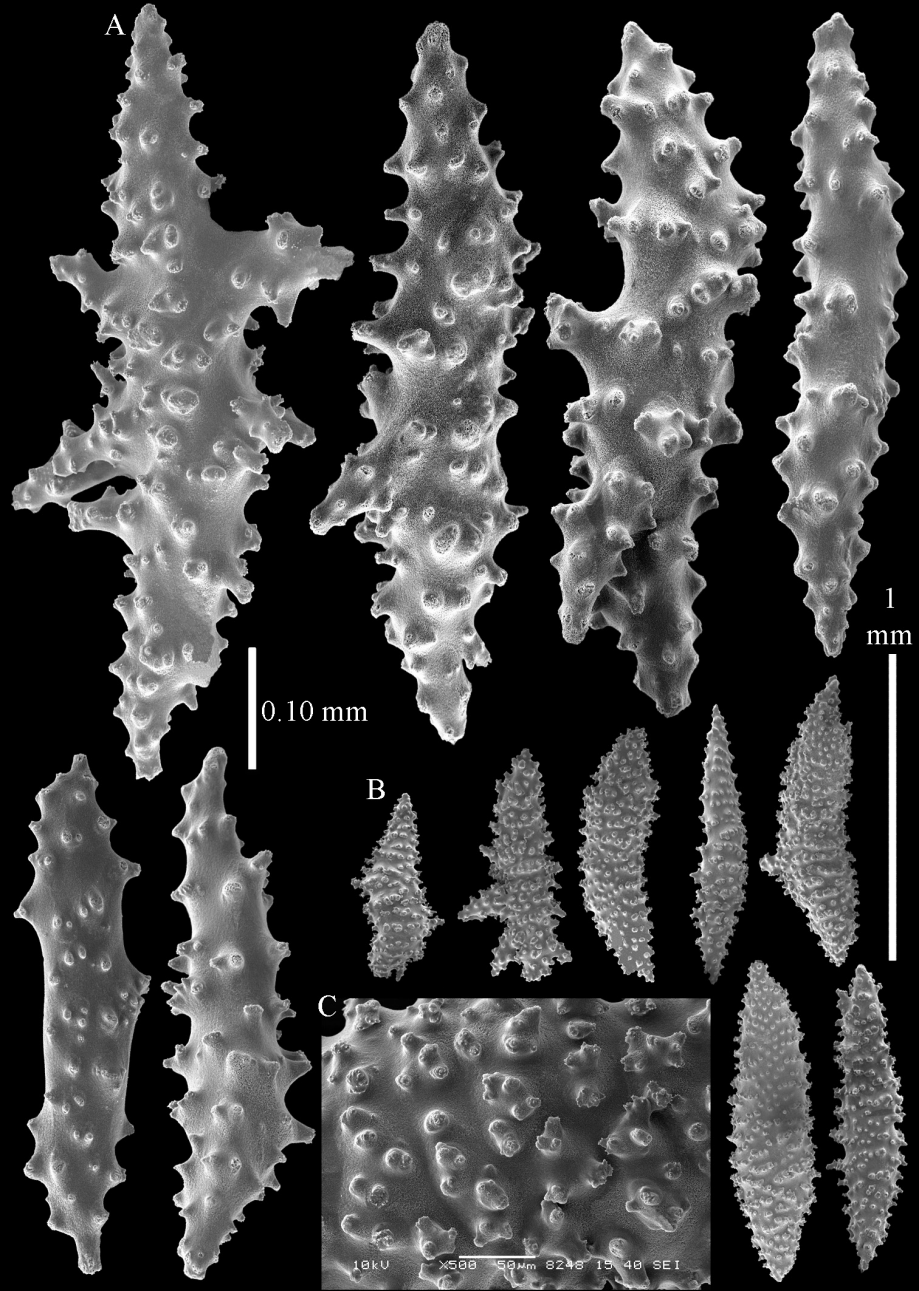
*Litophyton striatum* (Kükenthal, 1903), ZMTAU Co 25851. **A–B** spindles interior base of stalk **C** tubercles on spindle.

**Figure 77.**
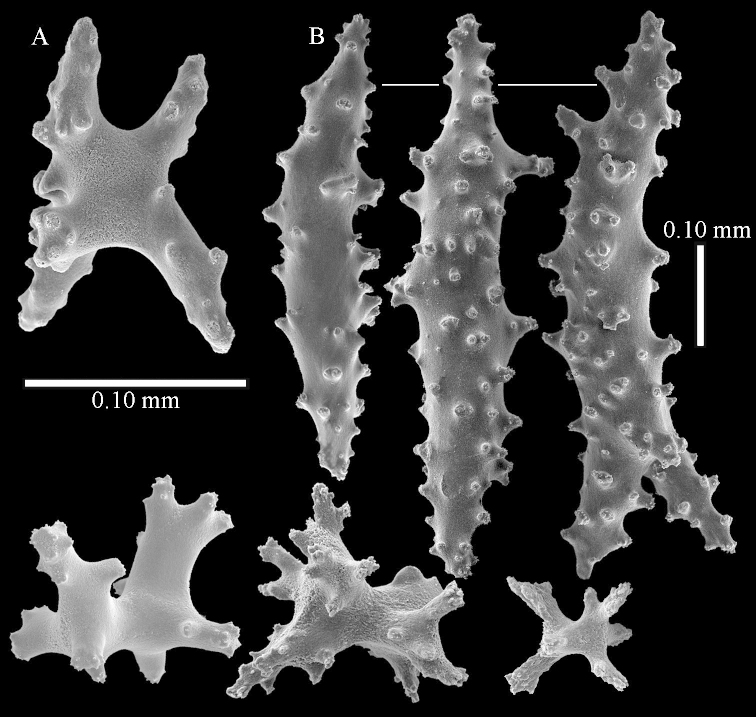
*Litophyton striatum* (Kükenthal, 1903), ZMTAU Co 26216. **A** sclerites surface layer base of stalk **B** spindles interior stalk.

**Figure 78.**
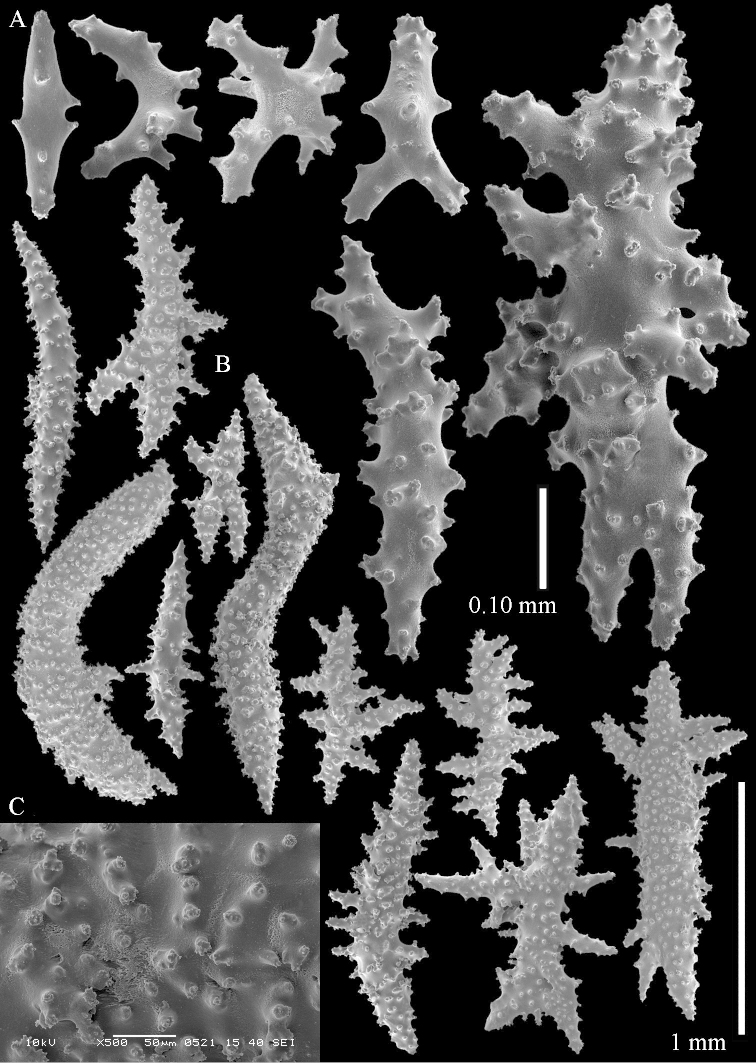
*Litophyton striatum* (Kükenthal, 1903), ZMTAU Co 26216. **A–B** spindles interior base of stalk **C** tubercles on spindle.

**Figure 79.**
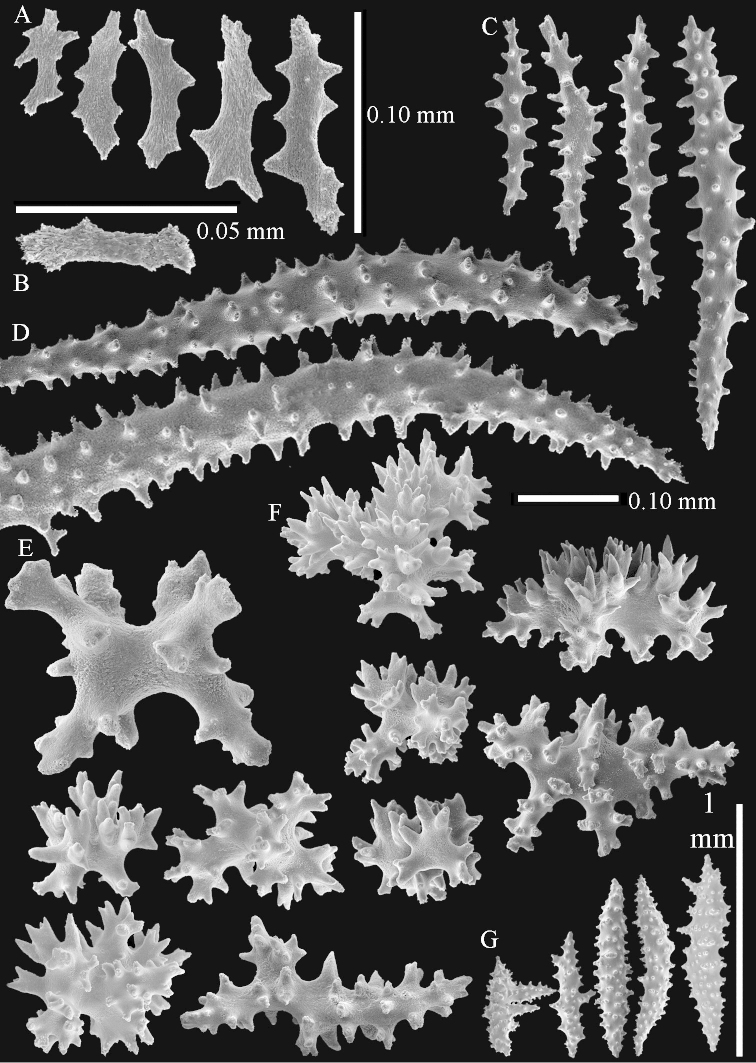
*Litophyton striatum* (Kükenthal, 1903), ZMTAU Co 26203. **A–B** tentacle rodlets **C** polyp body spindles **D** spindles of supporting bundle **E–F** sclerites surface layer base of stalk **G** spindles interior base of stalk. Scale at **D** also applies to **C, F**; scale at **A** also to **E**.

**Figure 80.**
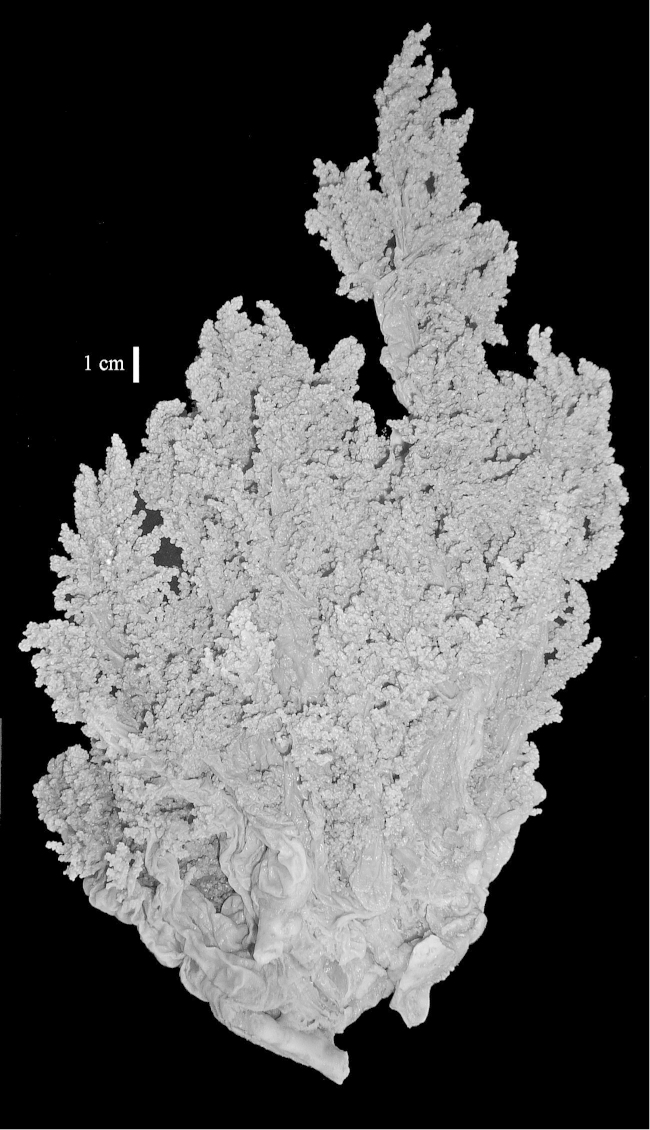
*Litophyton striatum* (Kükenthal, 1903), RMNH Coel. 8048, holotype *Nephthea galbuloides*.

**Figure 81.**
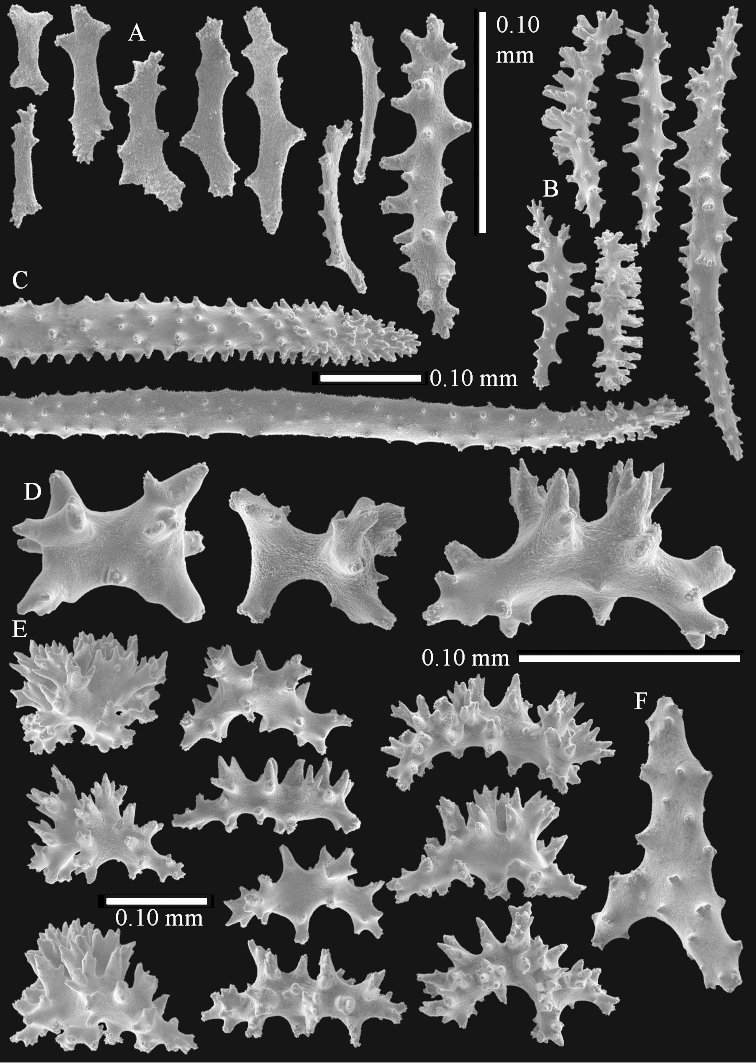
*Litophyton striatum* (Kükenthal, 1903), RMNH Coel. 8048, holotype *Nephthea galbuloides*. **A** tentacle rodlets and one small polyp body spindle **B** polyp body spindles **C** spindles of supporting bundle **D–E** sclerites of surface layer top of stalk **F** interior base stalk spindle. Scale at **C** also applies to **B**.

**Figure 82.**
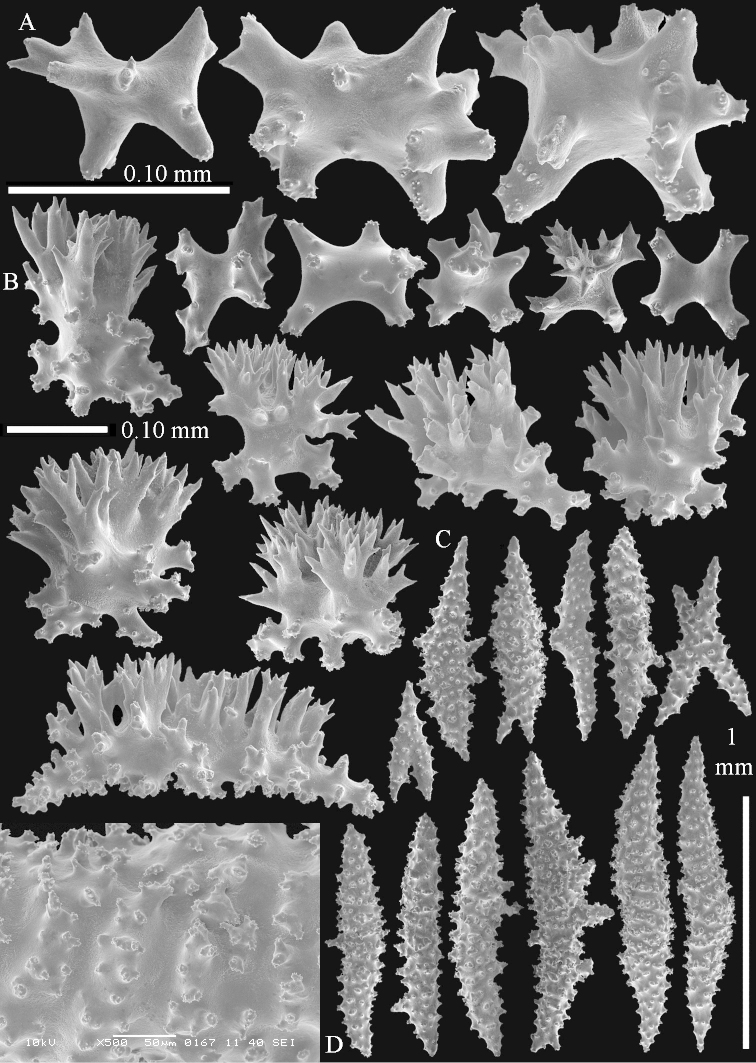
*Litophyton striatum* (Kükenthal, 1903), RMNH Coel. 8048, holotype *Nephthea galbuloides*. **A–B** sclerites surface layer base of stalk **C** spindles interior base of stalk **D** tubercles on spindle.

**Figure 83.**
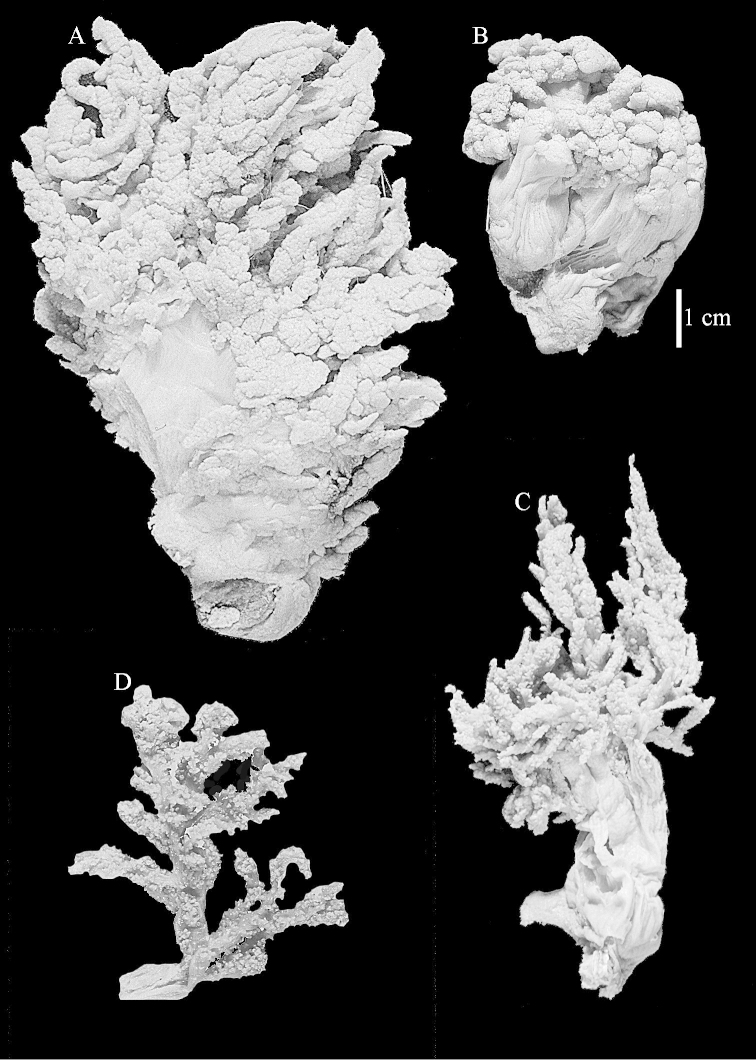
*Litophyton viridis* (May, 1898). **A** syntype ZMH 2396 **B** syntype ZMH 2397 **C** ZMH 2390, holotype *Litophyton sanderi* **D** BM 1933.3.13.193, holotype *Litophyton crosslandi*.

**Figure 84.**
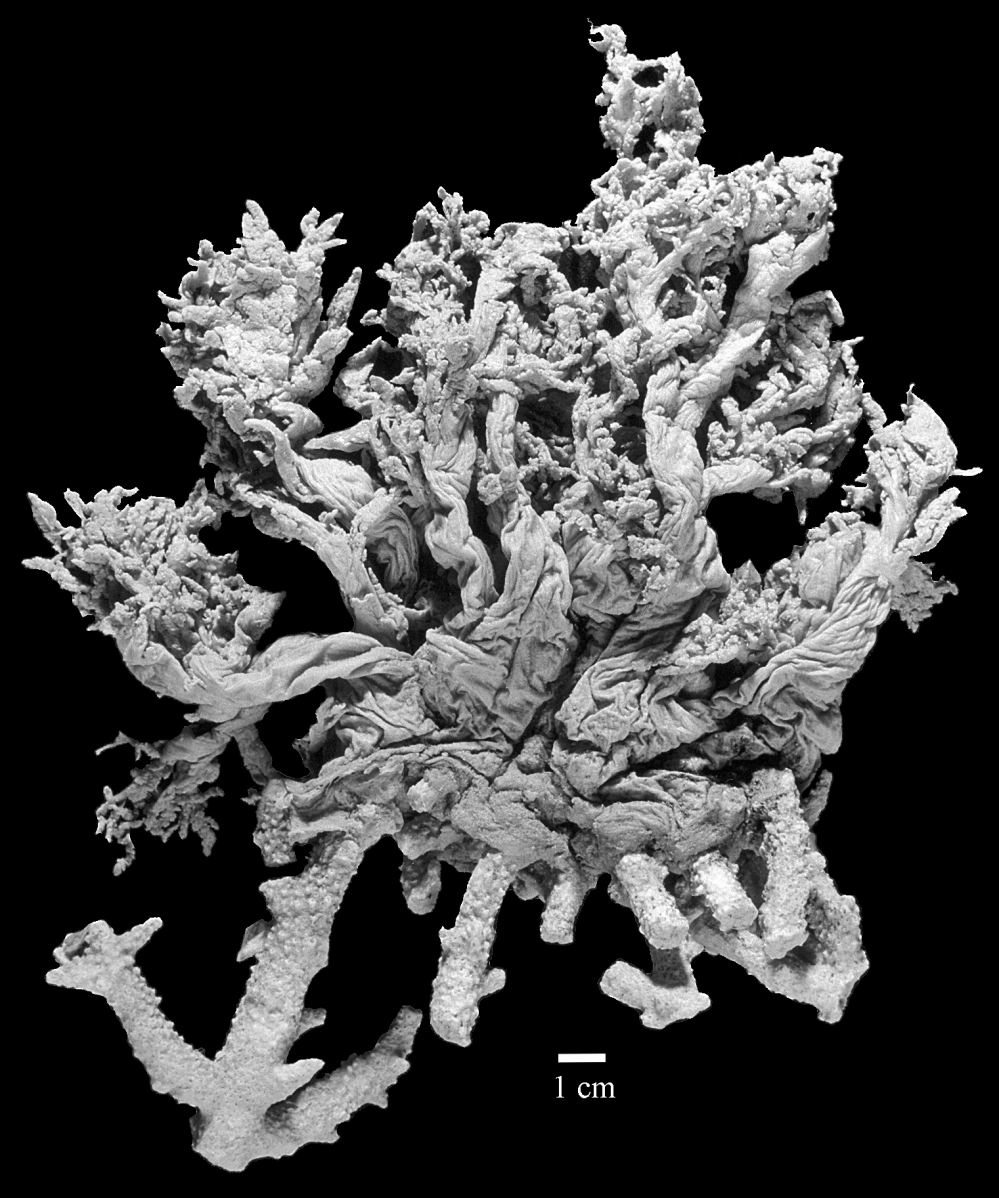
*Litophyton viridis* (May, 1898). ZMH C2391, syntype *Ammothea stuhlmanni*.

**Figure 85.**
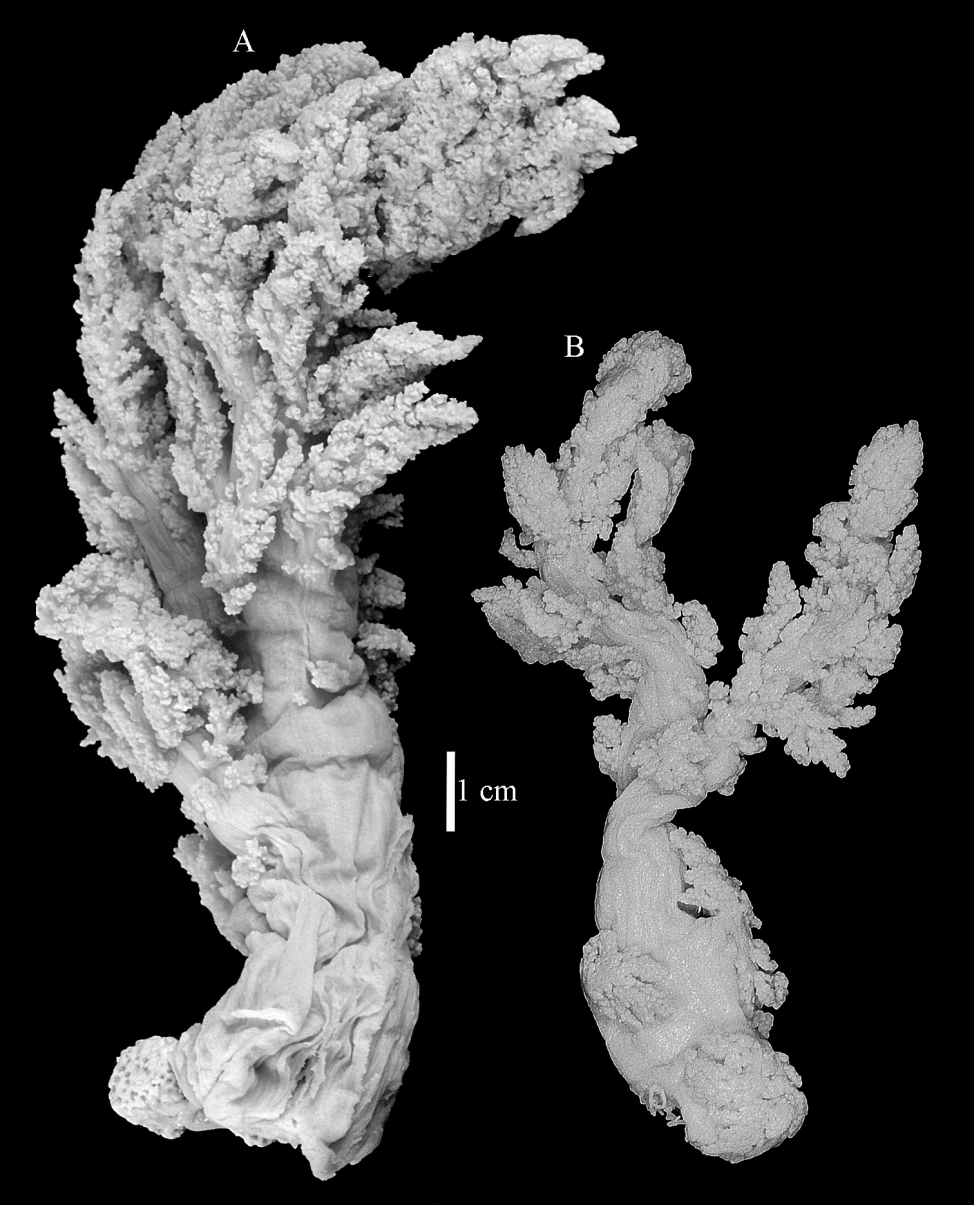
*Litophyton viridis* (May, 1898). **A** NHMW C2347, part holotype *Litophyton acutifolium* **B** ZMB 6683, part holotype *Litophyton acutifolium*.

**Figure 86.**
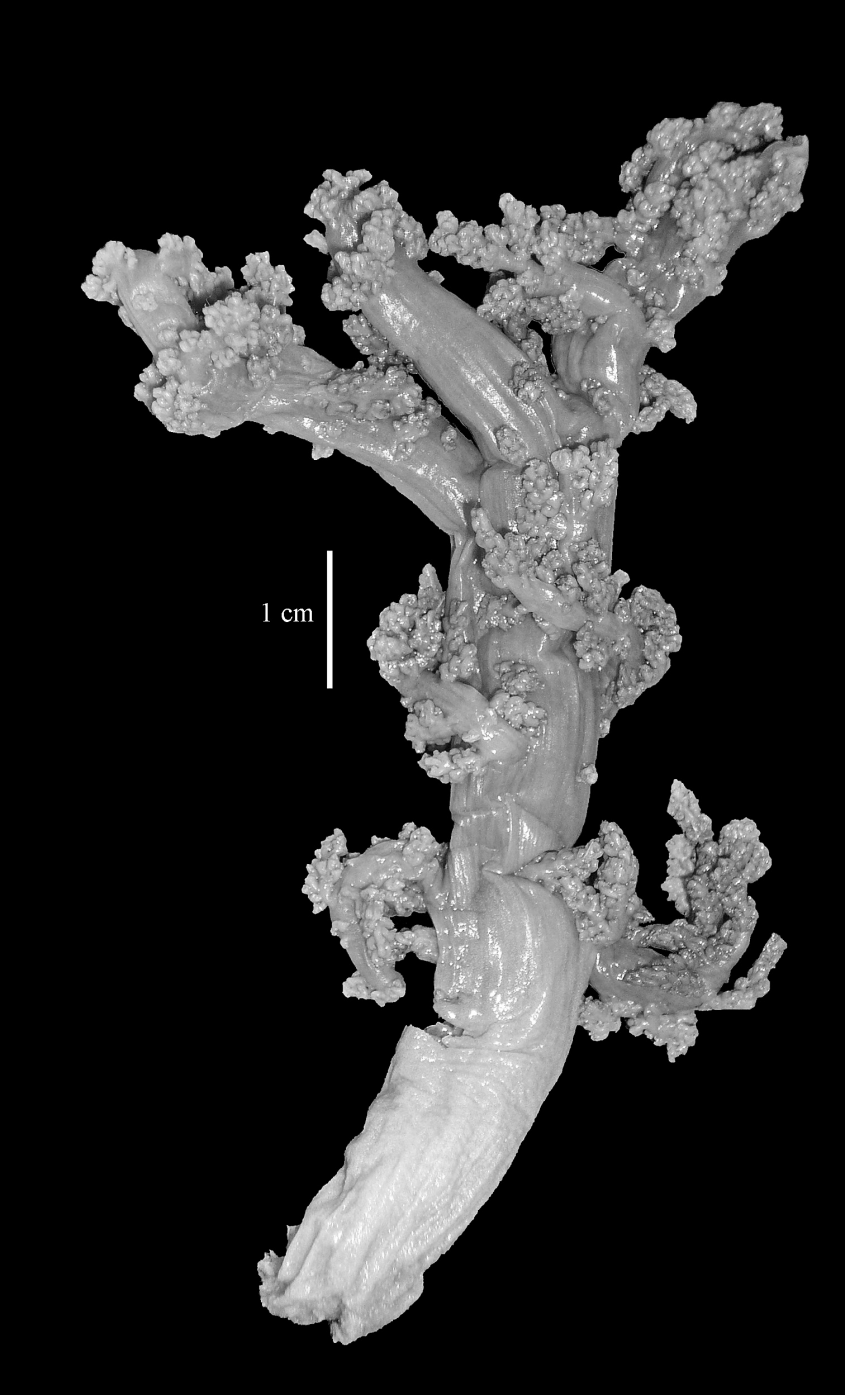
*Litophyton viridis* (May, 1898), ZMTAU Co 26193.

**Figure 87.**
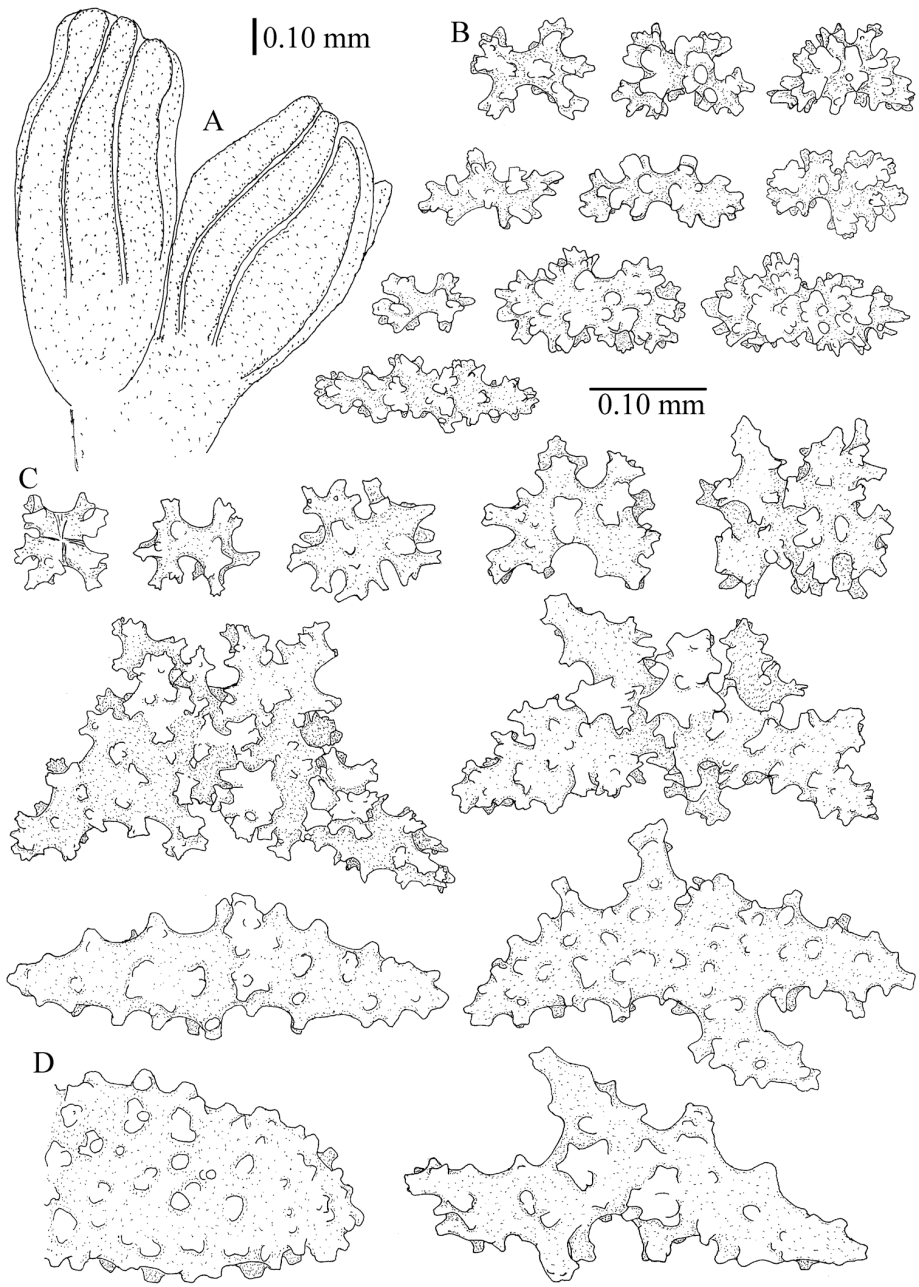
*Litophyton viridis* (May, 1898); syntype ZMH 2396. **A** polyps **B** sclerites surface layer top of stalk **C** sclerites surface layer base of stalk **D** spindles interior base of stalk. Scale at **B** also applies to **C–D**.

**Figure 88.**
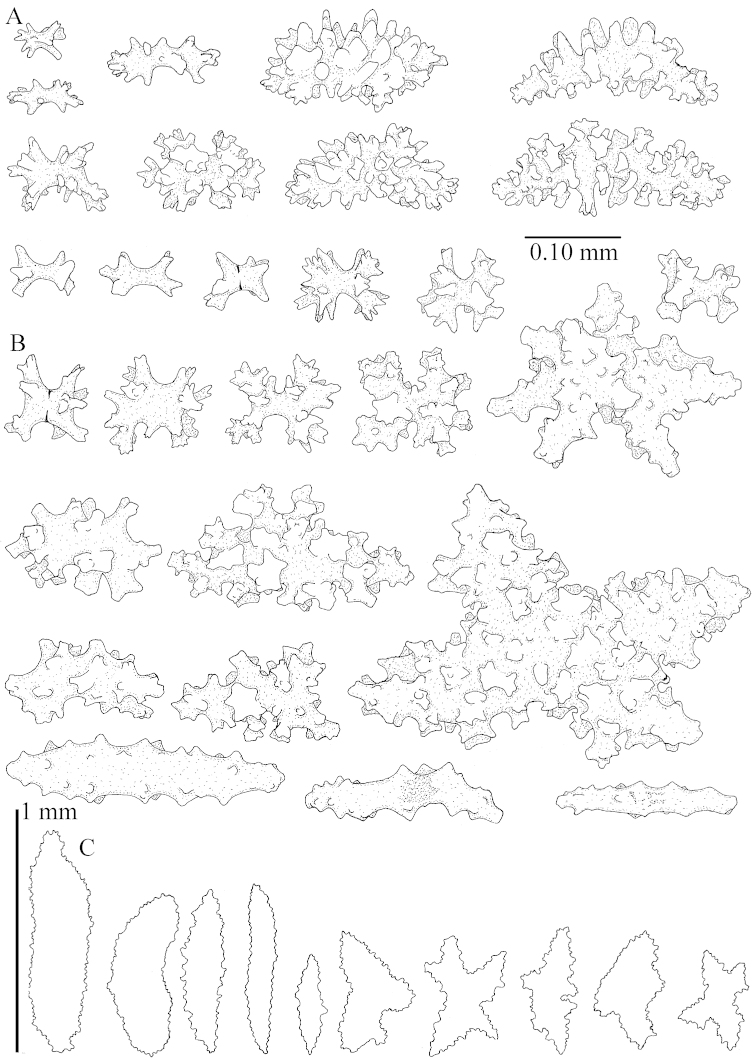
*Litophyton viridis* (May, 1898); syntype ZMH 2397. **A** sclerites surface layer top of stalk **B** sclerites surface layer base of stalk **C** spindles interior stalk.

**Figure 89.**
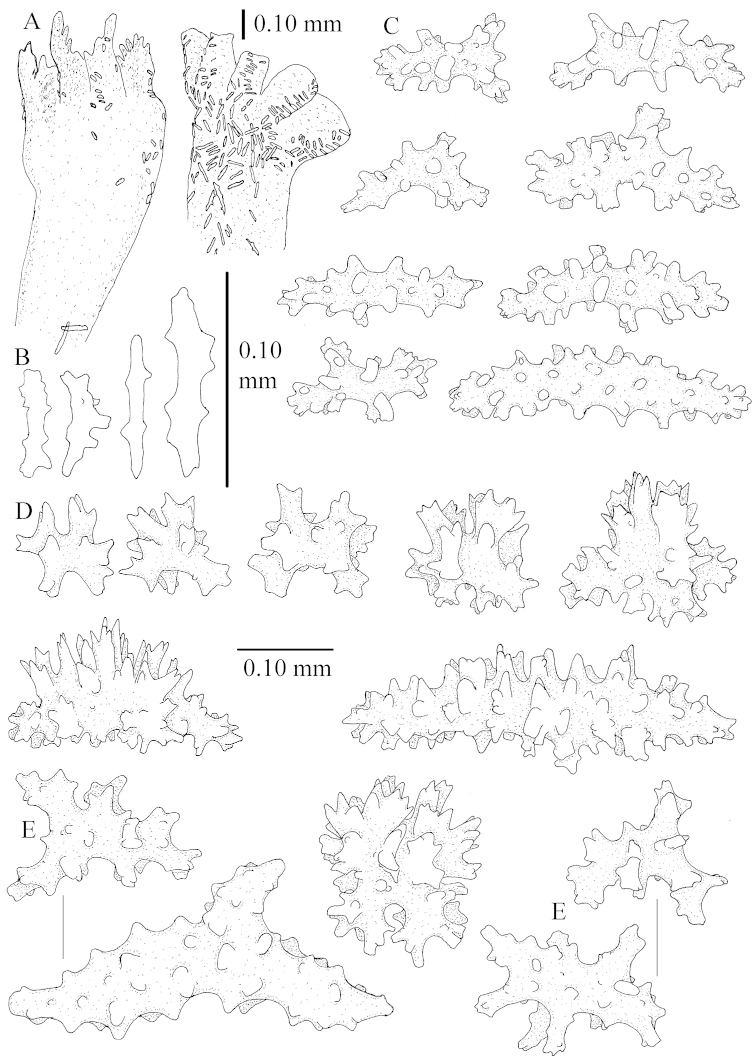
*Litophyton stuhlmanni* (May, 1898), syntype ZMH C2391. **A** lateral views of polyp armature **B** polyp sclerites **C** sclerites surface layer top of stalk **D** sclerites surface layer base of stalk **E** spindles, interior base of stalk. Scale at **D** also applies to **C** and **E**.

**Figure 90.**
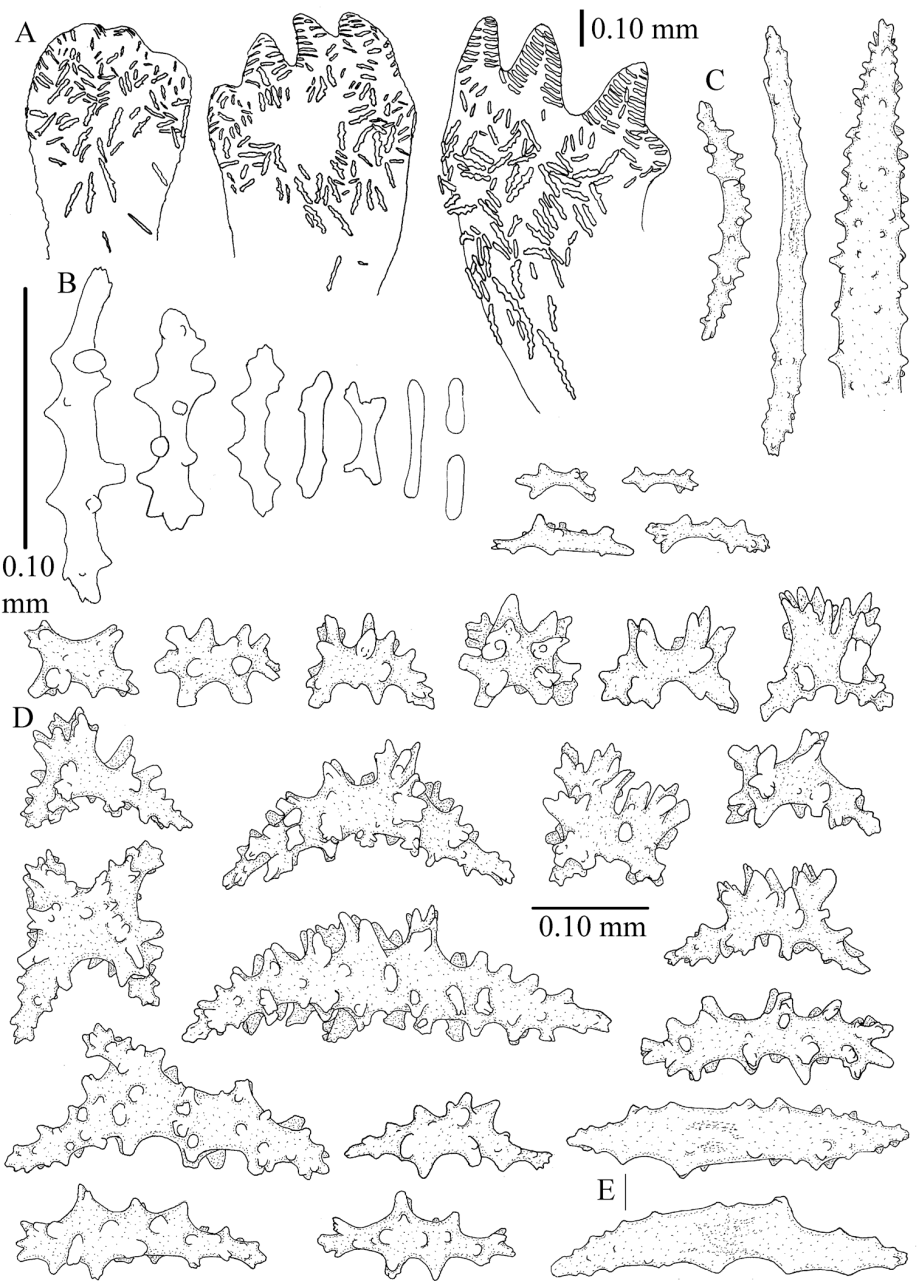
*Litophytum sanderi* (May, 1899), holotype ZMH C2390. **A** lateral views of polyp armature **B** polyp body sclerites **C** supporting bundle spindles **D** sclerites surface layer top of stalk **E** spindles interior top of stalk. Scale at **D** also applies to **C** and **E**.

**Figure 91.**
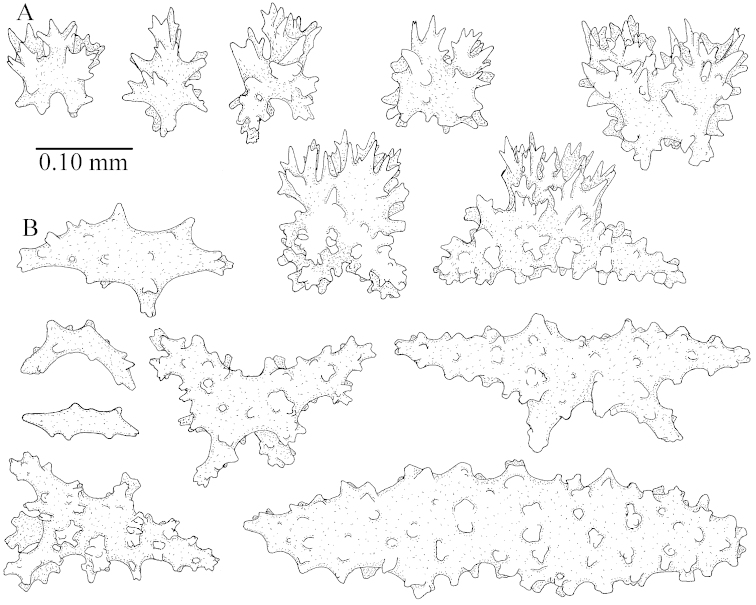
*Litophytum sanderi* (May, 1899), holotype ZMH C2390. **A** sclerites surface layer base of stalk **B** spindles interior base of stalk.

**Figure 92.**
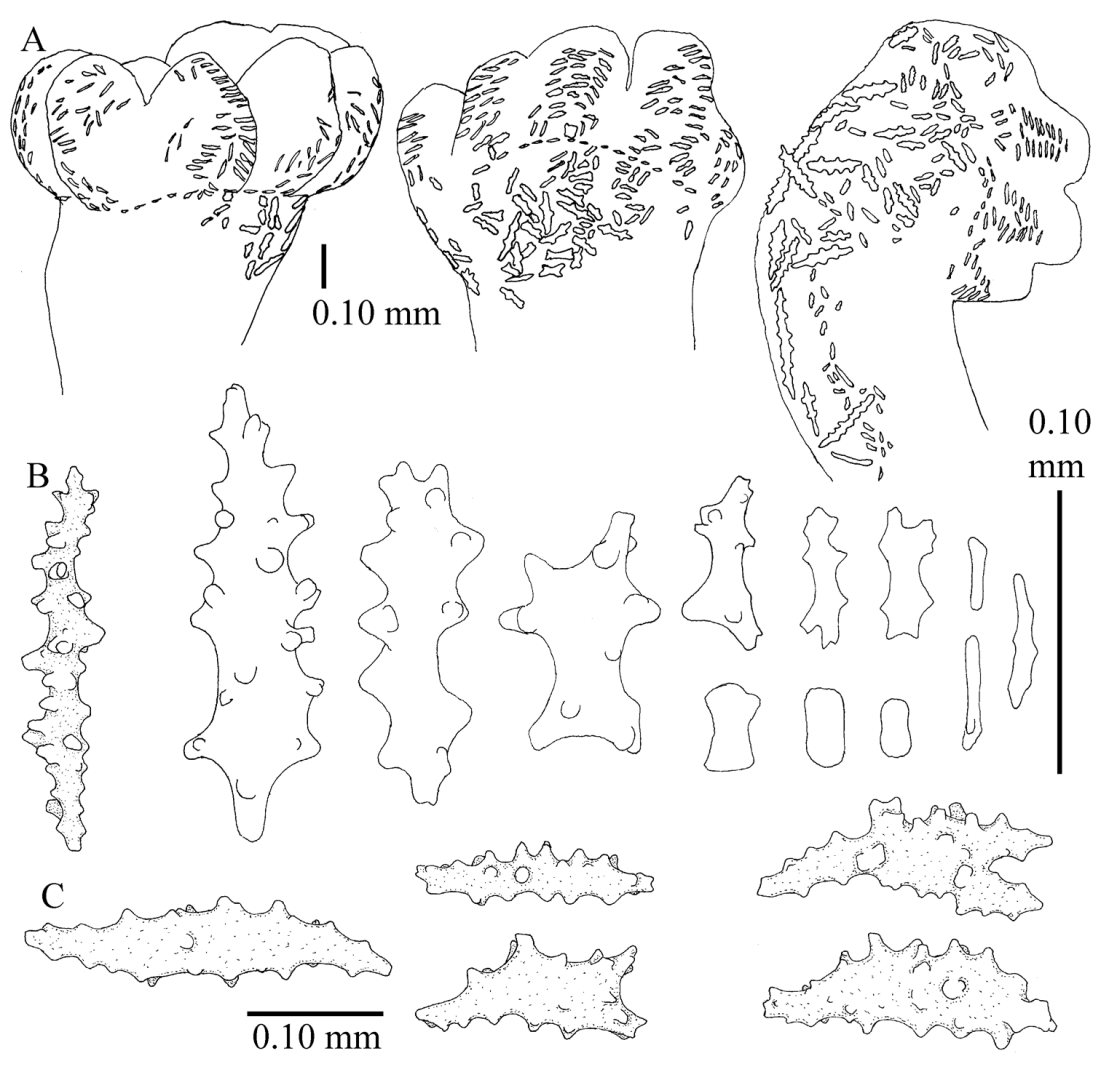
*Litophyton crosslandi* Thomson & McQueen, 1908, holotype BM 1933.3.13.193. **A** lateral views of polyp armature **B** polyp body sclerites **C** branch sclerites. Scale at **C** also applies to most left sclerite of **B**.

**Figure 93.**
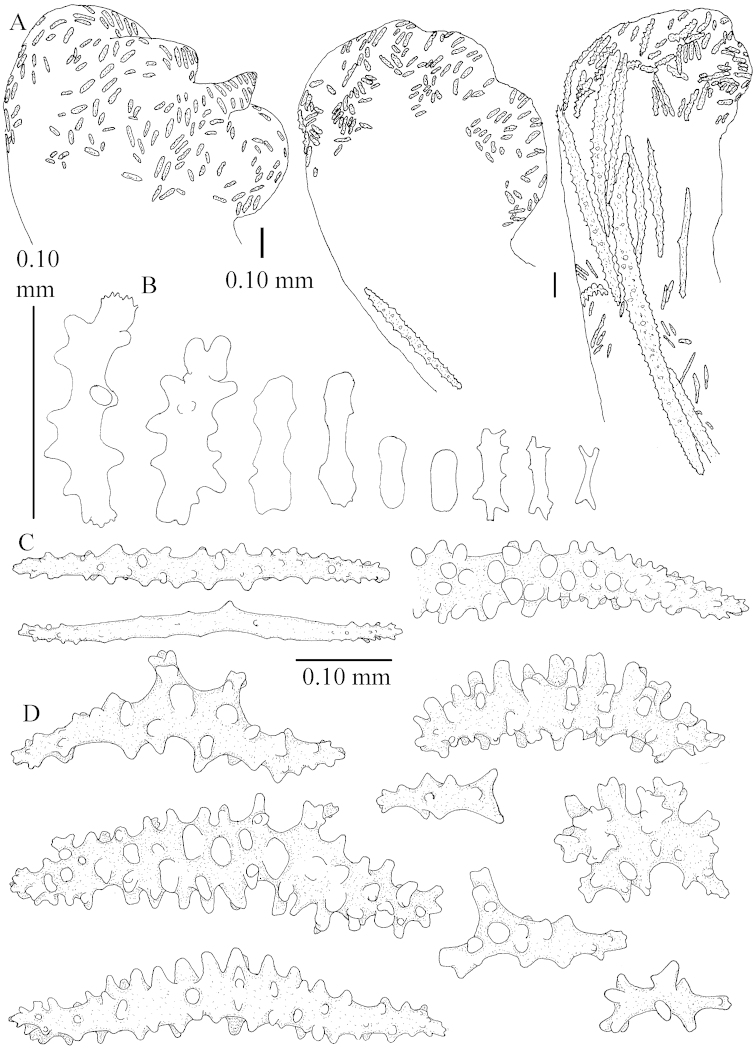
*Litophytum acutifolium* Kükenthal, 1913, ZMB 6683, part of holotype. **A** lateral views of polyp armature **B** polyp body sclerites **C** supporting bundle spindles **D** sclerites surface layer top of stalk. Scale at **C** also applies to **D**.

**Figure 94.**
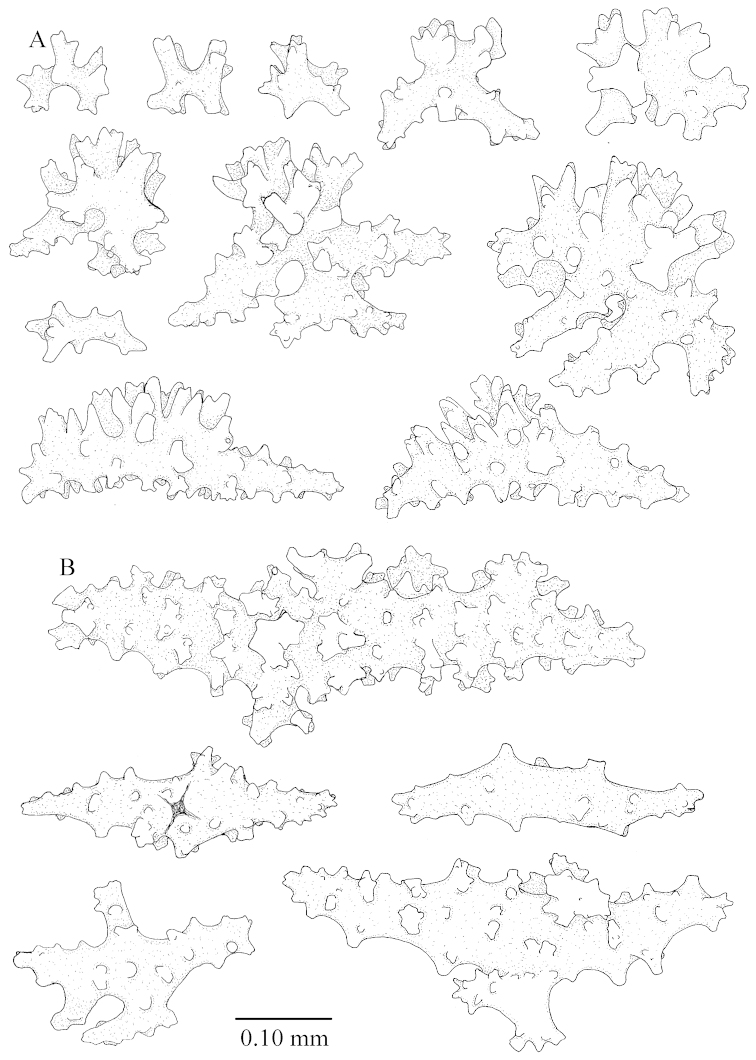
*Litophytum acutifolium* Kükenthal, 1913, ZMB 6683, part of holotype. **A** sclerites surface layer base of stalk **B** spindles interior base of stalk.

**Figure 95.**
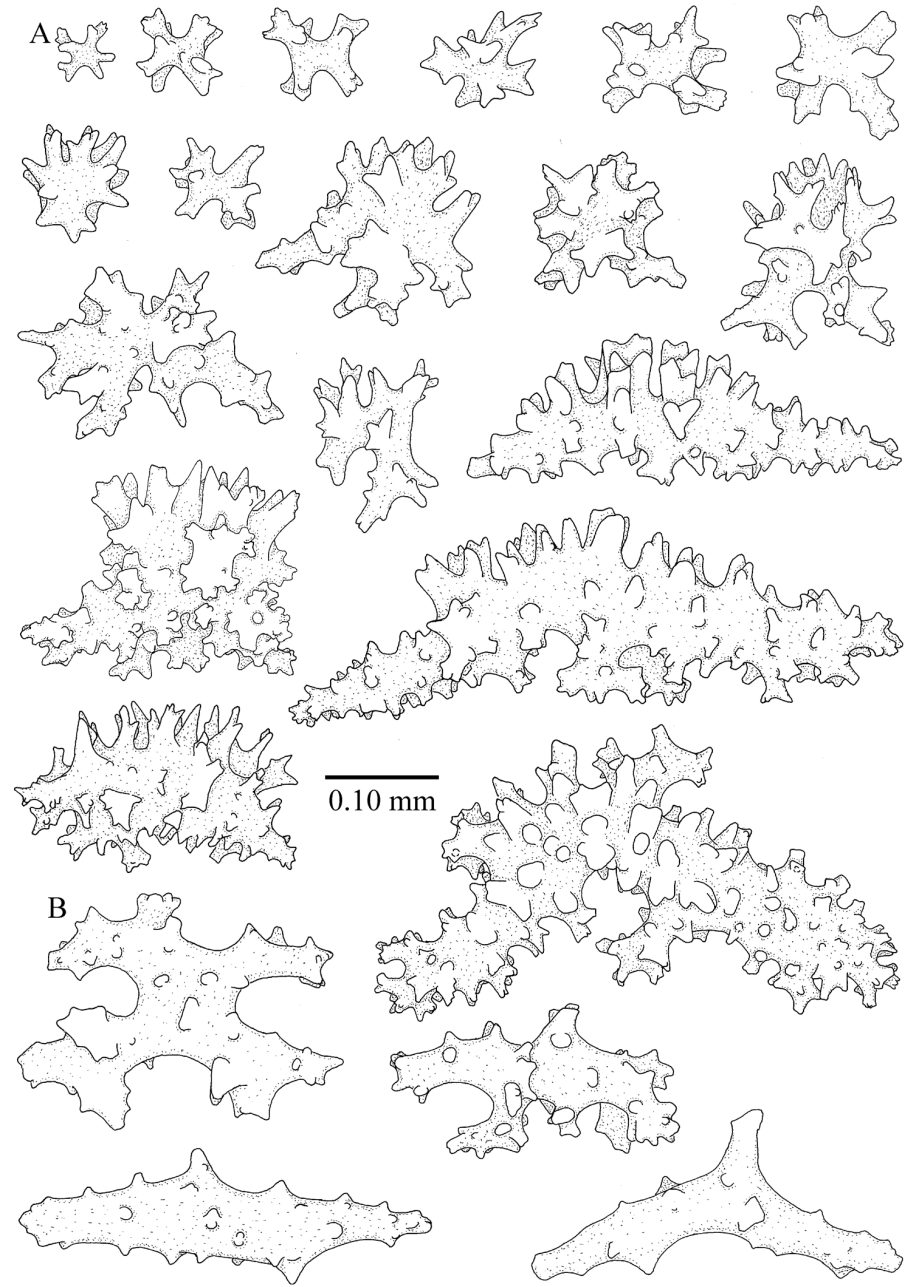
*Litophytum acutifolium* Kükenthal, 1913, NHMW C2347, part of holotype. **A** sclerites surface layer base of stalk **B** spindles interior base of stalk.

**Figure 96.**
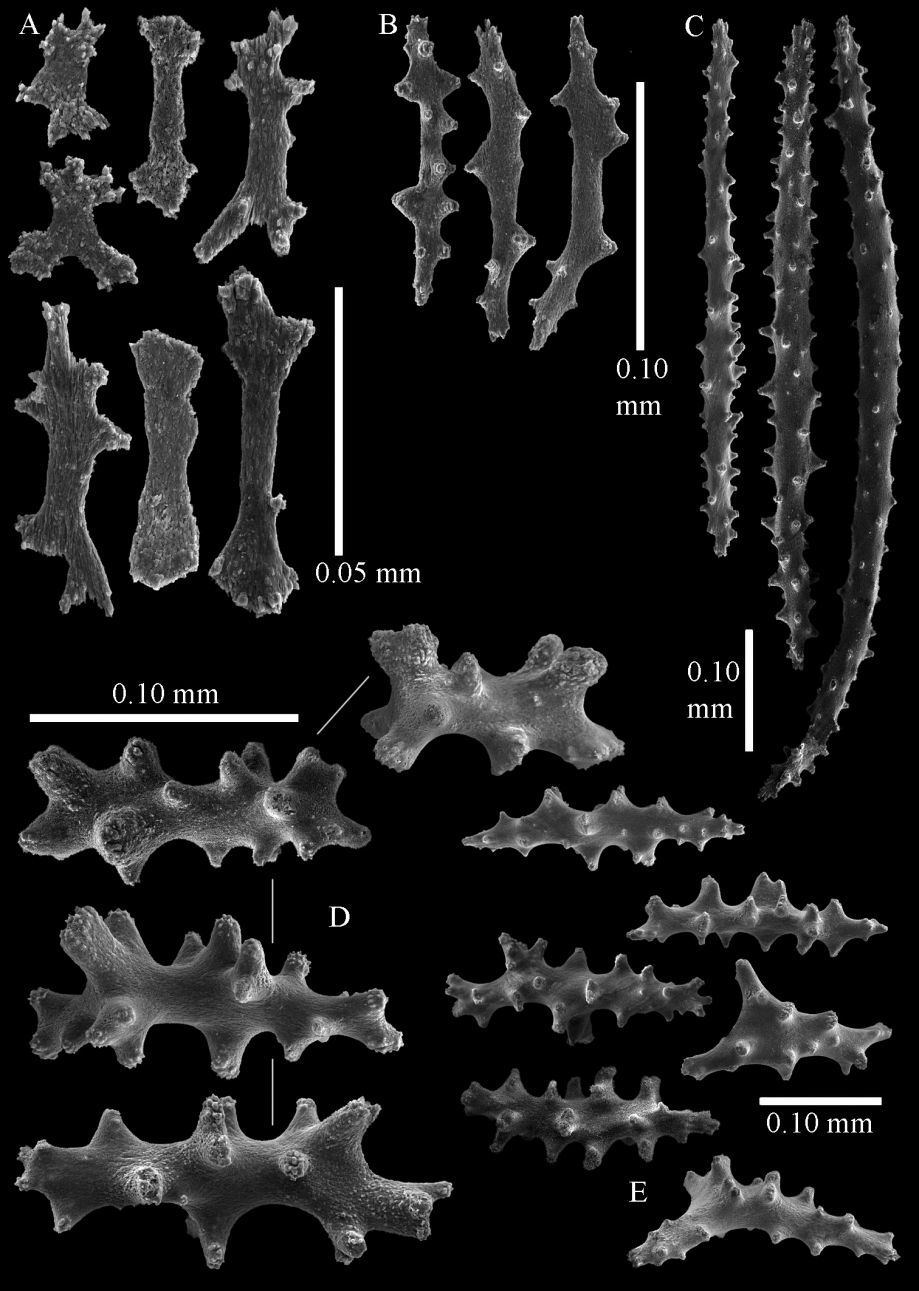
*Litophyton viridis* (May, 1898), ZMTAU Co 26193. **A** tentacle rodlets **B** polyp body spindles **C** supporting bundle spindles **D–E** sclerites surface layer top of stalk.

**Figure 97.**
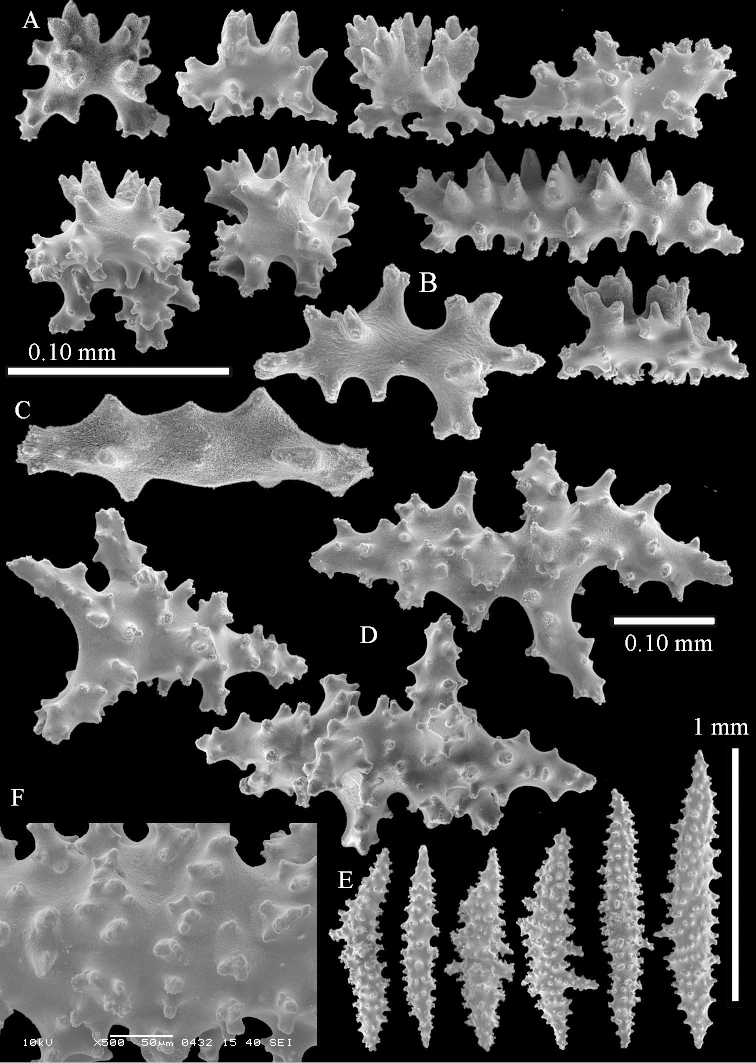
*Litophyton viridis* (May, 1898), ZMTAU Co 26193. **A–B** sclerites surface layer base of stalk **C–E** spindles interior base of stalk **F** detail tuberculation of interior spindle.

### Unidentified specimens

ZMTAU Co 25672 1894, Red Sea, South tip Sinai, Shab Mahmud, beacon rock, depth 0–20 m, 12 July 1987, coll. Y. Benayahu; disintegrated sclerites.

ZMTAU Co 26081, Red Sea, South tip Sinai Ras um Sud, 9 October 1988, coll. Y. Benayahu; stalk missing.

ZMTAU Co 26255, Red Sea, South tip Sinai, Ras Zaatir, 10 October 1989, coll. Y. Benayahu; consists of seven fragments all lacking a stalk.

ZMTAU Co 28609, Red Sea, Gulf of Aqaba, Eilat (Marin Lab.), depth 3 m, 1 August 1984, coll. Y. Benayahu; disintegrated sclerites.

ZMTAU Co 33090, Israel, Gulf of Aqaba, nature reserve, May 2000, coll. Y. Benayahu; fragments of branches, no internal sclerites found.

ZMTAU Co 25827 1460, Red Sea, South tip Sinai, Shab el Utaf, depth 10 m, 8 July 1986, coll. Y. Benayahu; base missing.

ZMTAU Co 26238, 4 small colonies, Red Sea, Gulf of Aqaba Wadi Magrash km 207, 17 April 1979, coll. Y. Benayahu; large supporting bundle spindles unlike other species, base looks a bit like *Litophyton maldivensis*.

## Supplementary Material

XML Treatment for Litophyton

XML Treatment for Litophyton acuticonicum

XML Treatment for Litophyton arboreum

XML Treatment for Litophyton bumastum

XML Treatment for Litophyton chabrolii

XML Treatment for Litophyton curvum

XML Treatment for Litophyton filamentosum

XML Treatment for Litophyton laevis

XML Treatment for Litophyton lanternarium

XML Treatment for Litophyton maldivensis

XML Treatment for Litophyton ?savignyi

XML Treatment for Litophyton simulatum

XML Treatment for Litophyton striatum

XML Treatment for Litophyton viridis
